# Early antenatal care visit: a systematic analysis of regional and global levels and trends of coverage from 1990 to 2013

**DOI:** 10.1016/S2214-109X(17)30325-X

**Published:** 2017-09-11

**Authors:** Ann-Beth Moller, Max Petzold, Doris Chou, Lale Say

**Affiliations:** a World Health Organization, Geneva, Switzerland; b Health Metrics Unit, Sahlgrenska Academy, University of Gothenburg, Gothenburg, Sweden

## Abstract

**Background:**

The timing of the first antenatal care visit is paramount for ensuring optimal health outcomes for women and children, and it is recommended that all pregnant women initiate antenatal care in the first trimester of pregnancy (early antenatal care visit). Systematic global analysis of early antenatal care visits has not been done previously. This study reports on regional and global estimates of the coverage of early antenatal care visits from 1990 to 2013.

**Methods:**

Data were obtained from nationally representative surveys and national health information systems. Estimates of coverage of early antenatal care visits were generated with linear regression analysis and based on 516 logit-transformed observations from 132 countries. The model accounted for differences by data sources in reporting the cutoff for the early antenatal care visit.

**Findings:**

The estimated worldwide coverage of early antenatal care visits increased from 40·9% (95% uncertainty interval [UI] 34·6–46·7) in 1990 to 58·6% (52·1–64·3) in 2013, corresponding to a 43·3% increase. Overall coverage in the developing regions was 48·1% (95% UI 43·4–52·4) in 2013 compared with 84·8% (81·6–87·7) in the developed regions. In 2013, the estimated coverage of early antenatal care visits was 24·0% (95% UI 21·7–26·5) in low-income countries compared with 81·9% (76·5–87·1) in high-income countries.

**Interpretation:**

Progress in the coverage of early antenatal care visits has been achieved but coverage is still far from universal. Substantial inequity exists in coverage both within regions and between income groups. The absence of data in many countries is of concern and efforts should be made to collect and report coverage of early antenatal care visits to enable better monitoring and evaluation.

**Funding:**

Department of Reproductive Health and Research, WHO and UNDP/UNFPA/UNICEF/WHO/World Bank Special Programme of Research, Development and Research Training in Human Reproduction.

## Introduction

The health of women and children remains an unfinished agenda and a global challenge. Efforts and investments are needed to sustain and accelerate progress if countries and the international community are to prevent maternal and child morbidity and reach the related Sustainable Development Goals (SDGs).^1^, ^2^, ^3^

Antenatal care is defined as the routine care of pregnant women provided between conception and the onset of labour. Antenatal care is an opportunity to provide care for prevention and management of existing and potential causes of maternal and newborn mortality and morbidity. The new WHO antenatal care model recommends that the first antenatal care visit takes place within the first trimester (ie, gestational age of <12 weeks) and an additional seven visits are recommended.^4^

The timing of initiation of the first antenatal care visit is paramount for ensuring optimal care and health outcomes for women and children. Globally, there has been a change in the pattern and type of obstetric outcomes, as a greater proportion of deaths and morbidities are related to complications of pre-existing medical conditions, namely indirect conditions, in a phenomenon described as the obstetric transition.^5^

An early antenatal care visit gives the opportunity to provide screening and tests that are most effective early in the pregnancy (ie, correct assessment of gestational age to allow for accurate treatment of preterm labour, screening for genetic and congenital disorders, provision of folic acid supplementation to reduce the risk of neural tube defects, and screening and treatment for iron deficiency anaemia and sexually transmitted infections). Additionally, the visit can potentially capture non-communicable diseases such as diabetes and provide guidance on modifiable lifestyle risks such as smoking, alcohol consumption, drug abuse, obesity, malnutrition, and occupational exposures.^6^, ^7^ All these conditions can be detected and treated if early, timely, and high-quality antenatal care is provided, but beyond the content the antenatal care services need to be available, accessible, and acceptable.

The SDG targets 3.1 (by 2030 reduce the global maternal mortality ratio to less than 70 per 100 000 livebirths) and 3.2 (by 2030, end preventable deaths of newborns and children under 5 years of age, with all countries aiming to reduce neonatal mortality to at least as low as 12 per 1000 livebirths and under-5 mortality to at least as low as 25 per 1000 livebirths)^8^ are supported by several global initiatives and strategies such as the Global Strategy for Women's, Children's and Adolescents' Health 2016–2030,^9^ the Global Financing Facility in Support of Every Woman Every Child,^10^ the Strategies toward Ending Preventable Maternal Mortality,^2^ and Every Newborn: An Action Plan to End Preventable Deaths.^3^ Thus, it is important to ensure coverage of early antenatal care services starting from the first trimester as one component to achieve these targets.

**Research in context**

**Evidence before this study**

Little evidence is available about early antenatal care visit coverage globally. Only two agencies have synthesised national data on such coverage: ICF International published an analysis in 2011 (using only Demographic and Health Surveys [DHS] data from 2003 to 2008) and WHO/UNICEF published an analysis in 2003 (using only DHS data from 1992 to 1999) limited to low-income and middle-income countries. Before our study, no previous (comparable) global or regional estimates existed. In our search of the scientific literature, population-based health surveys, health information system reports, and perinatal studies were included if national level data from 1990 to 2013 on early antenatal care were available. Specific information was provided in relation to which weeks or month(s) the pregnant women had their first antenatal care visit, as well as specification of data coverage period (also referred to as re-call period) and data collection period specified. The searches were done from March 7, to March 31, 2016.

No previous global and regional estimates of early antenatal care coverage have been made. The timing of initiation of the first antenatal care contact is paramount for ensuring optimal health outcomes for women and children, and it is recommended by WHO that pregnant women initiate their first antenatal care visit in the first trimester of pregnancy. Early antenatal care coverage was not tracked under the Millennium Development Goals, which instead focused on coverage of at least one antenatal care visit and coverage of at least four antenatal care visits, and is not included in the Sustainable Development Goals (SDGs) and in the indicator and monitoring framework for the Global Strategy for Women's, Children's and Adolescents' Health, 2016–30. However, new WHO guidelines on antenatal care emphasise the importance of early antenatal care contact, which highlights the need to promote enhanced within-country and global monitoring and evaluation of early antenatal care coverage.

**Added value of this study**

Through systematic searches on ministries of health and national statistics office webpages, as well as national representative household surveys such as the DHS, MICS, RHS, and other national representative surveys, we compiled available national data on early antenatal care coverage. The final dataset included 516 data points from 132 countries. We estimated global and regional levels and trends of coverage of early antenatal care, and we also noted the inequality of coverage of early antenatal care across regions and income groups.

**Implications of all available evidence**

The SDGs seek to reduce inequalities. With respect to the vision of ending preventable maternal deaths and preventable deaths of newborns and children under 5 (targets 3.1 and 3.2), urgent action is needed to ensure that all pregnant women initiate their first antenatal contact during the first trimester of pregnancy and receive the optimal care and treatment throughout the pregnancy and childbirth. 62 countries did not have any data of early antenatal care coverage and some countries had very few data points. Progress in early antenatal care coverage has been achieved but the level of coverage is still low. The absence of data in many countries is of concern and efforts should be made to collect and report coverage of early antenatal care to enable better monitoring and evaluation.

The initiation of antenatal care in early pregnancy is assessed in population-based surveys such as Demographic and Health Surveys (DHS)^11^ and Multiple Indicator Cluster Surveys (MICS, round 5 only)^12^ and other national household surveys, collected by routine health management information systems in high-income countries and in special perinatal surveys. Global estimates of the coverage of early antenatal care visits, combined with the content and quality of antenatal care, are crucial metrics for every programme and policy aiming to improve maternal and child health at both the local and global levels.^13^ Since little evidence has been collected on the coverage of early antenatal care visits globally, our objective was to examine and assess trends in coverage of early antenatal care visits at the global and regional level from 1990 to 2013.

## Methods

### Reporting rationale and data sources

We followed the Guidelines for Accurate and Transparent Health Estimates Reporting (GATHER) statement in developing the database, analysis, and presentation of the study (appendix pp 3, 4).^14^

We did a search to identify national-level data on coverage of early antenatal care visits (appendix p 5). The sources can be divided into two main categories: routine management health information system reports from ministries of health (MoHs) and official publications from MoHs or national statistics offices (NSOs); and population-based household surveys such as DHS,^11^ MICS round 5,^12^ Reproductive Health Surveys (RHS),^15^ other national surveys (eg, Family Health Surveys), and special national perinatal studies.^16^

All MoH and NSO websites were systematically searched for all 194 WHO Member States without any language restrictions. MoH and NSO websites were identified with Google searches applying the search terms “[country name] ministry of health”. To identify data on early antenatal care visit on the MoH websites, we searched pages using the search terms “annual MoH reports”, “annual MoH statistics reports”, and “perinatal and reproductive health statistics”, and used the website search functions for terms such as “antenatal care”, “prenatal”, “reproductive health”, “perinatal”, “women's health”, and “mothers and babies”. NSOs were identified from the UN website, which provides links to all NSOs. The same search approach was used for NSO websites as described for the MoH websites. We selected the search terms after first testing them on several MoH and NSO webpages; these terms gave the best results given that many of these webpages cannot support complex searches.

Population-based household surveys such as the DHS were obtained online from ICF International,^11^ the MICS round 5 surveys from UNICEF,^12^ and the RHS from the National Center for Chronic Disease Prevention and Health Promotion (NCCDPHP).^15^ Additionally, a hand search was done using Google to identify additional RHS (not all available RHS are included on the NCCDPHP website) and some DHS published at the national level (eg, those from Turkey and Timor-Leste).

Perinatal surveys were obtained from the Euro-Peristat webpage,^16^ and a hand search of Google was done for perinatal studies from developed countries outside Europe using the search terms “perinatal study”, “perinatal survey”, “maternity”, and “maternity care”.

### Data inclusion and quality

Population-based national household surveys, health information system reports, and perinatal studies that reported national-level data on the early antenatal care visit from 1990 to the latest date available (March, 2016) were included if they specified information related to the week or months at which the pregnant women had their first antenatal care visit, and the data coverage period (also referred to as the re-call period). A-BM did the searches and A-BM and DC assessed studies for eligibility. We checked for double counting of the data.

No well established tool for standardised appraisal of public health information systems has been developed and applied.^17^ Data from developing countries were mainly retrieved from the DHS programme and the MICS programme. The DHS programme implemented by ICF International started in 1984^11^ and has received a worldwide reputation for collecting and disseminating nationally representative data on maternal and child health from more than 300 surveys in over 90 countries. The MICS programme implemented by UNICEF^12^ has done surveys since 1995 mainly in countries not covered under the ICF International programme and is an important source of comparable data; more than 280 surveys have been implemented in 100 low-income and middle-income countries, although data on the early antenatal care visit have only been collected since the last round of surveys (round 5). These surveys are considered the best available way to obtain data on several types of maternal health indicators in developing countries.^13^

The regional estimates were developed using the Millennium Development Goal (MDG) regions (appendix pp 8, 9),^18^ because no new regional grouping has yet been suggested for monitoring of the SDGs. To examine income inequalities, the estimates are also presented by World Bank Group income groups (appendix p 10).^19^

For the purpose of these estimates we defined the coverage of early antenatal care visits as: the proportion of women aged 15–49 years with a livebirth in a given time period who initiated their first antenatal care visit in the first trimester (less than 14 weeks' gestation) in the same time period (panel). Sources reporting early antenatal care visits after 14 weeks of gestation were also included but adjusted and accounted for accordingly in the statistical model. We do not apply the new term “contact” as used in the new WHO 2016 antenatal care guidelines^4^ since currently available data are collected and measured as visits.

Panel **Definition of coverage of early antenatal care visits**

Coverage of early antenatal care visits was defined in this study as the proportion of women aged 15–49 years with a livebirth in a given time period who initiated their first antenatal care visit in the first trimester (<12 weeks' gestation) in the same period. The WHO International Statistical Classification of Diseases and Related Health Problems, 10th revision (ICD-10) defines the first pregnancy trimester as less than 14 weeks and 0 days counted from the first day of the last menstrual period.

The gold standard for the assessment of gestational age is routine early ultrasound assessment together with fetal measurements, ideally in the first trimester. Gestational assessment based on the date of last menstrual period was previously the most widespread method used and remains the only available method in many settings. This method assumes that conception occurs on the same day as ovulation (14 days after the onset of the last menstrual period). It has low accuracy due to considerable variation in the length of the menstrual cycle among women, the fact that conception can occur up to several days after ovulation, and the recall of the date of last menstrual period being subject to errors. Many countries now use the best obstetric estimate, combining ultrasound and last menstrual period as an approach to estimating gestational age.

Ten different cutoffs were used to report antenatal care visits in the dataset identified for this study. These were divided into two groups according to the ICD-10 classification of first and second trimester. Group 1 included women with a visit in their first trimester (<14 weeks' gestation, early antenatal care visit): the cutoffs reported were less than 3 months, first trimester, less than 12 weeks, less than 13 weeks, and less than 14 weeks. Group 2 included women with a visit in their second trimester (>14 weeks' gestation, late antenatal care visit): the cutoffs reported were less than 15 weeks, less than 16 weeks, less than 20 weeks, less than 4 months, and less than 6 months.

For sources reporting different initiation of first visit, data were extracted and adjusted according to the ICD-10 definition of first trimester before analysis.

### Statistical analysis

The outcome was the logit transformation of the different early antenatal care estimates. The logit transformation fit the data well from graphical assessment on a country level. Use of the logit transformation ensures that the predicted levels were within the possible range of 0–100%. The countries had 1–27 observations, with an average of 3·9 observations over the years 1990–2013. Statistical regression analyses were done in Stata version 14.2 and were based on the full dataset (516 observations). No weights for study sample size were applied, and no data were imputed for countries with no data. In the regression models, the MDG regional grouping was used (appendix p 8). In an initial analysis the mean difference in proportion between studies reporting early antenatal visit (before 14 weeks' gestational age) and later initiation (after 14 weeks' gestational age) was estimated. Later initiation is expected to be higher since it covers a larger period of the gestational age. An overview of the ten different cutoffs used for reporting early antenatal care visits in the dataset is shown in the panel. These cutoffs were divided into two groups according to the ICD-10 classification of first and second trimester.^20^ Studies reporting early antenatal care visit coverage for late initiation (after 14 weeks' gestational age) were estimated to have 2·75 percentage points higher antenatal care coverage. Since the chosen standard definition is the early antenatal care visit, studies reporting later initiation were standardised accordingly by subtracting the estimated difference value from the original reported level.

In the main analysis, global and regional levels and trends were estimated. The logit transform of the standardised early antenatal care data (ie, the relative increase) was estimated using a linear Bayesian model (bayesmh command in Stata):

$$\text{Logit}\left({\text{Prop}}_{ijk}\right)\;=\;\alpha_{j}\;+\;\beta_{j}{\text{X}}_{i}\;+\;\gamma_{k}\;+\;{ɛ}_{ijkl}$$

for the *i:th* year, *j:th* country, *k:th* region and the *l:th* observation. The random components α_*j*_ and β_*j*_ are the correlated country intercept and time slopes, respectively, γ_*k*_ is the regional dummy intercept, and ɛ is the residual. A burn-in period of 2500 samples and a Markov chain Monte Carlo sample size of 10 000 were used. Technical details, including the distribution of priors, are shown in the appendix (p 12). Non-informative priors were used. The back-transformed predicted country levels per year were then weighted into global and regional (MDG and World Bank Group income groups) mean values per year based on total number of livebirths in 2015 per country.^21^ Livebirths were chosen as the denominator because the numbers of pregnancies were only available for very few countries, even though it is the appropriate denominator. Only countries with at least one observation were included. 95% uncertainty intervals (UIs) were constructed from the weighted 2·5 and 97·5 percentiles of the posterior samples.

### Role of the funding source

The funder of the study had no role in study design, data collection, data analysis, data interpretation, or writing of the report. The corresponding author had full access to all the data in the study and had final responsibility for the decision to submit for publication.

## Results

The final dataset included 516 observations from 132 countries (figure). 324 (63%) observations were from population-based household surveys, 175 (34%) were from administrative records, and 17 (3%) were from perinatal studies. For 62 countries, no data were available (appendix p 13). Source details for all observations are in the appendix (pp 14–72).

**Figure:**
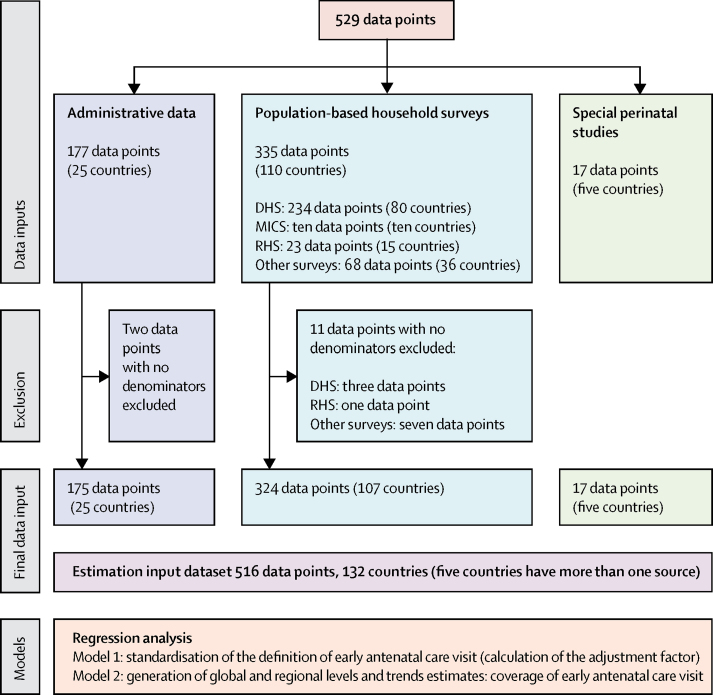
Data selection and analysis DHS=Demographic and Health Surveys. MICS=Multiple Indicator Cluster Surveys. RHS=Reproductive Health Surveys.

Worldwide, the estimated coverage of early antenatal care visits based on 183 countries increased from 40·9% (95% UI 34·6–46·7) in 1990 to 58·6% (52·1–64·3) in 2013, corresponding to a 43·3% increase (table 1). An estimated increase of more than 50% in early antenatal care visit coverage from 1990 to 2013 was achieved in five regions: Northern Africa, Western Asia, Southern Asia, developing regions, and South-eastern Asia. The lowest estimated increases in coverage between 1990 and 2013 were Latin America and the Caribbean (11·5%), developed regions (9·6%), and Eastern Asia (9·6%). These regions were also the regions with the highest estimated coverage of early antenatal care visits in 1990. Oceania was the only region for which a decrease in coverage was estimated. The estimates suggest an almost 31% decrease from 1990 to 2013.

**Table 1:** Global and regional estimates of coverage of early antenatal care visits by UN Millennium Development Goal region between 1990 and 2013

	**Early antenatal care visit coverage (%, 95% uncertainty interval)**				**Change between 1990 and 2013 (%)**
	1990	2000	2005	2013	
World	40·9% (34·6–46·7)	48·6% (45·1–51·9)	52·6% (48·7–56·3)	58·6% (52·1–64·3)	43·3%
Developed regions	76·4% (68·6–82·8)	81·7% (78·3–84·4)	83·4% (81·1–85·5)	84·8% (81·6–87·7)	9·6%
Developing regions	27·7% (25·1–31·3)	36·1% (34·3–37·9)	40·8% (38·3–43·3)	48·1% (43·4–52·4)	73·5%
Northern Africa	27·5% (21·1–35·2)	45·6% (41·0–50·4)	56·0% (50·4–61·8)	70·4% (61·9–78·6)	156·0%
Sub-Saharan Africa	17·7% (15·1–21·0)	19·9% (18·5–21·4)	21·5% (20·2–22·8)	24·9% (22·6–27·2)	40·4%
Eastern Asia	88·0% (79·6–82·4)	92·9% (90·9–94·3)	94·6% (93·7–95·3)	96·5% (95·5–97·3)	9·6%
Southern Asia	22·0% (15·8–29·5)	32·6% (28·5–36·9)	39·1% (33·1–45·4)	50·0% (38·2–61·4)	127·9%
South-eastern Asia	44·9% (38·0–52·0)	59·7% (56·1–63·2)	66·9% (62·9–70·7)	76·4% (70·1–81·5)	70·1%
Western Asia	31·1% (25·9–36·8)	53·8% (45·9–62·9)	62·8% (53·8–72·7)	72·7% (62·1–83·6)	133·9%
Caucasus and Central Asia	54·9% (37·9–69·6)	67·6% (51·5–78·8)	71·8% (44·1–86·4)	76·0% (39·4–93·2)	38·4%
Latin America and the Caribbean	67·0% (56·2–73·4)	71·8% (66·5–76·1)	73·4% (68·6–77·0)	74·7% (67·3–80·1)	11·5%
Oceania	26·9% (7·7–61·5)	20·8% (12·0–33·0)	19·1% (14·1–25·1)	18·6% (11·2–33·5)	−30·8%

Details on the regional country distribution are provided in the appendix.

Time trends for the coverage of early antenatal care visits were also estimated for income groups according to the World Bank Group income groupings.^19^ These estimates suggest substantial inequality in coverage between income groups not only in 1990, but also persisting in 2013 despite steady progress being achieved in all four income groups (table 2).

**Table 2:** Global and regional trend estimates of coverage of early antenatal care visit by World Bank Group income groups*

	**1990**	**1995**	**2000**	**2005**	**2010**	**2013**
Low income	16·1% (12·6–22·4)	16·8% (14·1–21·3)	17·9% (16·1–20·7)	19·7% (18·3–21·3)	22·2% (20·4–24·1)	24·0% (21·7–26·5)
Lower middle income	26·3% (22·8–30·6)	31·5% (28·8–34·5)	37·1% (34·7–39·6)	43·0% (39·5–46·6)	48·8% (43·3–54·1)	52·1% (45·4–58·4)
Upper middle income	61·7% (52·8–68·9)	65·8% (59·4–71·2)	69·8% (65·6–73·5)	73·0% (69·8–75·8)	75·2% (71·1–78·5)	76·1% (70·9–80·1)
High income	77·2% (72·7–80·9)	79·0% (75·6–82·3)	80·4% (77·1–84·0)	81·3% (77·4–85·3)	81·8% (77·0–86·5)	81·9% (76·5–87·1)
World	40·9% (34·6–46·7)	44·6% (40·2–48·8)	48·6% (45·1–51·9)	52·6% (48·7–56·3)	56·4% (51·0–61·3)	58·6% (52·1–64·3)

Data are % (95% uncertainty interval).

* Details of the distribution of the income groups are provided in the appendix.

In 2013, overall coverage of early antenatal care visits in developing regions was 48·1% (95% UI 43·4–52·4) compared with 84·8% (81·6–87·7) in developed regions. The regional coverage of early antenatal care visits in 2013 ranged from 18·6% (95% UI 11·2–33·5) in Oceania to 96·5% (95·5–97·3) in Eastern Asia. The regions with the lowest early antenatal care visit coverage in 2013 were Oceania (18·6% [95% UI 11·2–33·5]), followed by sub-Saharan Africa (24·9% [22·6–27·2]); both regions are also the only regions with less than 50% coverage. The regions with an estimated coverage greater than 75% were Eastern Asia, developed regions, Caucasus and Central Asia, and South-eastern Asia.

In 2013, the estimated coverage of early antenatal care visits was 24·0% (95% UI 21·7–26·5) in low-income countries compared with 81·9% (76·5–97·1) in high-income countries. In 2013, the estimate for lower-middle-income countries was 52·1% (95% UI 45·4–58·4) and for upper-middle-income groups was 76·1% (70·9–80·1), which indicates substantial differences compared with high-income countries.

## Discussion

This study provides a comprehensive analysis of the global and regional coverage of early antenatal care visits based on data from 132 countries. The estimates suggest that the global coverage was 58·6% (95% UI 52·1–64·3) in 2013 with noteworthy regional variation. Although progress in coverage has been achieved, the level of coverage is much lower than required to meet one of the core recommendations of the WHO early antenatal care visit programme,^4^ which recommends that all pregnant women should initiate their first antenatal care visit in the first trimester of pregnancy to allow enough time for optimal care and treatment.

Evidence has suggested that many barriers and factors exist that contribute to the low uptake of the early antenatal care visit. The mother's level of education is one of the major determinants as well as socioeconomic status, but factors such as availability, accessibility, acceptability, family support, and previous experiences with the health system also affect the timing of the first visit.^22^ Despite a national and global focus on maternal and child health, first with the establishment of MDG 4 and MDG 5,^23^ which mainly focused on low-income and middle-income countries and to a much lesser extent on high-income countries, countries also vary greatly in implementation of global monitoring frameworks and antenatal care guidelines. With the new SDG agenda,^24^ which has set ambitious goals and targets for maternal and child health (leaving nobody behind), this is, to our knowledge, the first study that presents global and regional levels and trends of coverage of early antenatal care visits.

The study has highlighted differences in regional coverage. Two regions, sub-Saharan Africa and Oceania, had early antenatal care visit coverage of less than 50% in 2013. These are also the regions with the highest maternal mortality ratios (546 and 187 per 100 000 livebirths in 2015, respectively),^25^ neonatal mortality rates (29 and 22 per 1000 livebirths in 2015, respectively),^26^ and stillbirth rate in sub-Saharan Africa in 2015 (28·7 per 1000 total births),^27^ suggesting that early antenatal care visits are likely to be linked with health outcomes for women and children.

Regional findings can mask the variation between countries within the region, and regional estimates of early antenatal care visit coverage are likely to be driven by the countries with several data points and the number of livebirths in the individual countries. The levels and trends of coverage for the Eastern Asia region should be interpreted with caution because only data from South Korea informed the estimates for this region because of a lack of data for other countries in the region. The same is true for Oceania, for which we were only able to locate data from five countries: Papua New Guinea, Samoa, Solomon Islands, Tonga, and Vanuatu. No data for Fiji, the largest country in the region, were identified so the regional estimates are informed by limited observed data.

Equity is a prominent part of the 2030 Agenda for Sustainable Development, the vision of which is a world with equitable and universal access to quality health care. SDG target 17.18 calls for countries to increase the availability of disaggregated data by a range of determinants such as income, age, race, ethnicity, and geographic location.^8^ The estimates indicate that inequalities persist in coverage of the early antenatal care visit and the gap between low-income and high-income countries continues and has not changed in the past two decades. Additional disaggregation by other determinants was not possible with the current dataset.

The strengths of this study are the extensive and systematic approach applied to identify all potential national level data on coverage of the early antenatal care visit, and for the first time, comparable global and regional trend estimates have been developed. Nevertheless, the study had a number of shortcomings and limitations. Estimating coverage of the early antenatal care visit is challenging because of the paucity and the quality of data, which might be due to relatively limited attention being given to the early antenatal care visit compared with antenatal care coverage of at least one visit or of at least four visits, which were monitored and evaluated during the MDG era.^28^ Given that this study is at the ecological level, we were not able to analyse the association between women having an early antenatal care visit or at least four antenatal care visits, even though it would be very important to know if the women coming for an early antenatal care visit also use the antenatal care services later in pregnancy.

We have not accounted for change in World Bank Group income groups categories over the years as this information is not publicly available. Other estimation work reporting on the World Bank Group income groups also only applies to the most recent categories.^29^

Data quality is particularly affected by the uncertainty of the assessment of gestational age because of the woman's recall of last menstrual period and how the questions were asked in the household surveys. In the current DHS survey questionnaire^11^ it is only possible to report the gestational age in months because the question asked is “How many months pregnant were you when you first received antenatal care for this pregnancy?”. In the MICS round 5 surveys^12^ women also have the opportunity to report in weeks, which might allow for more accurate assessment of gestational age.

The ideal denominator for measuring the coverage of the early antenatal care visit is the number of pregnant women in a given time period, which also applies to other antenatal care indicators such as coverage of at least one antenatal care visit or at least four antenatal care visits. It is very difficult to calculate the number of pregnant women without doing prospective studies of large groups of women. Instead, we used the numbers of livebirths as the denominator because number of livebirths approximates the number of pregnancies. However, this measure excludes women who have stillbirths, induced and spontaneous abortions, and ectopic pregnancies, whereas women who have multiple livebirths (eg, twins or triplets) are counted multiple times in the denominator.^30^, ^31^ This uncertainty should be taken into consideration when interpreting the estimates, although currently, the number of livebirths remains the best available denominator. Despite widespread use of maternal health coverage indicators, many of these indicators have not been validated,^32^ and this also applies to early antenatal care visit coverage. Research is warranted to ensure valid and sensitive measurement of early antenatal care visit coverage that can inform programmatic and strategic decisions.

We applied statistical modelling to adjust for the different cutoffs used to report the early antenatal care visit and data limitations. Only countries with data were included in the analysis, which means that regional estimates were informed by few observations in regions with data from only a few countries and uncertainty intervals could be overly narrow due to limited variability in the sparse observations. When quantifying the uncertainty of the estimates, random sampling error was accounted for, but not model uncertainty and uncertainty associated with assessment of gestational age. Statistical modelling is a tool for generating comparable data and can provide valuable information to guide policy and programmes in settings with limited data but should not be used as a replacement for robust health information systems and empirical data.^33^

Our estimates of coverage of early antenatal care visits can provide important policy and programme planning guidance. However, to better inform national and local policies and programmes, targeted improvement in the measurement of coverage of early antenatal care visits is essential. Furthermore, research is needed to better understand the reasons why pregnant women might not initiate antenatal care in the first trimester of pregnancy and to address these determinants using innovative interventions that are adapted and appropriate to local settings. Coverage of early antenatal care visits only measured service contact, which is also the case for other coverage indicators such as coverage of at least one antenatal care visit or at least four antenatal care visits. Better methods are needed to produce more accurate data on intervention coverage, which should be complemented with assessments of service quality and interventions delivered during the visit.^13^

## References

[1] Graham W, Woodd S, Byass P (2016). Diversity and divergence: the dynamic burden of poor maternal health. Lancet.

[2] WHO (2015). Strategies toward ending preventable maternal mortality (EPMM).

[3] WHO (2015). Every newborn: an action plan to end preventable deaths.

[4] WHO (2016). WHO recommendations on antenatal care for a positive pregnancy experience.

[5] Souza JP, Tuncalp O, Vogel JP (2014). Obstetric transition: the pathway towards ending preventable maternal deaths. BJOG.

[6] EBCOG Scientific Committee (2015). The public health importance of antenatal care. Facts Views Vis Obgyn.

[7] Zolotor AJ, Carlough MC (2014). Update on prenatal care. Am Fam Physician.

[8] UN (2015). Sustainable development goals.

[9] Every Woman Every Child (2015). Global strategy for women's, children's and adolescents' health 2016–2030.

[10] World Bank (2016). Global financing facility in support of Every Woman Every Child (GFF).

[11] USAID ICF International. The DHS Program—Demographic and Health Surveys. http://dhsprogram.com/.

[12] UNICEF (2006). Multiple indicator cluster surveys. http://mics.unicef.org/.

[13] Bryce J, Arnold F, Blanc A (2013). Measuring coverage in MNCH: new findings, new strategies, and recommendations for action. PLoS Med.

[14] Stevens GA, Alkema L, Black RE (2016). Guidelines for accurate and transparent health estimates reporting: the GATHER statement. PLoS Med.

[15] Division of Reproductive Health (2006). Reproductive Health Surveys.

[16] Euro-Peristat Network Euro-Peristat. http://www.europeristat.com/.

[17] Chen H, Yu P, Hailey D, Wang N (2014). Methods for assessing the quality of data in public health information systems: a critical review. Stud Health Technol Inform.

[18] UN (2014). Official Millennium Development Goal (MDG) regional groupings and their corresponding countries.

[19] World Bank Group (2016). Country and lending groups [internet].

[20] WHO (2010). International statistical classification of diseases and related health problems, tenth revision. Volume 2: instruction manual.

[21] UN (2015). World population prospects: the 2015 revision.

[22] Gill K, Pande R, Malhotra A (2007). Women deliver for development. Lancet.

[23] UN (2000). United Nations millennium declaration. Resolution adopted by the General Assembly, Sept 18, 2000. A/RES/55/2.

[24] UN (2015). Transforming our world: the 2030 agenda for sustainable development. Resolution adopted by the General Assembly, Sept 25, 2015. A/RES/70/1.

[25] Alkema L, Chou D, Hogan D (2016). Global, regional, and national levels and trends in maternal mortality between 1990 and 2015, with scenario-based projections to 2030: a systematic analysis by the UN Maternal Mortality Estimation Inter-Agency Group. Lancet.

[26] You D, Hug L, Ejdemyr S (2015). Global, regional, and national levels and trends in under-5 mortality between 1990 and 2015, with scenario-based projections to 2030: a systematic analysis by the UN Inter-agency Group for Child Mortality Estimation. Lancet.

[27] Blencowe H, Cousens S, Jassir FB (2016). National, regional, and worldwide estimates of stillbirth rates in 2015, with trends from 2000: a systematic analysis. Lancet Glob Health.

[28] UN (2008). Millennium Development Goals. Official list of MDG indicators.

[29] WHO, UNICEF, UNFPA, World Bank Group and the United Nations Population Division (2015). Trends in maternal mortality: 1990 to 2015. Estimates by WHO, UNICEF, UNFPA, World Bank Group and the United Nations Population Division.

[30] Blencowe H, Calvert C, Lawn JE, Cousens S, Campbell OMR (2016). Measuring maternal, fetal and neonatal mortality: challenges and solutions. Best Pract Res Clin Obstet Gynaecol.

[31] WHO (2011). Unsafe abortion: global and regional estimates of the incidence of unsafe abortion and associated mortality in 2008.

[32] McCarthy KJ, Blanc AK, Warren CE, Kimani J, Mdawida B, Ndwidga C (2016). Can surveys of women accurately track indicators of maternal and newborn care? A validity and reliability study in Kenya. J Glob Health.

[33] Boerma T, Mathers CD (2015). The World Health Organization and global health estimates: improving collaboration and capacity. BMC Med.

