# Supplementary material for: Early antenatal care visit: a systematic analysis of regional and global levels and trends of coverage from 1990 to 2013
Source: Lancet Glob Health. 2017 Sep 11;5(10):e977–83. doi: 10.1016/S2214-109X(17)30325-X (PMC5603717; doi:10.1016/S2214-109X(17)30325-X)
Supplement: Supplementary appendix [file mmc1.pdf]

THE LANCET
Global Health

Supplementary appendix

This appendix formed part of the original submission and has been peer reviewed. We post it as supplied by the authors.

Supplement to: Moller A-B, Petzold M, Chou D, Say L. Early antenatal care visit: a systematic analysis of regional and global levels and trends of coverage from 1990 to 2013. *Lancet Glob Health* 2017; **5:** e977–83.

Early antenatal care visit:
a systematic analysis of regional and global levels and trends of coverage from 1990 to 2013

Supplementary web appendix

Ann-Beth Moller, Max Petzold, Doris Chou and Lale Say

Contents

Appendix 1. Guidelines for Accurate and Transparent Health Estimates Reporting (GATHER) 3

Appendix 2. Literature review of available national level data on coverage of early ANC visit 5

Appendix 3. Millennium Development Goal (MDG) regional groupings 8

Appendix 4. World Bank Group income groups... 10

Appendix 5. Bayesian model 12

Appendix 6. Sources of early ANC visit coverage data used in the analysis by main source category, number of countries, and number of data points.. 13

Appendix 7. Input dataset (national level data) 14

Appendix 1. Guidelines for Accurate and Transparent Health Estimates Reporting (GATHER)[1]

Item no.	Checklist item	Location in submitted manuscript
**Objectives and funding**		
1	Define the indicator(s), populations (including age, sex, and geographic entities), and time period(s) for which estimates were made.	Included in the introduction
2	List the funding sources for the work.	Included under funding
**Data Inputs**		
For all data inputs from multiple sources that are synthesized as part of the study:		
3	Describe how the data were identified and how the data were accessed.	Included in the method section
4	Specify the inclusion and exclusion criteria. Identify all ad-hoc exclusions.	Included in the method section
5	Provide information on all included data sources and their main characteristics. For each data source used, report reference information or contact name/institution, population represented, data collection method, year(s) of data collection, sex and age range, diagnostic criteria or measurement method, and sample size, as relevant.	Supplementary Appendix 7 pp. 14-72
6	Identify and describe any categories of input data that have potentially important biases (e.g., based on characteristics listed in item 5).	Included in the method section
For data inputs that contribute to the analysis but were not synthesized as part of the study:		
7	Describe and give sources for any other data inputs.	Included in the method section
For all data inputs:		
8	Provide all data inputs in a file format from which data can be efficiently extracted (e.g., a spreadsheet as opposed to a PDF), including all relevant meta-data listed in item 5. For any data inputs that cannot be shared due to ethical or legal reasons, such as third-party ownership, provide a contact name or the name of the institution that retains the right to the data.	The dataset will be available at: www.who.int/reproductivehealth/early-anc-estimates/en/
**Data analysis**		
9	Provide a conceptual overview of the data analysis method. A diagram may be helpful.	Included in the results section
10	Provide a detailed description of all steps of the analysis, including mathematical formulae. This description should cover, as relevant, data cleaning, data pre-processing, data adjustments and weighting of data sources, and mathematical or statistical model(s).	Included in the method section

[1] Stevens GA, Alkema L, Black RE, Boerma JT, Collins GS, Ezzati M, et al. Guidelines for Accurate and Transparent Health Estimates Reporting: the GATHER statement. PLoS Med. 2016;13(6):e1002056

11	Describe how candidate models were evaluated and how the final model(s) were selected.	Appendix 5. p. 12

Appendix 2. Literature review of available national level data on coverage of early ANC visit

A literature review was conducted to search for national level data on early ANC visit coverage that poses to answer the following question:

What is the proportion of pregnant women who initiate their first ANC visit in the first trimester (early ANC visit) at national level in all WHO Members States. ? Data from 1990 to latest available will be considered.

The question was assessed based on the PIODS framework criteria: population, intervention, outcome, design and setting.

PIODS	Inclusion Criteria
Population	Women with a live birth
Intervention	Antenatal care
Outcome	Early antenatal care visit
Design	Population-based surveys, special perinatal studies, health information system reports (administrative data)
Setting	All WHO Member States (http://www.who.int/countries/en/)

National level data on early ANC visit were obtained from sources which can be divided into two main categories:

1. Routine management health information system reports from Ministries of Health (MoHs) an official publication of a Ministry of Health or National Statistical Offices (NSOs) an official publication of a National Statistical Office; and

2. Population-based household surveys such as; the Demographic and Health Surveys (DHS)[2] the Multiple Indicator Cluster Surveys (only MICS round 5)[3], the Reproductive Heaths Surveys (RHS)[4], other national surveys (e.g. the Family Health Surveys), and special national perinatal studies[5]

Search Strategy

The searches were conducted from 7th to 31st March 2016.

All Ministries of Health and National Statistical Offices websites were systematically searched for all the 194 WHO Members States without any language restrictions.

Only surveys and health information system reports published from 1990 to most recent were considered as data before 1990 are sparse, and 1990 has been the standard cut-off year for reporting global health estimates in the MDG era.[6]

MoH websites were identified using Google search applying the search terms '[country name] ministry of health'.

[2] USAID. ICF International. The DHS Program-Demographic and Health Surveys [Available from: http://dhsprogram.com/].
[3] UNICEF. Multiple Indicator Cluster Surveys [Available from: http://mics.unicef.org/].
[4] Division of Reproductive Health. Reproductive Health Surveys Atlanta, USA [Available from: http://www.cdc.gov/reproductivehealth/global/tools/surveys.htm].
[5] EuroPeristat [Available from: http://www.europeristat.com/].
[6] The Millennium Development Goals Report 2015. New York (N.Y.); 2015.

To identify data on early ANC visit coverage on the MoH websites the pages were searched utilizing the search terms:

- annual MoH reports, annual MoH statistics reports, perinatal and reproductive health statistics, and

- using the website search functions for terms such as "antenatal care", "prenatal", "reproductive health", "perinatal", "women's health", "mothers and babies".

- NSOs were identified using the United Nations website "Information on National Statistical Systems" which provides links to all NSOs.[7] Same search approach as described above for searching MoH websites were applied to the search on the NSO websites.

Population based household surveys such as the DHS were obtained online from the respective websites: IFC International, the MICS round 5 surveys from (United Nations Children's Fund (UNICEF), the RHS from Center for Chronic Disease Prevention and Health Promotion (CDC).

In addition, searches were conducted using Google and the Global Index Medicus (GIM) a search engine encompassing the 5 Regional WHO databases; AIM (AFRO), IMEMR (EMRO), LILACS (PAHO) Latin American & Caribbean Health Sciences, IMSEAR (SEARO), and WPRIM (WPRO) for RHS surveys as not all available RHS surveys are included on the CDC website as well as some national published DHS (e.g. Turkey and Timor Leste).

Perinatal surveys were obtained from the EuroPeristat webpage and to identify perinatal studies outside of Europe we did a Google search using the search terms; perinatal study, perinatal survey, maternity, maternity care.

Inclusion and exclusion criteria

Inclusion criteria:

- Population based health surveys, health information system reports, and perinatal studies which reported national level data from 1990-to latest available on coverage of early ANC visit.
- Information in relation to which weeks/month(s) the pregnant women had their first ANC visit were specified.
- Specification of data coverage period (also referred to as re-call period) and data collection period specified.

Exclusion criteria:

- Sub-national data (i.e. 1st and 2nd administrative level of a country e.g. district or department).

Quality assessment of data sources

No well-established tool for standardized appraisal of public health information systems have been developed and applied. A recent systematic review by Chen H et al., 2014 looked at "methods for assessing the quality of data in public health information systems" and concluded: "…. there are limitations of current data quality assessment methods: a lack of consensus on attributes measured; inconsistent definition of the data quality attributes; a lack of mixed methods for assessing data quality; and inadequate attention to reliability and validity".[8]

For the developing countries data were mainly retrieved from the DHS programme and the MICS programme. The DHS programme implemented by IFC International started in 1984 and has received worldwide reputation for

[7] https://unstats.un.org/home/nso_sites/.

[8] Chen H, Yu P, Hailey D, Wang N. Methods for assessing the quality of data in public health information systems: a critical review. Studies in health technology and informatics. 2014;204:13-8.

collecting and disseminating nationally representative data on maternal and child health in more than 300 surveys in over 90 countries. The MICS programme implemented by UNICEF has conducted surveys since 1995 mainly in countries not covered under the IFC International program and is an important source of comparable data and more than 280 surveys have been implemented in 100 low- and middle-income countries but only in the last round of surveys (round 5) the data on early ANC visit have been collected. In both programmes, surveys are conducted by trained personnel using standardized questionnaires and methods for data collection and processing, and usually implemented by the National Statistical Office. These surveys are considered the best available way of obtaining several types of maternal health indicators in developing countries[9].

The Authority, Accuracy, Coverage, Objectivity, Date, Significance (AACODS) checklist was used to assess the quality of the data sources. It assesses six domains; authority, accuracy, coverage, objectivity, date, and significance. Each domain has sub questions to further appraise each of the domains.[10] Only sources which fulfilled the requirement suggested in the AACODS checklist were included.

[9] Bryce J, Arnold F, Blanc A, Hancioglu A, Newby H, Requejo J, et al. Measuring Coverage in MNCH: New Findings, New Strategies, and Recommendations for Action. PLoS Med. 2013;10(5):e1001423.
[10] Tyndall J. The Authority, Accuracy, Coverage, Objectivity, Date, Significance (AACODS) Checklist: Flinders University; 2010 [Available from: http://dspace.flinders.edu.au/dspace/].

Appendix 3. Millennium Development Goal (MDG) regional groupings

Developed regions
Developing regions:

Caucasus and Central Asia
Eastern Asia
Latin America and the Caribbean
Northern Africa
Oceania
South-eastern Asia
Southern Asia
Sub-Saharan Africa
Western Asia

Countries in each grouping (183 countries included in the analysis)[11]

Region	Countries
Caucasus and Central Asia	Armenia, Azerbaijan, Georgia, Iran (Islamic Republic of), Kazakhstan, Kyrgyzstan, Tajikistan, Turkmenistan, Uzbekistan
Developed region	Argentina, Australia, Austria, Belgium, Bulgaria, Belarus, Canada, Switzerland, Chile, Cyprus, Czechia, Germany, Denmark, Spain, Estonia, Finland, France, Greece, Hungary, Ireland, Iceland, Israel, Italy, Japan, Republic of Korea, Lithuania, Luxembourg, Latvia, Republic of Moldova, Malta, Netherlands, Norway, New Zealand, Poland, Portugal, Romania, Russian Federation, Singapore, Slovakia, Slovenia, Sweden, Ukraine, United Kingdom, United States of America
Eastern Asia	China, Mongolia, Democratic People's Republic of Korea
Latin America and the Caribbean	Antigua and Barbuda, Argentina, Bahamas, Barbados, Belize, Bolivia, Brazil, Chile. Colombia, Costa Rica, Cuba, Dominican Republic, Ecuador, El Salvador, Grenada, Guatemala, Guyana, Haiti, Honduras, Jamaica, Mexico, Nicaragua, Panama, Paraguay, Peru, Saint Lucia, Saint Vincent and the Grenadines, Suriname, Trinidad and Tobago, Uruguay, Venezuela (Bolivarian Republic of)
Northern Africa	Algeria, Egypt, Libya, Morocco, Tunisia
Oceania	Cook Islands, Fiji, Micronesia (Federated States of), Kiribati, Marshall Islands, Papua New Guinea, Samoa, Solomon Islands, Tonga, Vanuatu
South-eastern Asia	Brunei Darussalam, Cambodia, Indonesia, Lao People's Democratic Republic Malaysia, Myanmar, Philippines, Singapore, Thailand, Timor-Leste, Viet Nam
Southern Asia	Afghanistan, Bangladesh, Bhutan, India, Iran (Islamic Republic of), Maldives, Nepal, Pakistan, Sri Lanka
Sub-Saharan Africa	Angola, Benin, Botswana, Burkina Faso, Burundi, Cabo Verde, Cameroon, Central African Republic, Chad, Comoros, Congo, Cote d'Ivoire, Democratic Republic of the Congo, Djibouti, Equatorial Guinea, Eritrea, Ethiopia, Gabon, Gambia, Ghana, Guinea, Guinea-Bissau, Kenya, Lesotho, Liberia, Madagascar, Malawi, Mali, Mauritania, Mauritius, Mozambique, Namibia, Niger, Nigeria, Rwanda, Sao Tome and Principe, Senegal, Seychelles, Sierra Leone, Somalia, South Africa, Sudan, Swaziland, Togo ,Uganda, United Republic of Tanzania, Zambia, Zimbabwe
Western Asia	Bahrain, Iraq, Jordan, Kuwait, Lebanon, Oman, Qatar, Saudi Arabia, Syrian Arab Republic, Turkey, United Arab Emirates, Yemen

[11] Official Millennium Development Goal (MDG) Regional Groupings and their corresponding countries [Internet]. New York (N.Y); 2014. Available from: http://mdgs.un.org/unsd/mdg/Host.aspx?Content=Data/RegionalGroupings.htm.

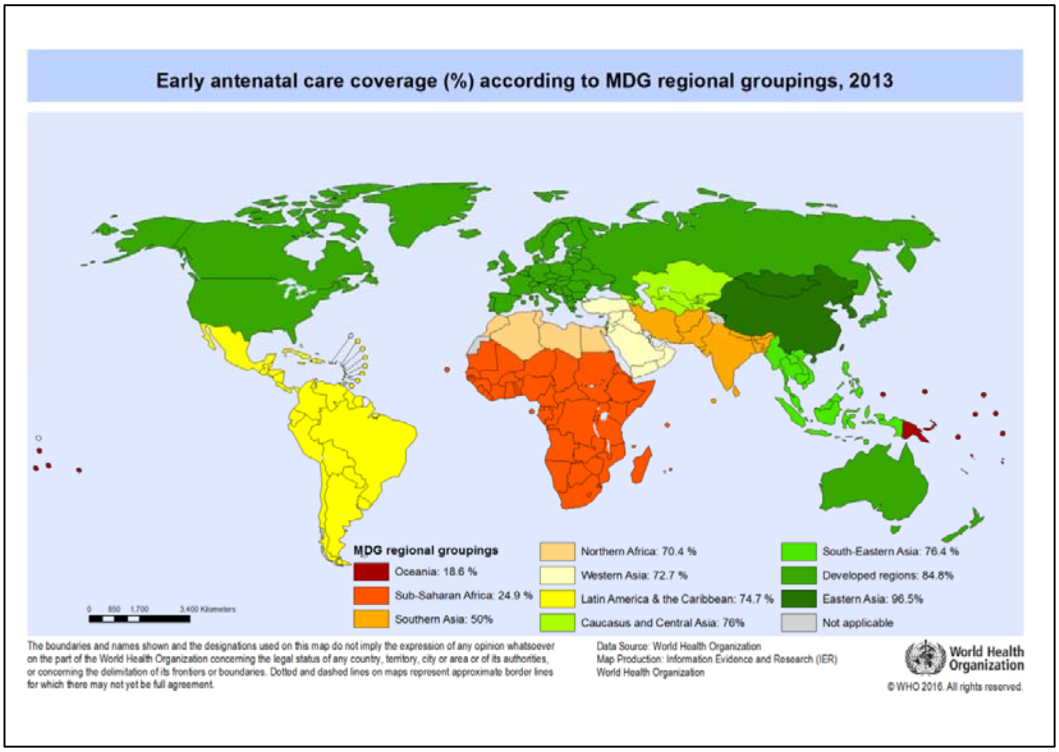

Early antenatal care coverage (%) according to MDG regional groupings, 2013
MDG regional groupings
Oceania: 18.6 %
Sub-Saharan Africa: 24.9 %
Southern Asia: 50%
Northern Africa: 70.4 %
Western Asia: 72.7 %
Latin America & the Caribbean: 74.7 %
Caucasus and Central Asia: 76%
South-Eastern Asia: 76.4 %
Developed regions: 84.8%
Eastern Asia: 96.5%
Not applicable
0 850 1,700 3,400 Kilometers
The boundaries and names shown and the designations used on this map do not imply the expression of any opinion whatsoever on the part of the World Health Organization concerning the legal status of any country, territory, city or area or of its authorities, or concerning the delimitation of its frontiers or boundaries. Dotted and dashed lines on maps represent approximate border lines for which there may not yet be full agreement.
Data Source: World Health Organization
Map Production: Information Evidence and Research (IER)
World Health Organization
World Health Organization
© WHO 2016. All rights reserved.

Appendix 4. World Bank Group income groups

For the current 2016 fiscal year, low-income economies are defined as those with a gross national income (GNI) per capita, calculated using the World Bank Atlas method, of $1,045 or less in 2014; middle-income economies are those with a GNI per capita of more than $1,045 but less than $12,736; high-income economies are those with a GNI per capita of $12,736 or more.[12]

Low income ($1,045 or less)	Lower middle income ($1,046 to $4,125)	Upper middle income ($4,126 to $12,735)	High income ($12,736 or more)
Afghanistan	Armenia	Albania	Antigua and Barbuda
Benin	Bangladesh	Algeria	Argentina
Burkina Faso	Bhutan	Angola	Australia
Burundi	Bolivia (Plurinational State of)	Azerbaijan	Austria
Cambodia	Cabo Verde	Belarus	Bahamas
Central African Republic	Cameroon	Belize	Bahrain
Chad	Congo	Bosnia and Herzegovina	Barbados
Comoros	Côte d'Ivoire	Botswana	Belgium
Democratic People's Republic of Korea	Djibouti	Brazil	Brunei Darussalam
Democratic Republic of the Congo	Egypt	Bulgaria	Canada
Eritrea	El Salvador	China	Chile
Ethiopia	Georgia	Colombia	Croatia
Gambia	Ghana	Costa Rica	Cyprus
Guinea	Guatemala	Cuba	Czechia
Guinea-Bissau	Guyana	Dominican Republic	Denmark
Haiti	Honduras	Ecuador	Equatorial Guinea
Liberia	India	Fiji	Estonia
Madagascar	Indonesia	Gabon	Finland
Malawi	Kenya	Grenada	France
Mali	Kiribati	Iran (Islamic Republic of)	Germany
Mozambique	Kyrgyzstan	Iraq	Greece
Nepal	Lao People's Democratic Republic	Jamaica	Hungary
Niger	Lesotho	Jordan	Iceland
Rwanda	Mauritania	Kazakhstan	Ireland
Sierra Leone	Micronesia (Federated States of)	Lebanon	Israel
Somalia	Morocco	Libya	Italy
South Sudan	Myanmar	Malaysia	Japan
Togo	Nicaragua	Maldives	Kuwait
Uganda	Nigeria	Mauritius	Latvia
United Republic of Tanzania	Pakistan	Mexico	Lithuania
Zimbabwe	Papua New Guinea	Mongolia	Luxembourg
	Philippines	Montenegro	Malta
	Republic of Moldova	Namibia	Netherlands
	Samoa	Panama	New Zealand
	Sao Tome and Principe	Paraguay	Norway

[12] World Bank Group. Country and Lending Groups [Internet]. Washington D.C.: World Bank Group; 2016 [cited 02 January 2017]. Available from: http://data.worldbank.org/about/country-and-lending-groups#High_income.

Low income ($1,045 or less)	Lower middle income ($1,046 to $4,125)	Upper middle income ($4,126 to $12,735)	High income ($12,736 or more)
	Senegal	Peru	Oman
	Solomon Islands	Romania	Poland
	Sri Lanka	Saint Lucia	Portugal
	Sudan	Saint Vincent and the Grenadines	Qatar
	Swaziland	Serbia	Republic of Korea
	Syrian Arab Republic	South Africa	Russian Federation
	Tajikistan	Suriname	Saudi Arabia
	Timor-Leste	Thailand	Seychelles
	Ukraine	The former Yugoslav Republic of Macedonia	Singapore
	Uzbekistan	Tonga	Slovakia
	Vanuatu	Tunisia	Slovenia
	Viet Nam	Turkey	Spain
	Yemen	Turkmenistan	Sweden
	Zambia		Switzerland
			Trinidad and Tobago
			United Arab Emirates
			United Kingdom
			United States of America
			Uruguay
			Venezuela (Bolivarian Republic of)

Appendix 5. Bayesian model

```
bayesmh national_perc_early i.region i.country_num i.country_num#c.midyear,            ///
   noconstant mcmcsize(10000) burnin(2500) likelihood(normal({var_res}))                ///
   prior({national_perc_early:i.region}, normal({national_perc_early:_cons}, {var_reg})) ///
   prior({national_perc_early:i.country_num i.country_num#c.midyear},                   ///
      mvnormal(2, 0, 0, {Covar, matrix}))                                                ///
   prior({national_perc_early:_cons}, normal(0, 100))                                   ///
   prior({var_res},   igamma(0.01, 0.01))                                                ///
                      prior({var_reg},   igamma(0.01, 0.01))                             ///
   prior({Covar,matrix},  iwishart(2, 3, I(2)))                                          ///
   block({national_perc_early:i.region}, reffects)                                      ///
   block({national_perc_early:i.country_num}, reffects)                                 ///
   block({national_perc_early:i.country_num#c.midyear}, reffects)                       ///
   saving(bayes_parameter_estimates_country_tmp, replace)
```

Model selection and evaluation:

We graphed the observed and predicted values per country and assessed the performance of the model visually. The basic linear model for logit transformed values was found to fit the data well. Using non-logit transformed data often lead to estimates less 0% and above 100% for the predicted values and UI. Given the fit it was decided to not include further covariates.

Appendix 6. Sources of early ANC visit coverage data used in the analysis by main source category, number of countries, and number of data points

Source of early ANC data and number of countries[a]	Countries	Number of data points
Population based household surveys (107 countries)	Afghanistan, Albania, Armenia, Azerbaijan, Bangladesh, Belize, Benin, Bhutan, Bolivia (Plurinational State of), Botswana, *Brazil*, Burkina Faso, Burundi, Cabo Verde, Cambodia, Cameroon, Chad, Colombia, Comoros, Congo, Costa Rica, Côte d'Ivoire, Cuba, *Czech Republic*, Democratic Republic of the Congo, Djibouti, Dominican Republic, Ecuador, Egypt, El Salvador, Equatorial Guinea, Eritrea, Ethiopia, Gabon, Gambia, Georgia, Ghana, Guatemala, Guinea, Guyana, Haiti, Honduras, India, Indonesia, Jamaica, Jordan, Kenya, Kiribati, Kyrgyzstan, Lao People's Democratic Republic, Lesotho, Liberia, Madagascar, Malawi, Maldives, Mali, Marshall Islands, Mauritania, *Mongolia*, Morocco, Mozambique, Myanmar, Namibia, Nauru, Nepal, Nicaragua, Niger, Nigeria, Pakistan, Panama, Paraguay, Peru, Philippines, Republic of Korea, Republic of Moldova, *Romania*, Russian Federation, Rwanda, Samoa, Sao Tome and Principe, Senegal, Serbia, Sierra Leone, Solomon Islands, South Africa, Sri Lanka, Sudan, Swaziland, Syrian Arab Republic, Thailand, Timor-Leste, Togo, Tonga, Tunisia, Turkey, Turkmenistan, Tuvalu, Uganda, Ukraine, United Republic of Tanzania, Uzbekistan, Vanuatu, Venezuela (Bolivarian Republic of), Viet Nam, Yemen, Zambia, Zimbabwe	324
Perinatal studies (5 countries)	*Brazil*, Canada, France, Luxembourg, *United Kingdom*	17
Administrative data (25 countries)	Australia, Croatia, Cyprus, *Czech Republic*, Estonia, Finland, Germany, Ireland, Italy, Latvia, Lithuania, Malta, Mauritius, *Mongolia*, Netherlands, New Zealand, Oman, Palau, Portugal, *Romania*, Saudi Arabia, Slovakia, Slovenia, *United Kingdom*, United States of America	175
No national data (62 countries)	Algeria, Andorra, Angola, Antigua and Barbuda, Argentina, Austria, Bahamas, Bahrain, Barbados, Belarus, Belgium, Bosnia and Herzegovina, Brunei Darussalam, Bulgaria, Central African Republic, Chile, China, Cook Islands, Democratic People's Republic of Korea, Denmark, Dominica, Fiji, Greece, Grenada, Guinea-Bissau, Hungary, Iceland, Iran (Islamic Republic of,) Iraq, Israel, Japan, Kazakhstan, Kuwait, Lebanon, Libya, Malaysia, Mexico, Micronesia (Federated States of), Monaco, Montenegro, Niue, Norway, Papua New Guinea, Poland, Qatar, Saint Kitts and Nevis, Saint Lucia, Saint Vincent and the Grenadines, San Marino, Seychelles, Singapore, Somalia, South Sudan, Spain, Suriname, Sweden, Switzerland, Tajikistan, The former Yugoslav Republic of Macedonia, Trinidad and Tobago, United Arab Emirates, Uruguay	0

a. Five countries had more than one type of data source: Brazil, Czech Republic, Mongolia, Romania, and United Kingdom (marked in italic).

Appendix 7. Input dataset (national level data)

ISO3	Country name	Data coverage period start year	Data coverage period end year	Coverage of early ANC visits (%) (women 15-49 years)	No. of pregnant women (15-49 years) having early ANC visit	Timing of first visit codes: 1=<4 mo; 2=<14 weeks; 3=<15 weeks; 4=<16 weeks; 5=1st trimester; 6=<6 mo; 7<12 weeks; 8<13 weeks; 9<20 weeks; 10<3 mo	Sample size	Source code: 1=survey; 2=adm.; 3= perinatal	Sources
AFG	Afghanistan	2005	2010	18.2		1	16998	1	Afghan Public Health Institute, Ministry of Public Health (APHI/MoPH) [Afghanistan], Central Statistics Organization (CSO) [Afghanistan], ICF Macro, Indian Institute of Health Management Research (IIHMR) [India], and World Health Organization Regional Office for the Eastern Mediterranean (WHO/EMRO) [Egypt]. Afghanistan Mortality Survey 2010. Calverton, Maryland, USA: APHI/MoPH, CSO, ICF Macro, IIHMR and WHO/EMRO; 2011.
ALB	Albania	1997	2002	59.3		4	2551	1	Institute of Public Health, Ministry of Health, Institute of Statistics, DRH/CDC. Reproductive Health Survey Albania, 2002. Final Report. Atlanta, Georgia, USA: DHR/CDC; 2005.
ALB	Albania	2003	2009	77.8		1	1310	1	Institute of Statistics, Institute of Public Health [Albania] and ICF Macro. Albania Demographic and Health Survey 2008-09. Tirana, Albania: Institute of Statistics, Institute of Public Health and ICF Macro; 2010.
ARM	Armenia	1995	2000	53.1		1	1248	1	National Statistical Service [Armenia], Ministry of Health [Armenia], and ORC Macro. Armenia Demographic and Health Survey 2000. Calverton, Maryland: National Statistical Service, Ministry of Health, and ORC Macro; 2001.
ARM	Armenia	2000	2005	48.2		1	1097	1	National Statistical Service [Armenia], Ministry of Health [Armenia], and ORC Macro. Armenia Demographic and Health Survey 2005. Calverton, Maryland: National Statistical Service, Ministry of Health, and ORC Macro; 2006.
ARM	Armenia	2005	2010	79.7		1	1140	1	National Statistical Service [Armenia], Ministry of Health [Armenia], and ICF International. Armenia Demographic and Health Survey 2010. Calverton, Maryland: National Statistical Service, Ministry of Health, and ICF International; 2012.

ISO3	Country name	Data coverage period start year	Data coverage period end year	Coverage of early ANC visits (%) (women 15-49 years)	No. of pregnant women (15-49 years) having early ANC visit	Timing of first visit codes: 1=<4 mo; 2=<14 weeks; 3=<15 weeks; 4=<16 weeks; 5=1st trimester; 6=<6 mo; 7<12 weeks; 8<13 weeks; 9<20 weeks; 10<3 mo	Sample size	Source code: 1=survey; 2=adm.; 3= perinatal	Sources
AUS	Australia	2012	2012	62.7	186897	2	307474	2	Hilder L, Zhichao Z, Parker M, Jahan S, Chambers GM. Australia's mothers and babies 2012. Perinatal statistics series no. 30. Cat. no. PER 69. Canberra: AIHW; 2014.
AUS	Australia	2011	2011	65.7	188040	2	297126	2	Li Z, Zeki R, Hilder L & Sullivan EA. Australia's mothers and babies 2011. Perinatal statistics series no. 28. Cat. no. PER 59. Canberra: AIHW National Perinatal Epidemiology and Statistics Unit; 2013.
AUS	Australia	2010	2010	65	189740	2	291840	2	Li Z, Zeki R, Hilder L and Sullivan EA. Australia's mothers and babies 2010. Perinatal statistics series no. 27. Cat. no. PER 57. Canberra: AIHW National Perinatal Epidemiology and Statistics Unit; 2012.
AZE	Azerbaijan	1996	2001	44.8		5	3430	1	Ministry of Health, DRH/CDC. Reproductive Health Survey Azerbaijan, 2001. Final Report. Atlanta, Georgia, USA: CDC; 2003.
AZE	Azerbaijan	2001	2006	53.8		1	1686	1	State Statistical Committee (SSC) [Azerbaijan] and Macro International Inc. Azerbaijan Demographic and Health Survey 2006. Calverton, Maryland, USA: State Statistical Committee and Macro International Inc.; 2008.
AZE	Azerbaijan	2006	2011	77.5		1	1623	1	Public Health and Reforms Center (PHRC). Demographic and Health Survey 2011 Ministry of Health, Baku, Azerbaijan; 2013.
BGD	Bangladesh	1990	1994	10.9		1	3850	1	Mitra SN, Nawab Ali M, Shahidul Islam, Cross AR, Saha T. Bangladesh Demographic and Health Survey, 1993-1994. Calverton, Maryland: National Institute of Population Research and Training (NIPORT), Mitra and Associates, and Macro International Inc.; 1994.
BGD	Bangladesh	1991	1997	13.7		1	6230	1	Mitra SN, Ahmed Al-Sabir, Cross AR, Kanta Jamil K. Bangladesh Demographic and Health Survey, 1996-1997. Dhaka and Calverton, Maryland: National Institute of Population Research and Training (NIPORT), Mitra and Associates, and Macro International Inc.; 1997.
BGD	Bangladesh	1994	2000	12		1	5263	1	National Institute of Population Research and Training (NIPORT), Mitra and Associates (MA), and ORC Macro (ORCM). Bangladesh Demographic and

ISO3	Country name	Data coverage period start year	Data coverage period end year	Coverage of early ANC visits (%) (women 15-49 years)	No. of pregnant women (15-49 years) having early ANC visit	Timing of first visit codes: 1=<4 mo; 2=<14 weeks; 3=<15 weeks; 4=<16 weeks; 5=1st trimester; 6=<6 mo; 7<12 weeks; 8<13 weeks; 9<20 weeks; 10<3 mo	Sample size	Source code: 1=survey; 2=adm.; 3= perinatal	Sources
									Health Survey 1999-2000. Dhaka, Bangladesh and Calverton, Maryland [USA]: National Institute of Population Research and Training, Mitra and Associates, and ORC Macro; 2001.
BGD	Bangladesh	1998	2001	14.6		1	40657	1	National Institute of Population Research and Training (NIPORT), ORC Macro, Johns Hopkins University and ICDDR,B. Bangladesh Maternal Health Services and Maternal Mortality Survey 2001. Dhaka, Bangladesh and Calverton, Maryland, USA: NIPORT, ORC Macro, Johns Hopkins University, and ICDDR,B; 2003.
BGD	Bangladesh	1999	2004	19.5		1	5416	1	National Institute of Population Research and Training (NIPORT), Mitra and Associates, and ORC Macro. Bangladesh Demographic and Health Survey 2004. Dhaka, Bangladesh and Calverton, Maryland [USA]: National Institute of Population Research and Training, Mitra and Associates, and ORC Macro; 2005.
BGD	Bangladesh	2002	2007	24.4		1	4905	1	National Institute of Population Research and Training (NIPORT), Mitra and Associates, and Macro International. Bangladesh Demographic and Health Survey 2007. Dhaka, Bangladesh and Calverton, Maryland, USA: National Institute of Population Research and Training, Mitra and Associates, and Macro International; 2009.
BGD	Bangladesh	2007	2010	21.3		1	17149	1	National Institute of Population Research and Training (NIPORT), MEASURE Evaluation, and icddr,b. Bangladesh Maternal Mortality and Health Care Survey 2010. Dhaka, Bangladesh: NIPORT, MEASURE Evaluation, and icddr,b; 2012.
BLZ	Belize	1986	1991	42.8		1	1910	1	Central Statistical Office, Ministry of Finance, CDC. 1991 Belize Family Health Survey. Final Report. Atlanta, Georgia, USA: CDC; 1992.
BLZ	Belize	1991	1999	47.8		1	2094	1	Central Statistical Office. 1999 Belize Family Health Survey - Females. Belize; 2001.
BEN	Benin	1993	1996	23.8		1	2939	1	Nicalse K, Mboup G, Tossou J, I'Aopoldine de Souza, Gandaho T et al. Enquête Démographique et de Santé, République de Bénin 1996. Calverton,

ISO3	Country name	Data coverage period start year	Data coverage period end year	Coverage of early ANC visits (%) (women 15-49 years)	No. of pregnant women (15-49 years) having early ANC visit	Timing of first visit codes: 1=<4 mo; 2=<14 weeks; 3=<15 weeks; 4=<16 weeks; 5=1st trimester; 6=<6 mo; 7<12 weeks; 8<13 weeks; 9<20 weeks; 10<3 mo	Sample size	Source code: 1=survey; 2=adm.; 3= perinatal	Sources
									Maryland USA: Institut National de la Statistique et de l'Analyse Économique et Macro International Inc.; 1997.
BEN	Benin	1996	2001	36.8		1	3524	1	Institut National de la Statistique et de l'Analyse Économique (INSAE) et ORC Macro. Enquête Démographique et de Santé au Bénin 2001. Calverton, Maryland, USA: Institut National de la Statistique et de l'Analyse Économique et ORC Macro; 2002.
BEN	Benin	2001	2006	41.7		1	10521	1	Institut National de la Statistique et de l'Analyse Économique (INSAE) [Bénin] et Macro International Inc. Enquête Démographique et de Santé (EDSB-III) - Bénin 2006. Calverton, Maryland, USA : Institut National de la Statistique et de l'Analyse Économique et Macro International Inc.; 2007.
BEN	Benin	2006	2012	48		1	8993	1	Institut National de la Statistique et de l'Analyse Économique (INSAE) et ICF International. Enquête Démographique et de Santé du Bénin 2011-2012. Calverton, Maryland, USA : INSAE et ICF International; 2013.
BTN	Bhutan	2010	2012	53		5	2144	1	Ministry of Health. 2012 National Health Survey (NHS). Thimphu, Bhutan; 2014.
BOL	Bolivia (Plurination al State of)	1991	1994	31.7		1	3580	1	Instituto Nacional de Estadística, Ministerio de Desarrollo Sostenible, Medio Ambiente Secretaría Nacional de Planificación, Macro International Inc. Encuesta Nacional de Demografía y Salud 1994. Calverton, Maryland, USA: Macro International Inc.; 1994.
BOL	Bolivia (Plurination al State of)	1995	1998	44.9		1	4106	1	Instituto Nacional de Estadística, Ministerio de Hacienda, Macro International Inc. Encuesta Nacional de Demografía y Salud 1998. Calverton, Maryland, USA: Macro International Inc.; 1998.
BOL	Bolivia (Plurination al State of)	1998	2003	51.4		1	7261	1	Ministerio de Salud y Deportes (MSD), Programa Reforma de Salud (PRS), Instituto Nacional de Estadística (INE), ORC Macro. Encuesta Nacional de Demografía y Salud ENDSA 2003. Calverton, Maryland, USA: ORC Macro; 2004.

ISO3	Country name	Data coverage period start year	Data coverage period end year	Coverage of early ANC visits (%) (women 15-49 years)	No. of pregnant women (15-49 years) having early ANC visit	Timing of first visit codes: 1=<4 mo; 2=<14 weeks; 3=<15 weeks; 4=<16 weeks; 5=1st trimester; 6=<6 mo; 7<12 weeks; 8<13 weeks; 9<20 weeks; 10<3 mo	Sample size	Source code: 1=survey; 2=adm.; 3= perinatal	Sources
BOL	Bolivia (Plurinational State of)	2003	2008	61.8		1	6472	1	Ministerio de Salud y Deportes (MSD), Programa Reforma de Salud (PRS), Instituto Nacional de Estadística (INE), Macro International. Encuesta Nacional de Demografía y Salud ENDSA 2008. Calverton, Maryland, USA: Macro International; 2009.
BOL	Bolivia (Plurinational State of)	2007	2012	59.4		7	9961	1	Unidad de Análisis de Políticas Sociales y Económicas (UDAPE), Ministerio de Salud. Encuesta de Evaluación de Salud y Nutrición 2012: Informe de Resultados. UDAPE, La Paz; 2014.
BWA	Botswana	2005	2007	53		1	1397	1	Central Statistics Office, UNICEF. 2007 Botswana Family Health Survey - IV Report. Gaborone, Botswana; 2009.
BRA	Brazil	2001	2006	83.6		5	4883	1	Ministério da Saúde. Pesquisa Nacional de Demografia e Saúde da Criança e da Mulher (PNDS 2006). Dimensões do Processo Reprodutivo e da Saúde da Criança. Brasília-DF, Brazil; 2009.
BRA	Brazil	2011	2012	75.8		4	23894	3	Viellas EF, Domingues RM, Dias MA, Gama SG, Theme Filha MM, Costa JV et al. Prenatal care in Brazil. Cad Saude Publica. 2014;30 Suppl 1:S1-15.
BFA	Burkina Faso	1998	1993	20.5		1	6302	1	Institut National de la Statistique et de la Démographie, Macro International Inc. Enquête Démographique et de Santé Burkina Faso 1993. Calverton, Maryland USA: Macro International Inc., 1994.
BFA	Burkina Faso	1993	1999	21.6		1	6218	1	Institut National de la Statistique et de la Démographie, et Macro International Inc. Enquête Démographique et de Santé, Burkina Faso 1998-1999. Calverton, Maryland, USA: Macro International Inc.; 2000.
BFA	Burkina Faso	1998	2003	26.9		1	7428	1	Institut National de la Statistique et de la Démographie (INSD) et ORC Macro. Enquête Démographique et de Santé du Burkina Faso 2003. Calverton, Maryland, USA: INSD et ORC Macro; 2004.
BFA	Burkina Faso	2005	2010	41.2		1	10487	1	Institut de Statistiques et d'Études Économiques du Burundi (ISTEEBU), Ministère de la Santé Publique et de la Lutte contre le Sida [Burundi] (MSPLS), et ICF International. Enquête Démographique et de

ISO3	Country name	Data coverage period start year	Data coverage period end year	Coverage of early ANC visits (%) (women 15-49 years)	No. of pregnant women (15-49 years) having early ANC visit	Timing of first visit codes: 1=<4 mo; 2=<14 weeks; 3=<15 weeks; 4=<16 weeks; 5=1st trimester; 6=<6 mo; 7<12 weeks; 8<13 weeks; 9<20 weeks; 10<3 mo	Sample size	Source code: 1=survey; 2=adm.; 3= perinatal	Sources
									Santé Burundi 2010. Bujumbura, Burundi : ISTEEBU, MSPLS, et ICF International; 2012.
BDI	Burundi	2005	2010	20.6		1	5063	1	Institut de Statistiques et d'Études Économiques du Burundi (ISTEEBU), Ministère de la Santé Publique et de la Lutte contre le Sida [Burundi] (MSPLS), et ICF International. Enquête Démographique et de Santé Burundi 2010. Bujumbura, Burundi : ISTEEBU, MSPLS, et ICF International; 2012.
CPV	Cabo Verde	1993	1998	45.4		1	4820	1	Instituto Nacional de Estatística (INE) [Cabo Verde], CDC. Inquérito Demográfico e de Saúde Reprodutiva 1998. Relatório Final. Cabo Verde; 1999.
CPV	Cabo Verde	2000	2005	53.8		1	2010	1	Instituto Nacional de Estatística (INE) [Cabo Verde], Ministério da Saúde, Macro International. Segundo Inquérito Demográfico e de Saúde Reprodutiva, Cabo Verde, IDSR-II, 2005. Calverton, Maryland, USA: INE; 2008.
KHM	Cambodia	1993	1998	19.8		6	4754	1	National Institute of Public Health, Ministry of Health, Phnom Penh, Cambodia and Macro International Inc. Calverton, Maryland, USA. National Health Survey 1998. Phnom Penh, Cambodia, 1999.
KHM	Cambodia	1995	2000	10		1	5714	1	National Institute of Statistics, Directorate General for Health [Cambodia], and ORC Macro. Cambodia Demographic and Health Survey 2000. Phnom Penh, Cambodia, and Calverton, Maryland USA: National Institute of Statistics, Directorate General for Health, and ORC Macro; 2001.
KHM	Cambodia	2000	2005	23.1		1	5865	1	National Institute of Public Health, National Institute of Statistics [Cambodia] and ORC Macro. Cambodia Demographic and Health Survey 2005. Phnom Penh, Cambodia and Calverton, Maryland, USA: National Institute of Public Health, National Institute of Statistics and ORC Macro, 2006.
KHM	Cambodia	2005	2010	59.4		1	5793	1	National Institute of Statistics, Directorate General for Health, and ICF Macro. Cambodia Demographic and Health Survey 2010. Phnom Penh, Cambodia and Calverton, Maryland, USA: National Institute of Statistics, Directorate General for Health, and ICF

ISO3	Country name	Data coverage period start year	Data coverage period end year	Coverage of early ANC visits (%) (women 15-49 years)	No. of pregnant women (15-49 years) having early ANC visit	Timing of first visit codes: 1=<4 mo; 2=<14 weeks; 3=<15 weeks; 4=<16 weeks; 5=1st trimester; 6=<6 mo; 7<12 weeks; 8<13 weeks; 9<20 weeks; 10<3 mo	Sample size	Source code: 1=survey; 2=adm.; 3= perinatal	Sources
									Macro, 2011.
KHM	Cambodia	2009	2014	79		1	5704	1	National Institute of Statistics, Directorate General for Health, and ICF International. Cambodia Demographic and Health Survey 2014. Phnom Penh, Cambodia, and Rockville, Maryland, USA: National Institute of Statistics, Directorate General for Health, and ICF International; 2015.
CMR	Cameroon	1986	1991	32.7		1	3456	1	Direction Nationale du Deuxième Recensement Général de la Population et de l'Habitat, Macro International Inc. Enquête Démographique et de Santé du Cameroun 1991. Calverton, Columbia, Maryland, USA: Macro International Inc.; 1992.
CMR	Cameroon	1995	1998	31.5		1	2469	1	Médard F, Ndonou R, Libité PR, Tsafack M, Wakou R, Ghapoutsa A et al. Enquête Démographique et de Santé, Cameroun 1998. Calverton, Maryland, USA: Bureau Central des Recensements et des Études de Population et Macro International Inc.; 1999.
CMR	Cameroon	1999	2004	34.7		1	5303	1	Institut National de la Statistique (INS) et ORC Macro. Enquête Démographique et de Santé du Cameroun 2004. Calverton, Maryland, USA: INS et ORC Macro; 2005.
CMR	Cameroon	2006	2011	34.3		1	7647	1	Institut National de la Statistique (INS) et ICF. International. Enquête Démographique et de Santé et à Indicateurs Multiples du Cameroun 2011. Calverton, Maryland, USA: INS et ICF International; 2012.
CAN	Canada	2006	2007	94.9		2	6421	3	Public Health Agency of Canada. What Mothers Say: The Canadian Maternity Experience Survey. Ottawa; 2009.
TCD	Chad	1991	1997	15.7		1	7498	1	Bandoumal O, Nodjimadji K, Ngoniri JN,Ngakoutou N, Ignégongba K, Tokindang JS et al. Enquête Démographique et de Santé, Tchad 1996-1997. Calverton, Maryland, USA: Bureau Central du Recensement et Macro International Inc.; 1998.
TCD	Chad	1999	2004	16.7		1	3720	1	Bandoumal O, Nodjimadji K, Bagamla T, Madnodji R, Tokindang JS, Ngakoutou N et al. Enquête Démographique et de Santé Tchad 2004. Calverton,

ISO3	Country name	Data coverage period start year	Data coverage period end year	Coverage of early ANC visits (%) (women 15-49 years)	No. of pregnant women (15-49 years) having early ANC visit	Timing of first visit codes: 1=<4 mo; 2=<14 weeks; 3=<15 weeks; 4=<16 weeks; 5=1st trimester; 6=<6 mo; 7<12 weeks; 8<13 weeks; 9<20 weeks; 10<3 mo	Sample size	Source code: 1=survey; 2=adm.; 3= perinatal	Sources
									Maryland, USA: INSEED et ORC Macro; 2005.
COL	Colombia	1998	1990	62.1		1	3713	1	Asociación Probienestar de la Familia Colombiana (Profamilia), Macro International Inc. Encuesta Nacional de Demografía y Salud 1990. Columbia, Maryland, USA: Macro International Inc.;1991.
COL	Colombia	1990	1995	63.5		1	5050	1	Asociación Probienestar de la Familia Colombiana (Profamilia), Macro International Inc. Encuesta Nacional de Demografía y Salud 1995. Calverton, Maryland, USA: Macro International Inc.; 1995.
COL	Colombia	1995	2000	68.2		1	3547	1	Asociación Probienestar de la Familia Colombiana (Profamilia). Salud Sexual y Reproductiva en Colombia. Resultados Encuesta Nacional de Demografía y Salud 2000. Bogotá, Colombia; 2000.
COL	Colombia	2000	2005	70.5		1	11062	1	Asociación Probienestar de la Familia Colombiana (Profamilia). Salud Sexual y Reproductiva en Colombia. Encuesta Nacional de Demografía y Salud 2005. Bogotá, Colombia; 2005.
COL	Colombia	2005	2010	77		1	13254	1	Asociación Probienestar de la Familia Colombiana (Profamilia). Encuesta Nacional de Demografía y Salud 2010. Bogotá, Colombia; 2010.
COM	Comoros	1993	1996	34.4		1	1145	1	Kassim AM, Schoemaker J, Banère M. Enquête Démographique et de Santé, Comores 1996. Calverton, Maryland, USA: Centre National de Documentation et de Recherche Scientifique et Macro International Inc.; 1997.
COM	Comoros	2007	2012	57.8		1	1921	1	Direction Générale de la Statistique et de la Prospective (DGSP) Comores et ICF International. l'Enquête Démographique et de Santé et à Indicateurs Multiples de l'Union des Comores (EDSC-MICS 12). ICF International: Calverton, MD, USA; 2014.
COG	Congo	2000	2005	46.6		1	3568	1	Centre National de la Statistique et des Études Économiques (CNSEE) et ORC Macro. Enquête Démographique et de Santé du Congo 2005. Calverton, Maryland, USA: CNSEE et ORC Macro; 2006.

ISO3	Country name	Data coverage period start year	Data coverage period end year	Coverage of early ANC visits (%) (women 15-49 years)	No. of pregnant women (15-49 years) having early ANC visit	Timing of first visit codes: 1=<4 mo; 2=<14 weeks; 3=<15 weeks; 4=<16 weeks; 5=1st trimester; 6=<6 mo; 7<12 weeks; 8<13 weeks; 9<20 weeks; 10<3 mo	Sample size	Source code: 1=survey; 2=adm.; 3= perinatal	Sources
COG	Congo	2006	2011	46.1		1	5479	1	Centre Nationale de la Statistique et des Études Économiques (CNSEE) [Congo] et ICF International. Enquête Démographique et de Santé du Congo (EDSC-II) 2011-2012. Calverton, Maryland, USA: CNSEE et ICF International, 2013.
CRI	Costa Rica	1987	1993	75		1	1566	1	Caja Costarricense del Seguro Social. Fecundidad y formación de la Familia. Encuesta Nacional de Salud Reproductiva del 1993. San José, Costa Rica; 1994.
CRI	Costa Rica	2005	2010	88.7		1	326	1	Costa Rica. Ministerio de Salud. Informe de los Resultados de la Encuesta de Salud Sexual y Reproductiva 2010. San José, Costa Rica: El Ministerio; 2011.
CIV	Côte d'Ivoire	1991	1994	22.6		1	3989	1	Institut National de la Statistique, Ministère Délégué Auprès du Premier Ministre, Chargé de l'Economie, des Finances et du Plan, Macro International Inc. Enquête Démographique et de Santé 1994. Calverton, Maryland, USA: Macro International Inc.; 1995.
CIV	Côte d'Ivoire	1993	1999	28.9		1	2224	1	Institut National de la Statistique [Côte d'Ivoire] et ORC Macro. Enquête Démographique et de Santé, Côte d'Ivoire 1998-1999. Calverton, Maryland USA : Institut National de la Statistique et ORC Macro; 2001.
CIV	Côte d'Ivoire	2000	2005	30.5		1	2553	1	Institut National de la Statistique (INS) et Ministère de la Lutte contre le Sida [Côte d'Ivoire] et ORC Macro. Enquête sur les Indicateurs du Sida, Côte d'Ivoire 2005. Calverton, Maryland, USA: INS et ORC Macro; 2006.
CIV	Côte d'Ivoire	2006	2012	29.8		1	4843	1	Institut National de la Statistique (INS) et ICF International. Enquête Démographique et de Santé et à Indicateurs Multiples de Côte d'Ivoire 2011-2012. Calverton, Maryland, USA: INS et ICF International; 2012.
HRV	Croatia	2005	2005	72.7		8	42574	2	Croatian National Institute of Public Health. Croatian Health Service Yearbook 2005. Zagreb; 2006.
HRV	Croatia	2006	2006	72.7		8	41600	2	Croatian National Institute of Public Health. Croatian Health Service Yearbook 2006. Zagreb; 2007.

ISO3	Country name	Data coverage period start year	Data coverage period end year	Coverage of early ANC visits (%) (women 15-49 years)	No. of pregnant women (15-49 years) having early ANC visit	Timing of first visit codes: 1=<4 mo; 2=<14 weeks; 3=<15 weeks; 4=<16 weeks; 5=1st trimester; 6=<6 mo; 7<12 weeks; 8<13 weeks; 9<20 weeks; 10<3 mo	Sample size	Source code: 1=survey; 2=adm.; 3= perinatal	Sources
HRV	Croatia	2007	2007	67.3		8	42136	2	Croatian National Institute of Public Health. Croatian Health Service Yearbook 2007. Zagreb; 2008.
HRV	Croatia	2008	2008	69.9		8	43776	2	Croatian National Institute of Public Health. Croatian Health Service Yearbook 2008. Zagreb; 2009.
HRV	Croatia	2009	2009	71.3		8	43515	2	Croatian National Institute of Public Health. Croatian Health Service Yearbook 2009. Zagreb; 2010.
HRV	Croatia	2010	2010	72.7		8	43209	2	Croatian National Institute of Public Health. Croatian Health Service Yearbook 2010. Zagreb; 2011.
HRV	Croatia	2011	2011	73.5		8	41163	2	Croatian National Institute of public Health. Croatian Health Service Yearbook 2011. Zagreb; 2012.
HRV	Croatia	2012	2012	74.4		8	41627	2	Croatian National Institute of Public Health. Croatian Health Service Yearbook 2012. Zagreb; 2013.
HRV	Croatia	2013	2013	70.7		8	39966	2	Croatian National Institute of Public Health. Croatian Health Service Yearbook 2013. Zagreb; 2014.
HRV	Croatia	2014	2014	71.2		8	39631	2	Croatian National Institute of Public Health. Croatian Health Service Yearbook 2014. Zagreb; 2015.
CUB	Cuba	2012	2014	92.4		1	635	1	Dirección de Registros Médicos, Estadísticas de Salud, Ministerio de Salud Pública. Encuesta de Indicadores Múltiples por Conglomerados. Cuba, 2014. Informe final. La Habana, Cuba; 2015.
CYP	Cyprus	2007	2007	61	1308	3	2144	2	Ministry of Health. Perinatal Health Indicators, Cyprus Public Maternity Units 2007-2013. Cyprus; 2015.
CYP	Cyprus	2008	2008	58.8	1392	3	2377	2	Ministry of Health. Perinatal Health Indicators, Cyprus Public Maternity Units 2007-2013. Cyprus; 2015.
CYP	Cyprus	2009	2009	56.8	1615	3	2846	2	Ministry of Health. Perinatal Health Indicators, Cyprus Public Maternity Units 2007-2013. Cyprus; 2015.
CYP	Cyprus	2010	2010	58.4	1872	3	3216	2	Ministry of Health. Perinatal Health Indicators, Cyprus Public Maternity Units 2007-2013. Cyprus; 2015.

ISO3	Country name	Data coverage period start year	Data coverage period end year	Coverage of early ANC visits (%) (women 15-49 years)	No. of pregnant women (15-49 years) having early ANC visit	Timing of first visit codes: 1=<4 mo; 2=<14 weeks; 3=<15 weeks; 4=<16 weeks; 5=1st trimester; 6=<6 mo; 7<12 weeks; 8<13 weeks; 9<20 weeks; 10<3 mo	Sample size	Source code: 1=survey; 2=adm.; 3= perinatal	Sources
CYP	Cyprus	2011	2011	60.8	2088	3	3440	2	Ministry of Health. Perinatal Health Indicators, Cyprus Public Maternity Units 2007-2013. Cyprus; 2015.
CYP	Cyprus	2012	2012	60.6	2385	3	3950	2	Ministry of Health. Perinatal Health Indicators, Cyprus Public Maternity Units 2007-2013. Cyprus; 2015.
CYP	Cyprus	2013	2013	66.3	2354	3	3564	2	Ministry of Health. Perinatal Health Indicators, Cyprus Public Maternity Units 2007-2013. Cyprus; 2015.
CZE	Czechia	1987	1993	94.1		1	1382	1	Czech Statistical Office, Centers for Disease Control and Prevention (CDC), USA. 1993 Czech Republic Reproductive Health Survey. Final Report. CDC, USA; 1995.
CZE	Czechia	2001	2001	81.9	73095	8	89303	2	Institute of Health Information and Statistics of the Czech Republic. Rodička a novorozenec 2001 [Mother and Newborn 2001]. Prague, Czech Republic; 2002.
CZE	Czechia	2002	2002	81.6	74688	8	91534	2	Institute of Health Information and Statistics of the Czech Republic. Rodička a novorozenec 2002 [Mother and Newborn 2002]. Prague, Czech Republic, 2004.
CZE	Czechia	2003	2003	81.6	75351	8	92387	2	Institute of Health Information and Statistics of the Czech Republic. Rodička a novorozenec 2003 [Mother and Newborn 2003]. Prague, Czech Republic; 2004.
CZE	Czechia	2004	2004	81.8	78574	8	96098	2	Institute of Health Information and Statistics of the Czech Republic. Rodička a novorozenec 2004 [Mother and Newborn 2004]. Prague, Czech Republic; 2005.
CZE	Czechia	2005	2005	81.7	82150	8	100519	2	Institute of Health Information and Statistics of the Czech Republic. Rodička a novorozenec 2005 [Mother and Newborn 2005]. Prague, Czech Republic; 2006.
CZE	Czechia	2006	2006	81	84338	8	104129	2	Institute of Health Information and Statistics of the Czech Republic. Rodička a novorozenec 2006 [Mother and Newborn 2006]. Prague, Czech Republic; 2007.

ISO3	Country name	Data coverage period start year	Data coverage period end year	Coverage of early ANC visits (%) (women 15-49 years)	No. of pregnant women (15-49 years) having early ANC visit	Timing of first visit codes: 1=<4 mo; 2=<14 weeks; 3=<15 weeks; 4=<16 weeks; 5=1st trimester; 6=<6 mo; 7<12 weeks; 8<13 weeks; 9<20 weeks; 10<3 mo	Sample size	Source code: 1=survey; 2=adm.; 3= perinatal	Sources
CZE	Czechia	2007	2007	79.3	88793	8	111988	2	Institute of Health Information and Statistics of the Czech Republic. Rodička a novorozenec 2007 [Mother and Newborn 2007]. Prague, Czech Republic; 2008.
CZE	Czechia	2008	2008	77.8	91275	8	117317	2	Institute of Health Information and Statistics of the Czech Republic. Rodička a novorozenec 2008 [Mother and Newborn 2008]. Prague, Czech Republic; 2009.
CZE	Czechia	2009	2009	78.2	90824	8	116090	2	Institute of Health Information and Statistics of the Czech Republic. Rodička a novorozenec 2009 [Mother and Newborn 2009]. Prague, Czech Republic; 2009.
CZE	Czechia	2010	2010	79.2	90583	8	114407	2	Institute of Health Information and Statistics of the Czech Republic. Rodička a novorozenec 2010 [Mother and Newborn 2010]. Prague, Czech Republic; 2011.
CZE	Czechia	2011	2011	79.5	84608	8	106392	2	Institute of Health Information and Statistics of the Czech Republic. Rodička a novorozenec 2011 [Mother and Newborn 2011]. Prague, Czech Republic; 2012.
CZE	Czechia	2012	2012	79.9	84483	8	105790	2	Institute of Health Information and Statistics of the Czech Republic. Rodička a novorozenec 2012 [Mother and Newborn 2012]. Prague, Czech Republic; 2013.
CZE	Czechia	2013	2013	78.8	81898	8	103902	2	Institute of Health Information and Statistics of the Czech Republic. Rodička a novorozenec 2013 [Mother and Newborn 2013]. Prague, Czech Republic; 2015.
COD	Democratic Republic of the Congo	2002	2007	18.6		1	4765	1	Ministère du Plan et Macro International. Enquête Démographique et de Santé, République Démocratique du Congo 2007. Calverton, Maryland, USA.: Ministère du Plan et Macro International; 2008.
COD	Democratic Republic of the Congo	2008	2014	17		1	11065	1	Ministère du Plan et Suivi de la Mise en oeuvre de la Révolution de la Modernité (MPSMRM), Ministère de la Santé Publique (MSP) et ICF International. Enquête Démographique et de Santé en République Démocratique du Congo 2013-2014. Rockville, Maryland, USA : MPSMRM, MSP et ICF

ISO3	Country name	Data coverage period start year	Data coverage period end year	Coverage of early ANC visits (%) (women 15-49 years)	No. of pregnant women (15-49 years) having early ANC visit	Timing of first visit codes: 1=<4 mo; 2=<14 weeks; 3=<15 weeks; 4=<16 weeks; 5=1st trimester; 6=<6 mo; 7<12 weeks; 8<13 weeks; 9<20 weeks; 10<3 mo	Sample size	Source code: 1=survey; 2=adm.; 3= perinatal	Sources
									International; 2014.
DJI	Djibouti	1997	2002	80.9	1105	6	1365	1	Gouvernement de Djibouti, Ligue des États Arabes - PAPFAM. Enquête Djiboutienne sur la Santé de la Famille 2002. PAPFAM - Rapport Final. Djibouti; 2004.
DJI	Djibouti	2007	2012	64.3		1	1849	1	Ministère de la Santé, Direction de la Statistique et des Etudes Démographiques, Ligue des États Arabes. Deuxième Enquête Djiboutienne sur la Santé de la Famille (EDSF/PAPFAM 2 – 2012). Le Caire, Egypte: Projet Pan Arabe sur la Santé de la Famille; 2012.
DOM	Dominican Republic	1986	1991	72.5		1	3820	1	Asociación Dominicana Pro-Bienestar de la Familia (PROFAMILIA), Oficina Nacional de Planificación, Macro International Inc. Encuesta Demográfica y de Salud de la República Dominicana 1991 (ENDESA-91). Columbia, Maryland, USA: Macro International Inc., 1992.
DOM	Dominican Republic	1991	1996	78.9		1	4379	1	Centro de Estudios Sociales y Demográficos (CESDEM), Asociación Dominicana Pro-Bienestar de la Familia (PROFAMILIA), Oficina Nacional de Planificación (ONAPLAN), Macro International Inc. Encuesta Demográfica y de Salud de la República Dominicana 1996 (ENDESA-96). Columbia, Maryland, USA: Macro International Inc.; 1997.
DOM	Dominican Republic	1997	2002	82.3		1	7866	1	Centro de Estudios Sociales y Demográficos (CESDEM), Secretaría de Estado de Salud, Pública y Asistencia Social (SESPAS), ORC Macro. Encuesta Demográfica y de Salud 2002 (ENDESA 2002). Calverton, Maryland, USA: ORC Macro; 2003.
DOM	Dominican Republic	2002	2007	81.9		1	8120	1	Centro de Estudios Sociales y Demográficos (CESDEM) y Macro International Inc. Encuesta Demográfica y de Salud 2007. Santo Domingo, República Dominicana: CESDEM y Macro International Inc.; 2008.
DOM	Dominican Republic	2008	2013	83.4		1	2916	1	Centro de Estudios Sociales y Demográficos (CESDEM) y ICF International. Encuesta Demográfica y de Salud 2013. Santo Domingo,

ISO3	Country name	Data coverage period start year	Data coverage period end year	Coverage of early ANC visits (%) (women 15-49 years)	No. of pregnant women (15-49 years) having early ANC visit	Timing of first visit codes: 1=<4 mo; 2=<14 weeks; 3=<15 weeks; 4=<16 weeks; 5=1st trimester; 6=<6 mo; 7<12 weeks; 8<13 weeks; 9<20 weeks; 10<3 mo	Sample size	Source code: 1=survey; 2=adm.; 3= perinatal	Sources
									República Dominicana: CESDEM y ICF International; 2014.
ECU	Ecuador	1989	1994	75.9		5	6599	1	Centro de Estudios de Población y Paternidad Responsable (CEPAR), División de Salud Reproductiva de los Centros para el Control de Enfermedades (CDC). Encuesta de Salud Materna e Infantil (ENDEMAIN-94). Quito, Ecuador; 1995.
ECU	Ecuador	1994	1999	75.3		5	6806	1	Centro de Estudios de Población y Desarrollo Social (CEPAR), Centros para el Control y Prevención de Enfermedades (CDC). Encuesta Demográfica y de Salud Materna e Infantil (ENDEMAIN-99). Informe General. Quito, Ecuador; 2001.
ECU	Ecuador	1999	2004	64.6		5	6140	1	Centro de Estudios de Población y Desarrollo Social (CEPAR), Centros para el Control y Prevención de Enfermedades (CDC). Encuesta Demográfica y de Salud Materna e Infantil (ENDEMAIN-2004). Informe Final. Quito, Ecuador; 2005.
EGY	Egypt	1987	1992	39.9		1	8626	1	El-Zanaty FH, Sayed H AA, Zaky Hassan HM, Way AA. Egypt Demographic and Health Survey 1992. Calverton, Maryland [USA]: National Population Council [Egypt] and Macro International Inc.; 1993.
EGY	Egypt	1990	1995	32.6		1	11454	1	El-Zanaty F, Hussein EM, Shawky GA, Way AA, Kishor S. Egypt Demographic and Health Survey 1995. Calverton, Maryland [USA]: National Population Council [Egypt] and Macro International Inc.; 1996.
EGY	Egypt	1995	2000	42.9		1	11361	1	El-Zanaty F, Way A. Egypt Demographic and Health Survey 2000. Calverton, Maryland [USA]: Ministry of Health and Population [Egypt], National Population Council and ORC Macro; 2001.
EGY	Egypt	1998	2003	51.9		1	6314	1	El-Zanaty F, Way AA. 2003 Egypt Interim Demographic and Health Survey. Cairo Egypt: Ministry of Health and Population [Egypt], National Population Council, El-Zanaty and Associates, and ORC Macro; 2004.
EGY	Egypt	2002	2005	54.9		1	9845	1	El-Zanaty F, Way A. Egypt Demographic and Health Survey 2005. Cairo, Egypt: Ministry of Health and Population, National Population Council, El-Zanaty

ISO3	Country name	Data coverage period start year	Data coverage period end year	Coverage of early ANC visits (%) (women 15-49 years)	No. of pregnant women (15-49 years) having early ANC visit	Timing of first visit codes: 1=<4 mo; 2=<14 weeks; 3=<15 weeks; 4=<16 weeks; 5=1st trimester; 6=<6 mo; 7<12 weeks; 8<13 weeks; 9<20 weeks; 10<3 mo	Sample size	Source code: 1=survey; 2=adm.; 3= perinatal	Sources
									and Associates, and ORC Macro; 2006.
EGY	Egypt	2003	2008	61.1		1	7896	1	El-Zanaty F, Way A. Egypt Demographic and Health Survey 2008. Cairo, Egypt: Ministry of Health, El-Zanaty and Associates, and Macro International; 2009.
EGY	Egypt	2009	2014	75		1	11391	1	Ministry of Health and Population [Egypt], El-Zanaty and Associates [Egypt], and ICF International. Egypt Demographic and Health Survey 2014. Cairo, Egypt and Rockville, Maryland, USA: Ministry of Health and Population and ICF International; 2015.
SLV	El Salvador	1988	1993	45.7		1	2930	1	Asociación Demográfica Salvadoreña (ADS), Division of Reproductive Health of the Centers for Disease Control and Prevention (CDC). Encuesta Nacional de Salud Familiar de 1993 (FESAL-93). San Salvador, El Salvador; 1994.
SLV	El Salvador	1993	1998	57.2		1	8482	1	Asociación Demográfica Salvadoreña (ADS), Division of Reproductive Health of the Centers for Disease Control and Prevention (CDC) . Encuesta Nacional de Salud Familiar de 1998 (FESAL-98). Informe Final. San Salvador, El Salvador; 2000.
SLV	El Salvador	1997	2003	66		1	5850	1	Asociación Demográfica Salvadoreña (ADS), Division of Reproductive Health of the Centers for Disease Control and Prevention (CDC). Encuesta Nacional de Salud Familiar de 2002/03 (FESAL-2002/03). Informe Final. San Salvador, El Salvador; 2004.
SLV	El Salvador	2003	2008	76.8		1	5169	1	Asociación Demográfica Salvadoreña (ADS), Centros para el Control, Prevención de Enfermedades (CDC). Encuesta Nacional de Salud Familiar de 2008 (FESAL-2008). Informe Final. San Salvador, El Salvador; 2009.
GNQ	Equatorial Guinea	2006	2011	63.7		1	1835	1	Ministerio de Sanidad y Bienestar Social, Ministerio de Economía, Planificación e Inversiones Públicas, ICF International. Encuesta Demográfica y de Salud (EDSGE-I) 2011. Calverton, Maryland, USA; 2012.

ISO3	Country name	Data coverage period start year	Data coverage period end year	Coverage of early ANC visits (%) (women 15-49 years)	No. of pregnant women (15-49 years) having early ANC visit	Timing of first visit codes: 1=<4 mo; 2=<14 weeks; 3=<15 weeks; 4=<16 weeks; 5=1st trimester; 6=<6 mo; 7<12 weeks; 8<13 weeks; 9<20 weeks; 10<3 mo	Sample size	Source code: 1=survey; 2=adm.; 3= perinatal	Sources
ERI	Eritrea	1992	1995	12		1	2580	1	National Statistics Office [Eritrea] and Macro International Inc. Eritrea Demographic and Health Survey, 1995. Calverton, Maryland: National Statistics Office and Macro International Inc.; 1997.
ERI	Eritrea	1997	2002	22.2		1	4175	1	National Statistics and Evaluation Office (NSEO) [Eritrea] and ORC Macro. Eritrea Demographic and Health Survey 2002. Calverton, Maryland, USA: National Statistics and Evaluation Office and ORC Macro; 2003.
ERI	Eritrea	2005	2010	26.5		1	12498	1	National Statistics Office (NSO) [Eritrea] and Fafo AIS. Eritrea Population and Health Survey 2010. Asmara, Eritrea: National Statistics Office and Fafo Institute for Applied International Studies; 2013.
EST	Estonia	1992	1992	73.8	12936	4	17534	2	National Institute for Health Development Estonia. http://www.tai.ee/en/r-and-d/registers/estonian-medical-birth-registry-and-estonian-abortion-registry/statistics.
EST	Estonia	1993	1993	74.2	10897	4	14690	2	National Institute for Health Development Estonia. http://www.tai.ee/en/r-and-d/registers/estonian-medical-birth-registry-and-estonian-abortion-registry/statistics.
EST	Estonia	1994	1994	75.9	10330	4	13595	2	National Institute for Health Development Estonia. http://www.tai.ee/en/r-and-d/registers/estonian-medical-birth-registry-and-estonian-abortion-registry/statistics.
EST	Estonia	1995	1995	79	10311	4	13042	2	National Institute for Health Development Estonia. http://www.tai.ee/en/r-and-d/registers/estonian-medical-birth-registry-and-estonian-abortion-registry/statistics..
EST	Estonia	1996	1996	80.8	10355	4	12820	2	National Institute for Health Development Estonia. http://www.tai.ee/en/r-and-d/registers/estonian-medical-birth-registry-and-estonian-abortion-registry/statistics.
EST	Estonia	1997	1997	83	10123	4	12196	2	National Institute for Health Development Estonia. http://www.tai.ee/en/r-and-d/registers/estonian-medical-birth-registry-and-estonian-abortion-registry/statistics.

ISO3	Country name	Data coverage period start year	Data coverage period end year	Coverage of early ANC visits (%) (women 15-49 years)	No. of pregnant women (15-49 years) having early ANC visit	Timing of first visit codes: 1=<4 mo; 2=<14 weeks; 3=<15 weeks; 4=<16 weeks; 5=1st trimester; 6=<6 mo; 7<12 weeks; 8<13 weeks; 9<20 weeks; 10<3 mo	Sample size	Source code: 1=survey; 2=adm.; 3= perinatal	Sources
EST	Estonia	1998	1998	84.7	10050	4	11860	2	National Institute for Health Development Estonia. http://www.tai.ee/en/r-and-d/registers/estonian-medical-birth-registry-and-estonian-abortion-registry/statistics.
EST	Estonia	1999	1999	85.6	10353	4	12099	2	National Institute for Health Development Estonia. http://www.tai.ee/en/r-and-d/registers/estonian-medical-birth-registry-and-estonian-abortion-registry/statistics.
EST	Estonia	2000	2000	86.3	11022	4	12780	2	National Institute for Health Development Estonia. http://www.tai.ee/en/r-and-d/registers/estonian-medical-birth-registry-and-estonian-abortion-registry/statistics.
EST	Estonia	2001	2001	87.5	10802	4	12352	2	National Institute for Health Development Estonia. http://www.tai.ee/en/r-and-d/registers/estonian-medical-birth-registry-and-estonian-abortion-registry/statistics..
EST	Estonia	2002	2002	88.2	11222	4	12729	2	National Institute for Health Development Estonia. http://www.tai.ee/en/r-and-d/registers/estonian-medical-birth-registry-and-estonian-abortion-registry/statistics.
EST	Estonia	2003	2003	89.8	11506	4	12806	2	National Institute for Health Development Estonia. http://www.tai.ee/en/r-and-d/registers/estonian-medical-birth-registry-and-estonian-abortion-registry/statistics.
EST	Estonia	2004	2004	91	12503	4	13736	2	National Institute for Health Development Estonia. http://www.tai.ee/en/r-and-d/registers/estonian-medical-birth-registry-and-estonian-abortion-registry/statistics.
EST	Estonia	2005	2005	92.2	12999	4	14098	2	National Institute for Health Development Estonia. http://www.tai.ee/en/r-and-d/registers/estonian-medical-birth-registry-and-estonian-abortion-registry/statistics.
EST	Estonia	2006	2006	94	13743	4	14619	2	National Institute for Health Development Estonia. http://www.tai.ee/en/r-and-d/registers/estonian-medical-birth-registry-and-estonian-abortion-registry/statistics.
EST	Estonia	2007	2007	94.6	14691	4	15545	2	National Institute for Health Development Estonia. http://www.tai.ee/en/r-and-d/registers/estonian-medical-birth-registry-and-estonian-abortion-

ISO3	Country name	Data coverage period start year	Data coverage period end year	Coverage of early ANC visits (%) (women 15-49 years)	No. of pregnant women (15-49 years) having early ANC visit	Timing of first visit codes: 1=<4 mo; 2=<14 weeks; 3=<15 weeks; 4=<16 weeks; 5=1st trimester; 6=<6 mo; 7<12 weeks; 8<13 weeks; 9<20 weeks; 10<3 mo	Sample size	Source code: 1=survey; 2=adm.; 3= perinatal	Sources
									registry/statistics.
EST	Estonia	2008	2008	95.3	15000	4	15745	2	National Institute for Health Development Estonia. http://www.tai.ee/en/r-and-d/registers/estonian-medical-birth-registry-and-estonian-abortion-registry/statistics.
EST	Estonia	2009	2009	95.4	14780	4	15491	2	National Institute for Health Development Estonia. http://www.tai.ee/en/r-and-d/registers/estonian-medical-birth-registry-and-estonian-abortion-registry/statistics.
EST	Estonia	2010	2010	96	15034	4	15668	2	National Institute for Health Development Estonia. http://www.tai.ee/en/r-and-d/registers/estonian-medical-birth-registry-and-estonian-abortion-registry/statistics.
EST	Estonia	2011	2011	95.9	14049	4	14644	2	National Institute for Health Development Estonia. http://www.tai.ee/en/r-and-d/registers/estonian-medical-birth-registry-and-estonian-abortion-registry/statistics.
EST	Estonia	2012	2012	96.3	13578	4	14109	2	National Institute for Health Development Estonia. http://www.tai.ee/en/r-and-d/registers/estonian-medical-birth-registry-and-estonian-abortion-registry/statistics.
EST	Estonia	2013	2013	96.8	13287	4	13725	2	National Institute for Health Development Estonia. http://www.tai.ee/en/r-and-d/registers/estonian-medical-birth-registry-and-estonian-abortion-registry/statistics.
EST	Estonia	2014	2014	97.1	13182	4	13573	2	National Institute for Health Development Estonia. http://www.tai.ee/en/r-and-d/registers/estonian-medical-birth-registry-and-estonian-abortion-registry/statistics.
ETH	Ethiopia	1995	2000	6.2		1	7978	1	Central Statistical Authority [Ethiopia] and ORC Macro. Ethiopia Demographic and Health Survey 2000. Addis Ababa, Ethiopia and Calverton, Maryland, USA: Central Statistical Authority and ORC Macro; 2001.
ETH	Ethiopia	2000	2005	6.4		1	7307	1	Central Statistical Agency, and ORC Macro. Ethiopia Demographic and Health Survey 2005. Addis Ababa, Ethiopia and Calverton, Maryland, USA: Central

ISO3	Country name	Data coverage period start year	Data coverage period end year	Coverage of early ANC visits (%) (women 15-49 years)	No. of pregnant women (15-49 years) having early ANC visit	Timing of first visit codes: 1=<4 mo; 2=<14 weeks; 3=<15 weeks; 4=<16 weeks; 5=1st trimester; 6=<6 mo; 7<12 weeks; 8<13 weeks; 9<20 weeks; 10<3 mo	Sample size	Source code: 1=survey; 2=adm.; 3= perinatal	Sources
									Statistical Agency and ORC Macro; 2006.
ETH	Ethiopia	2006	2011	11.2		1	7908	1	Central Statistical Agency [Ethiopia] and ICF International. Ethiopia Demographic and Health Survey 2011. Addis Ababa, Ethiopia and Calverton, Maryland, USA: Central Statistical Agency and ICF International; 2012.
ETH	Ethiopia	2009	2014	17.5		1	3678	1	Central Statistical Agency. Ethiopia Mini Demographic and Health Survey 2014. Addis Ababa, Ethiopia; 2014.
FIN	Finland	2004	2004	95.9		3	56878	2	EURO-PERISTAT Project, with SCPE, EUROCAT, EURONEOSTAT. European Perinatal Health Report-data for 2004. 2008.
FIN	Finland	2010	2010	96.6		3	59413	2	EURO-PERISTAT Project with SCPE and EUROCAT. European Perinatal Health Report. The health and care of pregnant women and babies in Europe in 2010.2013.
FRA	France	1995	1995	95.8		5	12587	3	Blondel B, Lelong N, Kermarrec M, Goffinet F, The National Coordination Group of the National Perinatal Surveys Trends in perinatal health in France from 1995 to 2010. Results from the French National Perinatal Surveys. J Gynecol Obstet Biol Reprod (Paris). 2012;41(4):e1-e15.
FRA	France	1998	1998	95.6		5	12882	3	Blondel B, Lelong N, Kermarrec M, Goffinet F, The National Coordination Group of the National Perinatal Surveys Trends in perinatal health in France from 1995 to 2010. Results from the French National Perinatal Surveys. J Gynecol Obstet Biol Reprod (Paris). 2012;41(4):e1-e15.
FRA	France	2003	2003	95.1		5	13459	3	Blondel B, Lelong N, Kermarrec M, Goffinet F, The National Coordination Group of the National Perinatal Surveys Trends in perinatal health in France from 1995 to 2010. Results from the French National Perinatal Surveys. J Gynecol Obstet Biol Reprod (Paris). 2012;41(4):e1-e15.

ISO3	Country name	Data coverage period start year	Data coverage period end year	Coverage of early ANC visits (%) (women 15-49 years)	No. of pregnant women (15-49 years) having early ANC visit	Timing of first visit codes: 1=<4 mo; 2=<14 weeks; 3=<15 weeks; 4=<16 weeks; 5=1st trimester; 6=<6 mo; 7<12 weeks; 8<13 weeks; 9<20 weeks; 10<3 mo	Sample size	Source code: 1=survey; 2=adm.; 3= perinatal	Sources
FRA	France	2010	2010	92.2		5	13775	3	Blondel B, Lelong N, Kermarrec M, Goffinet F, The National Coordination Group of the National Perinatal Surveys Trends in perinatal health in France from 1995 to 2010. Results from the French National Perinatal Surveys. J Gynecol Obstet Biol Reprod (Paris). 2012;41(4):e1-e15.
GAB	Gabon	1995	2000	55.8		1	2766	1	Direction Générale de la Statistique et des Études Économiques (DGSEE) [Gabon] et ORC Macro. Enquête Démographique et de Santé Gabon 2000. Calverton, Maryland: Direction Générale de la Statistique et des Études Économiques, et Fonds des Nations Unies pour la Populations, et ORC Macro; 2001.
GAB	Gabon	2007	2012	63.6		1	3702	1	Direction Générale de la Statistique (DGS) et ICF International. Enquête Démographique et de Santé du Gabon 2012. Calverton, Maryland, et Libreville, Gabon: DGS et ICF International; 2013.
GMB	Gambia	2008	2013	37.7		1	5260	1	The Gambia Bureau of Statistics (GBOS) and ICF International. The Gambia Demographic and Health Survey 2013. Banjul, The Gambia, and Rockville, Maryland, USA: GBOS and ICF International; 2014.
GEO	Georgia	1993	2000	62.6		5	3050	1	National Center for Disease Control (NCDC), Georgian Ministry of Labor Health and Social Affairs (MOLHSA), Center for Medical Statistics and Information (CMSI). Reproductive Health, Centers for Disease Control and Prevention (DRH/CDC). Georgia Reproductive Health Survey 1999 (99 GERHS). Atlanta, GA, USA: CDC; 2001.
GEO	Georgia	2000	2005	70.6		5	2017	1	Serbanescu F, Imnadze P, Bokhua Z, Nutsubidze N, Jackson DB, Morris L. Reproductive Health Survey Georgia 2005: Final Report. Atlanta, GA, USA; 2007.
GEO	Georgia	2005	2010	89.8		5	2617	1	Georgian National Center for Disease Control, Centers for Disease Control and Prevention. Atlanta, GA, USA, UNICEF. Reproductive Health Survey Georgia 2010. Centers for Disease Control and Prevention. Atlanta, GA, USA; 2012.

ISO3	Country name	Data coverage period start year	Data coverage period end year	Coverage of early ANC visits (%) (women 15-49 years)	No. of pregnant women (15-49 years) having early ANC visit	Timing of first visit codes: 1=<4 mo; 2=<14 weeks; 3=<15 weeks; 4=<16 weeks; 5=1st trimester; 6=<6 mo; 7<12 weeks; 8<13 weeks; 9<20 weeks; 10<3 mo	Sample size	Source code: 1=survey; 2=adm.; 3= perinatal	Sources
DEU	Germany	2004	2004	93.9		3	636844	2	EURO-PERISTAT Project, with SCPE, EUROCAT, EURONEOSTAT. European Perinatal Health Report-data for 2004. 2008.
DEU	Germany	2010	2010	95		3	582477	2	EURO-PERISTAT Project with SCPE and EUROCAT. European Perinatal Health Report. The health and care of pregnant women and babies in Europe in 2010.2013.
GHA	Ghana	1990	1993	33.3		1	2168	1	Ghana Statistical Service (GSS) and Macro International Inc. (MI). Ghana Demographic and Health Survey 1993. Calverton, Maryland: GSS and MI; 1994.
GHA	Ghana	1993	1998	39.8		1	3194	1	Ghana Statistical Service (GSS) and Macro International Inc. (MI). Ghana Demographic and Health Survey 1998. Calverton, Maryland: GSS and MI; 1999.
GHA	Ghana	1998	2003	46.4		1	2645	1	Ghana Statistical Service (GSS), Noguchi Memorial Institute for Medical Research (NMIMR), and ORC Macro. Ghana Demographic and Health Survey 2003. Calverton, Maryland: GSS, NMIMR, and ORC Macro; 2004.
GHA	Ghana	2002	2007	52.8		1	4928	1	Ghana Statistical Service (GSS), Ghana Health Service (GHS), and Macro International. Ghana Maternal Health Survey 2007. Calverton, Maryland, USA: GSS, GHS, and Macro International; 2009.
GHA	Ghana	2003	2008	55		1	2099	1	Ghana Statistical Service (GSS), Ghana Health Service (GHS), and ICF Macro. Ghana Demographic and Health Survey 2008. Accra, Ghana: GSS, GHS, and ICF Macro; 2009.
GHA	Ghana	2009	2014	64		1	4142	1	Ghana Statistical Service (GSS), Ghana Health Service (GHS), and ICF International. Ghana Demographic and Health Survey 2014. Rockville, Maryland, USA: GSS, GHS, and ICF International; 2015.
GTM	Guatemala	1990	1995	60.1		1	9150	1	Instituto Nacional de Estadística (INE), Macro International Inc. Guatemala Encuesta Nacional de Salud Materno Infantil 1995. Calverton, Maryland, USA: Macro International Inc.; 1996.

ISO3	Country name	Data coverage period start year	Data coverage period end year	Coverage of early ANC visits (%) (women 15-49 years)	No. of pregnant women (15-49 years) having early ANC visit	Timing of first visit codes: 1=<4 mo; 2=<14 weeks; 3=<15 weeks; 4=<16 weeks; 5=1st trimester; 6=<6 mo; 7<12 weeks; 8<13 weeks; 9<20 weeks; 10<3 mo	Sample size	Source code: 1=survey; 2=adm.; 3= perinatal	Sources
GTM	Guatemala	1993	1999	53.2		1	4545	1	Instituto Nacional de Estadística (INE), Macro International Inc. Guatemala Encuesta Nacional de Salud Materno Infantil 1998-1999. Calverton, Maryland, USA: Macro International Inc.; 1999
GTM	Guatemala	1997	2002	51.1		5	7901	1	Ministerio de Salud Pública y Asistencia Social (MSPAS), Instituto Nacional de Estadística (INE). Guatemala Encuesta Nacional de Salud Materno Infantil 2002. Guatemala; 2003.
GTM	Guatemala	2003	2009	60.3		5	9264	1	Ministerio de Salud Pública y Asistencia Social (MSPAS), Instituto Nacional de Estadística (INE). Guatemala Encuesta Nacional de Salud Materno Infantil 2008-2009. Guatemala; 2009.
GIN	Guinea	1987	1992	39.5		6	5275	1	Keita ML, Bah MC, Diallo MB, Barrère B. Enquête Démographique et de Santé, Guinée 1992. Conakry, Guinée et Calverton, Maryland, USA: Direction National de la Statistique et de l'Information; 1994.
GIN	Guinea	1994	1999	35.3		1	5842	1	Direction Nationale de la Statistique [Guinée] et Macro International Inc. Enquête Démographique et de Santé, Guinée 1999. Calverton, Maryland USA: Direction Nationale de la Statistique et Macro International Inc.; 2000.
GIN	Guinea	2000	2005	33.8		1	4447	1	Direction Nationale de la Statistique (DNS) (Guinée) et ORC Macro. Enquête Démographique et de Santé, Guinée 2005. Calverton, Maryland, USA: DNS et ORC Macro; 2006.
GIN	Guinea	2002	2007	35.1		1	8192	1	Ministère de l'Economie des Finances et du Plan, UNICE, PAM and Direction Nationale de la Statistique. Enquête nationale sur l'état nutritionnel et le suivi des principaux indicateurs de survie de l'enfant. Rapport provisoire.Guinée; 2008.
GIN	Guinea	2007	2012	40		1	4995	1	Institut National de la Statistique, Ministère du Plan Ministère d'Etat de la Santé et de l'Hygiène Publique Conakry, Guinée Measure DHS. Enquête Démographique et de Santé et à Indicateurs Multiples (EDS-MICS-IV) Guinée 2012. ICF International Calverton, Maryland, U.S.A; 2013.
GUY	Guyana	2004	2009	48.8		1	1425	1	Ministry of Health (MOH), Bureau of Statistics (BOS), and ICF Macro. Guyana Demographic and Health Survey 2009. Georgetown, Guyana: MOH,

ISO3	Country name	Data coverage period start year	Data coverage period end year	Coverage of early ANC visits (%) (women 15-49 years)	No. of pregnant women (15-49 years) having early ANC visit	Timing of first visit codes: 1=<4 mo; 2=<14 weeks; 3=<15 weeks; 4=<16 weeks; 5=1st trimester; 6=<6 mo; 7<12 weeks; 8<13 weeks; 9<20 weeks; 10<3 mo	Sample size	Source code: 1=survey; 2=adm.; 3= perinatal	Sources
									BOS, and ICF Macro; 2010.
HTI	Haiti	1989	1995	41.1		1	3624	1	Cayemittes M, Rival A, Barrère B, Lerebours G, Gédéon AM. Enquête Mortalité, Morbidité et Utilisation des Services (EMMUS-II), Haïti 1994/95. Calverton, Maryland USA: Institut Hattien de l'Enfance et Macro International Inc.; 1995.
HTI	Haiti	1995	2000	46.4		1	4254	1	Cayemittes M, Placide FM, Barrère B, Mariko S, Sévère. B Enquête Mortalité, Morbidité et Utilisation des Services, Haïti 2000. Calverton, Maryland, USA: Ministère de la Santé Publique et de la Population, Institut Haïtien de l'Enfance et ORC Macro; 2001.
HTI	Haiti	2000	2006	65.1		1	4074	1	Cayemittes M, Placide M, Mariko S, Barrère B, Sévère B, Alexandre C. Enquête Mortalité, Morbidité et Utilisation des Services, Haïti, 2005-2006. Calverton, Maryland, USA: Ministère de la Santé Publique et de la Population, Institut Haïtien de l'Enfance et Macro International Inc.; 2007.
HTI	Haiti	2007	2012	59.5		1	5218	1	Cayemittes M, Busangu MF, de Dieu Bizimana J, Barrère B, Sévère B, Cayemittes V, Charles E. Enquête Mortalité, Morbidité et Utilisation des Services, Haïti, 2012. Calverton, Maryland, USA : MSPP, IHE et ICF International; 2013.
HND	Honduras	1986	1992	62.3		1	3946	1	Ministerio de Salud Pública. Encuesta Nacional de Epidemioloía y Salud Familiar (ENESF), 1991/92. Honduras, 1993.
HND	Honduras	1991	1996	54		1	4068	1	Secretaria de Salud y la Asociación Hondureña de Planificación de Familia (ASHONPLAFA), División de Salud Reproductiva de los Centros para el Control y Prevención de Enfermedades (CDC). Encuesta Nacional de Epidemiología y Salud Familiar 1996 (ENESF-96). Informe Final. Honduras; 1997.
HND	Honduras	1996	2001	56.4		1	6624	1	Secretaría de Salud de Honduras (SS), la Asociación Hondureña de Planificación de Familia (ASHONPLAFA) y la Agencia de los Estados Unidos para el Desarrollo Internacional (USAID/Honduras). Encuesta Nacional de Epidemiología y Salud Familiar (ENESF-01) y de la

ISO3	Country name	Data coverage period start year	Data coverage period end year	Coverage of early ANC visits (%) (women 15-49 years)	No. of pregnant women (15-49 years) having early ANC visit	Timing of first visit codes: 1=<4 mo; 2=<14 weeks; 3=<15 weeks; 4=<16 weeks; 5=1st trimester; 6=<6 mo; 7<12 weeks; 8<13 weeks; 9<20 weeks; 10<3 mo	Sample size	Source code: 1=survey; 2=adm.; 3= perinatal	Sources
									Encuesta Nacional de Salud Masculina (ENSM-01) 2001. Informe Final. Honduras; 2002.
HND	Honduras	2000	2006	68.7		1	7774	1	Secretaría de Salud [Honduras], Instituto Nacional de Estadística (INE) y Macro International. Encuesta Nacional de Salud y Demografía 2005-2006. Tegucigalpa, Honduras: SS, INE y Macro International; 2006.
HND	Honduras	2006	2012	77.6		1	8269	1	Secretaría de Salud [Honduras], Instituto Nacional de Estadística (INE) e ICF International. Encuesta Nacional de Salud y Demografía 2011-2012. Tegucigalpa, Honduras: SS, INE e ICF International; 2013.
IND	India	1989	1993	24		5	49369	1	International Institute for Population Sciences (IIPS). National Family Health Survey (MCH and Family Planning), India 1992-93. Bombay, India: IIPS; 1995.
IND	India	1995	1999	33		1	32393	1	International Institute for Population Sciences (IIPS). Reproductive and Child Health Project Rapid Household Survey (Phase I and II) 1998-1999. India. Mumbai, India: IIPS, 2000.
IND	India	1999	2004	40.2		1	195031	1	International Institute for Population Sciences (IIPS). District Level Household and Facility Survey (DLHS-2), 2002-04: India. Mumbai, India: IIPS; 2006.
IND	India	2000	2006	43.9		1	39677	1	International Institute for Population Sciences (IIPS) and Macro International. National Family Health Survey (NFHS-3), 2005–06: India: Volume I. Mumbai: IIPS; 2007.
IND	India	2004	2008	44.9		1	215048	1	International Institute for Population Sciences (IIPS). District Level Household and Facility Survey (DLHS-3), 2007-08: India. Mumbai, India: IIPS; 2010.
IDN	Indonesia	1986	1991	51.6		1	14355	1	Central Bureau of Statistics, National Family Planning Coordinating Board, Ministry of Health, Macro International Inc. Indonesia Demographic and Health Survey 1991. Columbia, Maryland, USA: Macro International Inc.; 1992.

ISO3	Country name	Data coverage period start year	Data coverage period end year	Coverage of early ANC visits (%) (women 15-49 years)	No. of pregnant women (15-49 years) having early ANC visit	Timing of first visit codes: 1=<4 mo; 2=<14 weeks; 3=<15 weeks; 4=<16 weeks; 5=1st trimester; 6=<6 mo; 7<12 weeks; 8<13 weeks; 9<20 weeks; 10<3 mo	Sample size	Source code: 1=survey; 2=adm.; 3= perinatal	Sources
IDN	Indonesia	1989	1994	56.9		1	16983	1	Central Bureau of Statistics (CBS) [Indonesia] and State Ministry of Population/National Family Planning Coordinating Board (NFPCB) and Ministry of Health (MOH) and Macro International Inc. (MI). Indonesia Demographic and Health Survey 1994. Calverton, Maryland: CBS and MI; 1995.
IDN	Indonesia	1992	1997	63.5		1	16217	1	Central Bureau of Statistics (CBS) [Indonesia] and State Ministry of Population/National Family Planning Coordinating Board (NFPCB) and Ministry of Health (MOH) and Macro International Inc. (MI). Indonesia Demographic and Health Survey 1997. Calverton, Maryland: CBS and MI; 1998.
IDN	Indonesia	1998	2003	72.4		1	12760	1	Badan Pusat Statistik-Statistics Indonesia (BPS) and ORC Macro. Indonesia Demographic and Health Survey 2002-2003. Calverton, Maryland, USA: BPS and ORC Macro; 2003.
IDN	Indonesia	2002	2007	75.3		1	14043	1	Statistics Indonesia (Badan Pusat Statistik-BPS) and Macro International. Indonesia Demographic and Health Survey 2007. Calverton, Maryland, USA: BPS and Macro International; 2008.
IDN	Indonesia	2007	2012	80.4		1	14782	1	Statistics Indonesia (Badan Pusat Statistik—BPS), National Population and Family Planning Board (BKKBN), and Kementerian Kesehatan (Kemenkes—MOH), and ICF International. Indonesia Demographic and Health Survey 2012. Jakarta, Indonesia: BPS, BKKBN, Kemenkes, and ICF International; 2013.
IRL	Ireland	1999	1999	48.7	25592	8	52556	2	Health Research and Information Division. The Economic and Social Research Institute. Perinatal Statistics Report 2003. Dublin, Ireland; 2006.
IRL	Ireland	2000	2000	49.8	26630	8	53429	2	Health Research and Information Division. The Economic and Social Research Institute. Perinatal Statistics Report 2003. Dublin, Ireland; 2006.
IRL	Ireland	2001	2001	46.3	26001	8	56115	2	Health Research and Information Division. The Economic and Social Research Institute. Perinatal Statistics Report 2003. Dublin, Ireland; 2006.
IRL	Ireland	2002	2002	45.5	26761	8	58752	2	Health Research and Information Division. The Economic and Social Research Institute. Perinatal Statistics Report 2003. Dublin, Ireland; 2006.

ISO3	Country name	Data coverage period start year	Data coverage period end year	Coverage of early ANC visits (%) (women 15-49 years)	No. of pregnant women (15-49 years) having early ANC visit	Timing of first visit codes: 1=<4 mo; 2=<14 weeks; 3=<15 weeks; 4=<16 weeks; 5=1st trimester; 6=<6 mo; 7<12 weeks; 8<13 weeks; 9<20 weeks; 10<3 mo	Sample size	Source code: 1=survey; 2=adm.; 3= perinatal	Sources
IRL	Ireland	2003	2003	45	26923	8	59837	2	Health Research and Information Division. The Economic and Social Research Institute. Perinatal Statistics Report 2003. Dublin, Ireland; 2006.
IRL	Ireland	2004	2004	47.8	28771	8	60186	2	Health Research and Information Division. The Economic and Social Research Institute.. Perinatal Statistics Report 2004. Dublin, Ireland; 2007.
IRL	Ireland	2005	2005	51.1	30485	8	59321	2	Health Research and Information Division. The Economic and Social Research Institute.. Perinatal Statistics Report 2005. Dublin, Ireland; 2008.
IRL	Ireland	2006	2006	52.8	33558	8	63555	2	Health Research and Information Division. The Economic and Social Research Institute. Perinatal Statistics Report 2006. Dublin, Ireland; 2008.
IRL	Ireland	2007	2007	52.8	33633	8	69318	2	Health Research and Information Division. The Economic and Social Research Institute. Perinatal Statistics Report 2007. Dublin, Ireland; 2009.
IRL	Ireland	2008	2008	54.3	39448	8	72582	2	Health Research and Information Division. The Economic and Social Research Institute. Perinatal Statistics Report 2008. Dublin, Ireland; 2010.
IRL	Ireland	2009	2009	55	40274	8	73249	2	Health Research and Information Division. The Economic and Social Research Institute. Perinatal Statistics Report 2009. Dublin, Ireland; 2011.
IRL	Ireland	2010	2010	62.2	45234	8	72709	2	Health Research and Information Division. The Economic and Social Research Institute. Perinatal Statistics Report 2010. Dublin, Ireland; 2012.
IRL	Ireland	2011	2011	65.7	46822	8	71375	2	Health Research and Information Division. The Economic and Social Research Institute. Perinatal Statistics Report 2012. Dublin, Ireland, 2012.
IRL	Ireland	2012	2012	68.6	47467	8	69188	2	Health Research and Information Division, the Economic and Social Research Institute. Perinatal Statistics Report 2012. Dublin, Ireland; 2013.
IRL	Ireland	2013	2013	70	44465	8	66386	2	Health Research and Information Division, the Economic and Social Research Institute. Perinatal Statistics Report 2013. Dublin, Ireland; 2014.
ITA	Italy	2004	2004	94.5		3	534568	2	EURO-PERISTAT Project, with SCPE, EUROCAT, EURONEOSTAT. European Perinatal Health Report-data for 2004. 2008.
ITA	Italy	2010	2010	96.6		3	522773	2	EURO-PERISTAT Project with SCPE and EUROCAT. European Perinatal Health Report. The health and

ISO3	Country name	Data coverage period start year	Data coverage period end year	Coverage of early ANC visits (%) (women 15-49 years)	No. of pregnant women (15-49 years) having early ANC visit	Timing of first visit codes: 1=<4 mo; 2=<14 weeks; 3=<15 weeks; 4=<16 weeks; 5=1st trimester; 6=<6 mo; 7<12 weeks; 8<13 weeks; 9<20 weeks; 10<3 mo	Sample size	Source code: 1=survey; 2=adm.; 3= perinatal	Sources
									care of pregnant women and babies in Europe in 2010.2013.
JAM	Jamaica	1992	1997	57.1		1	3777	1	National Family Planning Board. Reproductive Health Survey 1997 Jamaica. Final Report. Jamaica; 1999.
JAM	Jamaica	1997	2002	60.7		5	3044	1	National Family planning Board. Reproductive Health Survey Jamaica 2008: Final Report. Kingston, Jamaica; 2005.
JAM	Jamaica	2003	2009	60.6		5	2880	1	Serbanescu F, Ruiz A, Suchdev DB. Reproductive Health Survey Jamaica 2008: Final Report. Atlanta, GA (USA), Kingston, Jamaica; 2010.
JOR	Jordan	1985	1990	57.9		1	8181	1	Department of Statistics, Ministry of Health and IRD/Macro International Inc. Jordan Population and Family Health Survey 1990. Columbia, Maryland USA: IRD/Macro International Inc.; 1992.
JOR	Jordan	1992	1997	78.8		1	6360	1	Department of Statistics (DOS) [Jordan] and Macro International Inc. (MI). Jordan Population and Family Health Survey 1997. Calverton, Maryland: DOS and MI; 1998.
JOR	Jordan	1997	2002	85.1		1	3743	1	Department of Statistics [Jordan] and ORC Macro. Jordan Population and Family Health Survey 2002. Calverton, Maryland, USA: Department of Statistics and ORC Macro; 2003.
JOR	Jordan	2002	2007	89.2		1	6446	1	Department of Statistics [Jordan] and Macro International Inc. Jordan Population and Family Health Survey 2007. Calverton, Maryland, USA: Department of Statistics and Macro International Inc.; 2008.
JOR	Jordan	2007	2012	90.6		1	6577	1	Department of Statistics [Jordan] and ICF International. Jordan Population and Family Health Survey 2012. Calverton, Maryland, USA: Department of Statistics and ICF International; 2013.
KEN	Kenya	1988	1993	13.7		1	6062	1	National Council for Population and Development (NCPD), Central Bureau of Statistics (CBS) (Office of the Vice President and Ministry of Planning and National Development [Kenya]), Macro International Inc. (MI). Kenya Demographic and Health Survey 1993. Calverton, Maryland: NCPD, CBS, and MI;

ISO3	Country name	Data coverage period start year	Data coverage period end year	Coverage of early ANC visits (%) (women 15-49 years)	No. of pregnant women (15-49 years) having early ANC visit	Timing of first visit codes: 1=<4 mo; 2=<14 weeks; 3=<15 weeks; 4=<16 weeks; 5=1st trimester; 6=<6 mo; 7<12 weeks; 8<13 weeks; 9<20 weeks; 10<3 mo	Sample size	Source code: 1=survey; 2=adm.; 3= perinatal	Sources
									1994.
KEN	Kenya	1995	1998	13.4		1	3464	1	National Council for Population and Development (NCPD), Central Bureau of Statistics (CBS) (Office of the Vice President and Ministry of Planning and National Development) [Kenya], Macro International Inc. (MI). Kenya Demographic and Health Survey 1998. Calverton, Maryland: NDPD, CBS, and MI; 1999.
KEN	Kenya	1998	2003	11.1		1	4052	1	Central Bureau of Statistics (CBS) [Kenya], Ministry of Health (MOH) [Kenya], ORC Macro. Kenya Demographic and Health Survey 2003. Calverton, Maryland: CBS, MOH, and ORC Macro; 2004.
KEN	Kenya	2003	2009	14.6		1	3680	1	Kenya National Bureau of Statistics (KNBS), ICF Macro. Kenya Demographic and Health Survey 2008-09. Calverton, Maryland: KNBS and ICF Macro; 2010.
KEN	Kenya	2009	2014	19.8		1	14442	1	Kenya National Bureau of Statistics, Ministry of Health, National AIDS Control Council, Kenya Medical Research Institute, National Council for Population and Development, Nairobi, Kenya, The DHS Program. Kenya Demographic and Health Survey 2014. ICF International, Rockville, Maryland, USA; 2015.
KIR	Kiribati	2004	2009	35.7		1	787	1	Kiribati National Statistics Office (KNSO),SPC. Kiribati Demographic and Health Survey 2009 . Secretariat of the Pacific Community (SPC), Noumea; 2010.
KGZ	Kyrgyzstan	1994	1997	71.2		1	1172	1	Research Institute of Obstetrics and Pediatrics [Kyrgyz Republic], Macro International Inc. Kyrgyz Republic Demographic and Health Survey, 1997. Calverton, Maryland: Research Institute of Obstetrics and Pediatrics, Ministry of Health of the Kyrgyz Republic and Macro International Inc.; 1998.
KGZ	Kyrgyzstan	2007	2012	78.8		1	3014	1	National Statistical Committee of the Kyrgyz Republic (NSC), Ministry of Health [Kyrgyz Republic],ICF International. Kyrgyz Republic Demographic and Health Survey 2012. Bishkek,

ISO3	Country name	Data coverage period start year	Data coverage period end year	Coverage of early ANC visits (%) (women 15-49 years)	No. of pregnant women (15-49 years) having early ANC visit	Timing of first visit codes: 1=<4 mo; 2=<14 weeks; 3=<15 weeks; 4=<16 weeks; 5=1st trimester; 6=<6 mo; 7<12 weeks; 8<13 weeks; 9<20 weeks; 10<3 mo	Sample size	Source code: 1=survey; 2=adm.; 3= perinatal	Sources
									Kyrgyz Republic, Calverton, Maryland, USA: NSC, MOH, and ICF International; 2013.
KGZ	Kyrgyzstan	2012	2014	85.8		1	1675	1	National Statistical Committee of the Kyrgyz Republic, UNICEF. Kyrgyzstan Multiple Indicator Cluster Survey 2014. Final Report. Bishkek, Kyrgyzstan: National Statistical Committee of the Kyrgyz Republic, UNICEF; 2015.
LAO	Lao People's Democratic Republic	2000	2005	66.2		6	2346	1	Committee for Planning and Investment, National Statistics Centre and UNFPA. Lao Reproductive Health Survey 2005. Vientiane, Lao People's Democratic Republic; 2007.
LVA	Latvia	2014	2014	88.6		7	21244	2	Centre of Health Economics. Statistical Yearbook of Health Care in Latvia 16th edition, 2014. Riga, Latvia; 2015.
LVA	Latvia	2013	2013	91		7	20094	2	Centre of Health Economics. Statistical Yearbook of Health Care in Latvia 16th edition, 2014. Riga, Latvia; 2015.
LVA	Latvia	2012	2012	89.6		7	19401	2	Centre of Health Economics. Statistical Yearbook of Health Care in Latvia 16th edition, 2014. Riga, Latvia; 2015.
LVA	Latvia	2011	2011	88.3		7	18331	2	Centre of Health Economics. Statistical Yearbook of Health Care in Latvia 16th edition, 2014. Riga, Latvia; 2015.
LVA	Latvia	2010	2010	87.5		7	19003	2	Centre of Health Economics. Statistical Yearbook of Health Care in Latvia 16th edition, 2014. Riga, Latvia; 2015.
LVA	Latvia	2009	2009	88.4		7	21559	2	Centre of Health Economics. Statistical Yearbook of Health Care in Latvia 16th edition, 2014. Riga, Latvia; 2015.
LVA	Latvia	2008	2008	89.9		7	23834	2	Centre of Health Economics. Statistical Yearbook of Health Care in Latvia 13th edition, 2011. Riga, Latvia; 2012.
LVA	Latvia	2007	2007	87.3		7	23099	2	Centre of Health Economics. Statistical Yearbook of Health Care in Latvia 13th edition, 2011. Riga, Latvia; 2012.
LVA	Latvia	2006	2006	89.6		7	22197	2	Centre of Health Economics. Statistical Yearbook of Health Care in Latvia 13th edition, 2011. Riga, Latvia; 2012.

ISO3	Country name	Data coverage period start year	Data coverage period end year	Coverage of early ANC visits (%) (women 15-49 years)	No. of pregnant women (15-49 years) having early ANC visit	Timing of first visit codes: 1=<4 mo; 2=<14 weeks; 3=<15 weeks; 4=<16 weeks; 5=1st trimester; 6=<6 mo; 7<12 weeks; 8<13 weeks; 9<20 weeks; 10<3 mo	Sample size	Source code: 1=survey; 2=adm.; 3= perinatal	Sources
LVA	Latvia	2005	2005	89.7		7	21417	2	Centre for Disease Prevention and Control of Latvia. Statistical Yearbook of Health Care in Latvia 12th edition, 2010. Riga, Latvia; 2011.
LSO	Lesotho	1999	2004	30.3		1	2859	1	Ministry of Health and Social Welfare (MOHSW) [Lesotho], Bureau of Statistics (BOS) [Lesotho], ORC Macro. Lesotho Demographic and Health Survey 2004. Calverton, Maryland: MOH, BOS, and ORC Macro; 2005.
LSO	Lesotho	2004	2009	32.5		1	2984	1	Ministry of Health and Social Welfare (MOHSW) [Lesotho], ICF Macro. Lesotho Demographic and Health Survey 2009. Maseru, Lesotho: MOHSW and ICF Macro; 2010.
LBR	Liberia	2002	2007	58.5		1	3928	1	Liberia Institute of Statistics and Geo-Information Services (LISGIS) [Liberia], Ministry of Health and Social Welfare [Liberia], National AIDS Control Program [Liberia], Macro International Inc. Liberia Demographic and Health Survey 2007. Monrovia, Liberia: Liberia Institute of Statistics and Geo-Information Services (LISGIS), Macro International Inc.; 2008.
LBR	Liberia	2008	2013	66.6		1	4769	1	Liberia Institute of Statistics and Geo-Information Services (LISGIS), Ministry of Health and Social Welfare [Liberia], National AIDS Control Program [Liberia], ICF International. Liberia Demographic and Health Survey 2013. Monrovia, Liberia: Liberia Institute of Statistics and Geo- Information Services (LISGIS), ICF International; 2014.
LTU	Lithuania	2004	2004	74.5		3	29306	2	EURO-PERISTAT Project, with SCPE, EUROCAT, EURONEOSTAT. European Perinatal Health Report-data for 2004. 2008.
LTU	Lithuania	2010	2010	82.6		3	28406	2	EURO-PERISTAT Project with SCPE and EUROCAT. European Perinatal Health Report. The health and care of pregnant women and babies in Europe in 2010.2013.
LUX	Luxembourg	2009	2009	91.5		5	6217	1	Ministère de la Santé, CRP-Santé - Centre d'Etudes en Santé. Surveillance de la santé périnatale au Luxembourg évolution de 2001 à 2011. Luxembourg; 2013.

ISO3	Country name	Data coverage period start year	Data coverage period end year	Coverage of early ANC visits (%) (women 15-49 years)	No. of pregnant women (15-49 years) having early ANC visit	Timing of first visit codes: 1=<4 mo; 2=<14 weeks; 3=<15 weeks; 4=<16 weeks; 5=1st trimester; 6=<6 mo; 7<12 weeks; 8<13 weeks; 9<20 weeks; 10<3 mo	Sample size	Source code: 1=survey; 2=adm.; 3= perinatal	Sources
LUX	Luxembourg	2010	2010	94.9		5	6560	1	Ministère de la Santé, CRP-Santé - Centre d'Etudes en Santé. Surveillance de la santé périnatale au Luxembourg évolution de 2001 à 2011. Luxembourg; 2013.
LUX	Luxembourg	2011	2011	94		5	6351	1	Ministère de la Santé, CRP-Santé - Centre d'Etudes en Santé. Surveillance de la santé périnatale au Luxembourg évolution de 2001 à 2011. Luxembourg; 2013.
MDG	Madagascar	1987	1992	20.8		1	5604	1	Centre National de Recherches sur l'Environnement, Macro International Inc. Enquête Nationale Démographique et Sanitaire Madagascar en 1992. Calverton, Maryland USA: Macro International Inc.; 1994.
MDG	Madagascar	1994	1997	16.6		1	3893	1	Direction de la Démographie et des Statistiques Sociales, Institut National de la Statistique (INSTAT) [Madagascar] et Macro International Inc. Enquête Démographique et de Santé, Madagascar 1997. Calverton, Maryland, USA: INSTAT et Macro International Inc.; 1998.
MDG	Madagascar	1998	2004	20.9		1	4162	1	Institut National de la Statistique (INSTAT), ORC Macro. Enquête Démographique et de Santé de Madagascar 2003-2004. Calverton, Maryland, USA: INSTAT et ORC Macro; 2005.
MDG	Madagascar	2003	2009	27.4		1	8662	1	Institut National de la Statistique (INSTAT), ICF Macro. Enquête Démographique et de Santé de Madagascar 2008-2009. Antananarivo, Madagascar: INSTAT et ICF Macro; 2010.
MDG	Madagascar	2007	2013	28.7		1	8780	1	l'Institut National de la Statistique, l'Office National de Nutrition (ONN). L'Enquête Nationale sur le Suivi des indicateurs des Objectifs du Millénaire pour le Développement 2012-2013 (ENSOMD). Madagascar; 2014.
MWI	Malawi	1987	1992	8.7		1	4512	1	National Statistical Office and Macro International Inc. Malawi Demographic and Health Survey 1992. Calverton, Maryland USA: Macro International Inc.; 1994.
MWI	Malawi	1995	2000	6.5		1	8057	1	National Statistical Office [Malawi], ORC Macro. Malawi Demographic and Health Survey 2000. Zomba, Malawi and Calverton, Maryland, USA:

ISO3	Country name	Data coverage period start year	Data coverage period end year	Coverage of early ANC visits (%) (women 15-49 years)	No. of pregnant women (15-49 years) having early ANC visit	Timing of first visit codes: 1=<4 mo; 2=<14 weeks; 3=<15 weeks; 4=<16 weeks; 5=1st trimester; 6=<6 mo; 7<12 weeks; 8<13 weeks; 9<20 weeks; 10<3 mo	Sample size	Source code: 1=survey; 2=adm.; 3= perinatal	Sources
									National Statistical Office and ORC Macro; 2001.
MWI	Malawi	1999	2004	7.7		1	7271	1	National Statistical Office (NSO) [Malawi], ORC Macro. Malawi Demographic and Health Survey 2004. Calverton, Maryland, USA: NSO and ORC Macro; 2005.
MWI	Malawi	2005	2010	12.4		1	13664	1	National Statistical Office (NSO), ICF Macro. Malawi Demographic and Health Survey 2010. Zomba, Malawi, and Calverton, Maryland, USA: NSO and ICF Macro; 2011.
MWI	Malawi	2012	2014	20.8		1	7490	1	National Statistical Office. Malawi MDG Endline Survey 2014. Zomba, Malawi: National Statistical Office; 2015.
MDV	Maldives	2004	2009	90.3		1	3190	1	Ministry of Health and Family (MOHF), ICF Macro. Maldives Demographic and Health Survey 2009. Calverton, Maryland: MOHF, ICF Macro; 2010.
MLI	Mali	1992	1996	18.9		1	6019	1	Coulibaly S, Fatoumata D, Traoré SM, Sidibé O, Seroussi, Barrère MB. Enquête Démographique et de Santé, Mali 1995-1996. Calverton, Maryland, USA: Cellule de Planification et de Statistique du Ministère de la Santé), Direction Nationale de la Statistique et de l'Informatique et Macro International Inc.; 1996.
MLI	Mali	1996	2001	22.5		1	8291	1	Cellule de Planification et de Statistique du Ministère de la Santé (CPS/MS), Direction Nationale de la Statistique et de l'Informatique (DNSI), ORC Macro. Enquête Démographique et de Santé au Mali 2001. Calverton, Maryland, USA: CPS/MS, DNSI et ORC Macro; 2002.
MLI	Mali	2001	2006	30.4		1	9087	1	Cellule de Planification et de Statistique du Ministère de la Santé (CPS/MS), Direction Nationale de la Statistique et de l'Informatique du Ministère de l'Économie, de l'Industrie et du Commerce (DNSI/MEIC) et Macro International Inc. Enquête Démographique et de Santé du Mali 2006. Calverton, Maryland, USA: CPS/DNSI et Macro International Inc.; 2007.

ISO3	Country name	Data coverage period start year	Data coverage period end year	Coverage of early ANC visits (%) (women 15-49 years)	No. of pregnant women (15-49 years) having early ANC visit	Timing of first visit codes: 1=<4 mo; 2=<14 weeks; 3=<15 weeks; 4=<16 weeks; 5=1st trimester; 6=<6 mo; 7<12 weeks; 8<13 weeks; 9<20 weeks; 10<3 mo	Sample size	Source code: 1=survey; 2=adm.; 3= perinatal	Sources
MLI	Mali	2007	2013	34.5		1	6773	1	Cellule de Planification et de Statistique (CPS/SSDSPF), Institut National de la Statistique (INSTAT/MPATP), INFO-STAT et ICF International. Enquête Démographique et de Santé au Mali 2012-2013. Rockville, Maryland, USA : CPS, INSTAT, INFO-STAT et ICF International; 2014.
MLT	Malta	2004	2004	66.3		3	3838	2	EURO-PERISTAT Project, with SCPE, EUROCAT, EURONEOSTAT. European Perinatal Health Report-data for 2004. 2008.
MHL	Marshall Islands	2002	2007	42.8		1	774	1	Economic Policy, Planning and Statistics Office (EPPSO), Secretariat of the Pacific Community (SPC), Macro International Inc. Republic of the Marshall Islands Demographic and Health Survey 2007. EPPSO; Majuro, Marshall Islands; 2008.
MRT	Mauritania	1995	2001	42.9		1	3432	1	Office National de la Statistique (ONS) [Mauritanie], ORC Macro. Enquête Démographique et de Santé Mauritanie 2000-2001. Calverton, Maryland, USA: ONS et ORC Macro; 2001.
MUS	Mauritius	2004	2004	24.5		10	15505	2	Ministry of Health and Quality of Life. Statistics Report 2014. Island of Mauritius: Ministry of Health and Quality of Life; 2015.
MUS	Mauritius	2005	2005	23		10	14887	2	Ministry of Health and Quality of Life. Statistics Report 2014. Island of Mauritius: Ministry of Health and Quality of Life; 2015.
MUS	Mauritius	2006	2006	23.8		10	13714	2	Ministry of Health and Quality of Life. Statistics Report 2014. Island of Mauritius: Ministry of Health and Quality of Life; 2015.
MUS	Mauritius	2007	2007	23.9		10	13355	2	Ministry of Health and Quality of Life. Statistics Report 2014. Island of Mauritius: Ministry of Health and Quality of Life; 2015.
MUS	Mauritius	2008	2008	25.1		10	12497	2	Ministry of Health and Quality of Life. Statistics Report 2014. Island of Mauritius: Ministry of Health and Quality of Life; 2015.
MUS	Mauritius	2009	2009	25.7		10	11607	2	Ministry of Health and Quality of Life. Statistics Report 2014. Island of Mauritius: Ministry of Health and Quality of Life; 2015.
MUS	Mauritius	2010	2010	26.1		10	11161	2	Ministry of Health and Quality of Life. Statistics Report 2014. Island of Mauritius: Ministry of Health and Quality of Life; 2015.

ISO3	Country name	Data coverage period start year	Data coverage period end year	Coverage of early ANC visits (%) (women 15-49 years)	No. of pregnant women (15-49 years) having early ANC visit	Timing of first visit codes: 1=<4 mo; 2=<14 weeks; 3=<15 weeks; 4=<16 weeks; 5=1st trimester; 6=<6 mo; 7<12 weeks; 8<13 weeks; 9<20 weeks; 10<3 mo	Sample size	Source code: 1=survey; 2=adm.; 3= perinatal	Sources
MUS	Mauritius	2011	2011	25.4		10	10882	2	Ministry of Health and Quality of Life. Statistics Report 2014. Island of Mauritius: Ministry of Health and Quality of Life; 2015.
MUS	Mauritius	2012	2012	30		10	10639	2	Ministry of Health and Quality of Life. Statistics Report 2014. Island of Mauritius: Ministry of Health and Quality of Life; 2015.
MUS	Mauritius	2013	2013	30.8		10	9682	2	Ministry of Health and Quality of Life. Statistics Report 2014. Island of Mauritius: Ministry of Health and Quality of Life; 2015.
MUS	Mauritius	2014	2014	31.8		10	9304	2	Ministry of Health and Quality of Life. Statistics Report 2014. Island of Mauritius: Ministry of Health and Quality of Life; 2015.
MLT	Malta	2010	2010	66.8		3	3899	2	EURO-PERISTAT Project with SCPE and EUROCAT. European Perinatal Health Report. The health and care of pregnant women and babies in Europe in 2010. 2013.
MNG	Mongolia	1993	1998	56.4		1	3857	1	National Statistical Office of Mongolia, Ministry of Health, UNPFA. Reproductive Health Survey 1998. National Report. Ulaanbaatar, Mongolia: National Statistical Office of Mongolia; 1999.
MNG	Mongolia	1998	2003	72.4		1	3711	1	National Statistical Office of Mongolia, Ministry of Health, UNPFA. Mongolia Reproductive Health Survey 2003. Ulaanbaatar, Mongolia: National Statistical Office of Mongolia: 2004.
MNG	Mongolia	2003	2008	55.2		1	609	1	National Statistical Office of Mongolia, Ministry of Health, UNPFA. Mongolia Reproductive Health Survey 2008. Ulaanbaatar, Mongolia: National Statistical Office of Mongolia; 2009.
MNG	Mongolia	2008	2010	77.2		1	1654	1	National Statistics Office, UNICEF. Multiple Indicator Cluster Survey 2010, Final Report. Ulaanbaatar, Mongolia; 2013.
MNG	Mongolia	1990	1990	53.4	38729	5	72527	2	Ministry of Health. Report on Measurement of Maternal Deaths and other MDG Targets in Mongolia. Mongolia; 2013.
MNG	Mongolia	1991	1991	54.5	36727	5	67390	2	Ministry of Health. Report on Measurement of Maternal Deaths and other MDG Targets in Mongolia. Mongolia; 2013.

ISO3	Country name	Data coverage period start year	Data coverage period end year	Coverage of early ANC visits (%) (women 15-49 years)	No. of pregnant women (15-49 years) having early ANC visit	Timing of first visit codes: 1=<4 mo; 2=<14 weeks; 3=<15 weeks; 4=<16 weeks; 5=1st trimester; 6=<6 mo; 7<12 weeks; 8<13 weeks; 9<20 weeks; 10<3 mo	Sample size	Source code: 1=survey; 2=adm.; 3= perinatal	Sources
MNG	Mongolia	1992	1992	63.2	35807	5	56658	2	Ministry of Health. Report on Measurement of Maternal Deaths and other MDG Targets in Mongolia. Mongolia; 2013.
MNG	Mongolia	1993	1993	67.2	30543	5	45451	2	Ministry of Health. Report on Measurement of Maternal Deaths and other MDG Targets in Mongolia. Mongolia; 2013.
MNG	Mongolia	1994	1994	59.6	23259	5	39019	2	Ministry of Health. Report on Measurement of Maternal Deaths and other MDG Targets in Mongolia. Mongolia; 2013.
MNG	Mongolia	1995	1995	60.3	23819	5	39501	2	Ministry of Health. Report on Measurement of Maternal Deaths and other MDG Targets in Mongolia. Mongolia; 2013.
MNG	Mongolia	1996	1996	64.2	28708	5	44717	2	Ministry of Health. Report on Measurement of Maternal Deaths and other MDG Targets in Mongolia. Mongolia; 2013.
MNG	Mongolia	1997	1997	61.5	36255	5	58920	2	Ministry of Health. Report on Measurement of Maternal Deaths and other MDG Targets in Mongolia. Mongolia; 2013.
MNG	Mongolia	1998	1998	62.7	36500	5	58208	2	Ministry of Health. Report on Measurement of Maternal Deaths and other MDG Targets in Mongolia. Mongolia; 2013.
MNG	Mongolia	1999	1999	65.2	39608	5	60789	2	Ministry of Health. Report on Measurement of Maternal Deaths and other MDG Targets in Mongolia. Mongolia; 2013.
MNG	Mongolia	2000	2000	65.7	38159	5	58070	2	Ministry of Health. Report on Measurement of Maternal Deaths and other MDG Targets in Mongolia. Mongolia; 2013.
MNG	Mongolia	2001	2001	67.9	38625	5	56880	2	Ministry of Health. Report on Measurement of Maternal Deaths and other MDG Targets in Mongolia. Mongolia; 2013.
MNG	Mongolia	2002	2002	71.9	39381	5	54807	2	Ministry of Health. Report on Measurement of Maternal Deaths and other MDG Targets in Mongolia. Mongolia; 2013.
MNG	Mongolia	2003	2003	73.8	35275	5	47793	2	Ministry of Health. Report on Measurement of Maternal Deaths and other MDG Targets in Mongolia. Mongolia; 2013.
MNG	Mongolia	2004	2004	77.6	37851	5	48794	2	Ministry of Health. Report on Measurement of Maternal Deaths and other MDG Targets in Mongolia. Mongolia; 2013.

ISO3	Country name	Data coverage period start year	Data coverage period end year	Coverage of early ANC visits (%) (women 15-49 years)	No. of pregnant women (15-49 years) having early ANC visit	Timing of first visit codes: 1=<4 mo; 2=<14 weeks; 3=<15 weeks; 4=<16 weeks; 5=1st trimester; 6=<6 mo; 7<12 weeks; 8<13 weeks; 9<20 weeks; 10<3 mo	Sample size	Source code: 1=survey; 2=adm.; 3= perinatal	Sources
MNG	Mongolia	2005	2005	80	40063	5	50099	2	Ministry of Health. Report on Measurement of Maternal Deaths and other MDG Targets in Mongolia. Mongolia; 2013.
MNG	Mongolia	2006	2006	81.5	42380	5	52015	2	Ministry of Health. Report on Measurement of Maternal Deaths and other MDG Targets in Mongolia. Mongolia; 2013.
MNG	Mongolia	2007	2007	84	55908	5	66595	2	Ministry of Health. Report on Measurement of Maternal Deaths and other MDG Targets in Mongolia. Mongolia; 2013.
MNG	Mongolia	2008	2008	83.7	59600	5	71175	2	Ministry of Health. Report on Measurement of Maternal Deaths and other MDG Targets in Mongolia. Mongolia; 2013.
MNG	Mongolia	2009	2009	83.2	59592	5	71598	2	Ministry of Health. Report on Measurement of Maternal Deaths and other MDG Targets in Mongolia. Mongolia; 2013.
MNG	Mongolia	2010	2010	83.4	58821	5	70538	2	Ministry of Health. Report on Measurement of Maternal Deaths and other MDG Targets in Mongolia. Mongolia; 2013.
MNG	Mongolia	2011	2011	85.5	65676	5	76826	2	Ministry of Health. Report on Measurement of Maternal Deaths and other MDG Targets in Mongolia. Mongolia; 2013.
MNG	Mongolia	2012	2012	87.5	71114	5	81292	2	Ministry of Health. Report on Measurement of Maternal Deaths and other MDG Targets in Mongolia. Mongolia; 2013.
MAR	Morocco	1997	1992	23.9		1	5138	1	Ministère de la Santé Publique, Macro International Inc. Enquête Nationale sur la Population et la Santé (ENPS-II) 1992. Columbia, Maryland, USA: Macro International Inc.;1993.
MAR	Morocco	1998	2004	50.9		1	4695	1	Ministère de la Santé [Maroc], ORC Macro, et Ligue des États Arabes. Enquête sur la Population et la Santé Familiale (EPSF) 2003-2004. Calverton, Maryland, USA: Ministère de la Santé et ORC Macro; 2005.
MAR	Morocco	2006	2011	69.9		1	5648	1	Ministère de la Santé (MS). Enquête Nationale sur la Population et la Santé Familiale (ENPSF) 2011 .Maroc; 2012.
MOZ	Mozambique	1994	1997	17.6		1	4207	1	Instituto Nacional de Estatística, Macro International Inc. Moçambique Inquérito Demográfico e de Saúde 1997. Calverton, Maryland, USA: Macro

ISO3	Country name	Data coverage period start year	Data coverage period end year	Coverage of early ANC visits (%) (women 15-49 years)	No. of pregnant women (15-49 years) having early ANC visit	Timing of first visit codes: 1=<4 mo; 2=<14 weeks; 3=<15 weeks; 4=<16 weeks; 5=1st trimester; 6=<6 mo; 7<12 weeks; 8<13 weeks; 9<20 weeks; 10<3 mo	Sample size	Source code: 1=survey; 2=adm.; 3= perinatal	Sources
									International Inc.; 1998.
MOZ	Mozambique	1998	2003	17.8		1	7179	1	Instituto Nacional de Estatística, Ministério da Saúde, ORC Macro. Mozambique Inquérito Demográfico e de Saúde 2003. Ministério da Saúde. ORC Macro. Calverton MD, USA: ORC Macro; 2005.
MOZ	Mozambique	2006	2011	13.1		1	7874	1	Ministerio da Saude (MISAU), Instituto Nacional de Estatística (INE), ICF International (ICFI). Mozambique Inquérito Demográfico e de Saúde 2011. Calverton, Maryland, USA: MISAU, INE e ICFI; 2013.
MMR	Myanmar	1996	2001	70.1		6	4632	1	Ministry of Immigration and Population, Department of Population, UNPFA. Myanmar Fertility and Reproductive Health Survey 2001. Detailed Analysis Report. Yangon, Myanmar; 2004.
NAM	Namibia	1987	1992	26.9		1	3814	1	Ministry of Health and Social Services, Macro International Inc. Namibia Demographic and Health Survey 1992. Columbia, Maryland, USA: Macro International Inc.; 1993.
NAM	Namibia	1995	2000	31		1	3002	1	Ministry of Health and Social Services (MOHSS) [Namibia]. Namibia Demographic and Health Survey 2000. Windhoek, Namibia: MOHSS; 2003.
NAM	Namibia	2001	2007	32.6		1	3898	1	Ministry of Health and Social Services (MoHSS) [Namibia], Macro International Inc. Namibia Demographic and Health Survey 2006-07. Windhoek, Namibia and Calverton, Maryland, USA: MoHSS and Macro International Inc.; 2008.
NAM	Namibia	2008	2013	42.5		1	3842	1	The Namibia Ministry of Health and Social Services (MoHSS), ICF International. The Namibia Demographic and Health Survey 2013. Windhoek, Namibia, and Rockville, Maryland, USA: MoHSS and ICF International; 2014.
NRU	Nauru	2002	2007	16.8		1	205	1	Nauru Bureau of Statistics, SPC, Macro International Inc. Nauru 2007 Demographic and Health Survey. Noumea, New Caledonia, SPC; 2009.
NPL	Nepal	1993	1996	13.1		1	4375	1	Family Health Division, Ministry of Health, New ERA, Macro International Inc. Nepal Family Health Survey 1996. Calverton, Maryland, USA: Macro

ISO3	Country name	Data coverage period start year	Data coverage period end year	Coverage of early ANC visits (%) (women 15-49 years)	No. of pregnant women (15-49 years) having early ANC visit	Timing of first visit codes: 1=<4 mo; 2=<14 weeks; 3=<15 weeks; 4=<16 weeks; 5=1st trimester; 6=<6 mo; 7<12 weeks; 8<13 weeks; 9<20 weeks; 10<3 mo	Sample size	Source code: 1=survey; 2=adm.; 3= perinatal	Sources
									International Inc.; 1997.
NPL	Nepal	1996	2001	16.4		1	4745	1	Ministry of Health [Nepal], New ERA, ORC Macro. Nepal Demographic and Health Survey 2001. Calverton, Maryland, USA: Family Health Division, Ministry of Health; New ERA; and ORC Macro; 2002.
NPL	Nepal	2001	2006	27.7		1	4066	1	Ministry of Health and Population (MOHP) [Nepal], New ERA, Macro International Inc. Nepal Demographic and Health Survey 2006. Kathmandu, Nepal: Ministry of Health and Population, New ERA, and Macro International Inc.; 2007.
NPL	Nepal	2006	2011	49.7		1	4148	1	Ministry of Health and Population (MOHP) [Nepal], New ERA, ICF International Inc. Nepal Demographic and Health Survey 2011. Kathmandu, Nepal: Ministry of Health and Population, New ERA, and ICF International, Calverton, Maryland; 2012.
NPL	Nepal	2012	2014	50.8		1	2048	1	Government of Nepal, Central Bureau of Statistics, the United Nations Children's Fund (UNICEF). Multiple Indicator Cluster Survey 2014. Key Finding and Tables. Nepal, Central Bureau of Statistics; 2015.
NLD	Netherlands	2010	2010	87.3		3	161722	2	EURO-PERISTAT Project with SCPE and EUROCAT. European Perinatal Health Report. The health and care of pregnant women and babies in Europe in 2010.2013.
NZL	New Zealand	2008	2008	41		5	64623	2	Ministry of Health (MoH). Maternity Tables 2013. Wellington, New Zealand: MOH; 2015.
NZL	New Zealand	2009	2009	47.2		5	64233	2	Ministry of Health (MoH). Maternity Tables 2013. Wellington, New Zealand: MOH; 2015.
NZL	New Zealand	2010	2010	50		5	64462	2	Ministry of Health (MoH). Maternity Tables 2013. Wellington, New Zealand: MOH; 2015.
NZL	New Zealand	2011	2011	53.9		5	62309	2	Ministry of Health (MoH). Maternity Tables 2013. Wellington, New Zealand: MOH; 2015.

ISO3	Country name	Data coverage period start year	Data coverage period end year	Coverage of early ANC visits (%) (women 15-49 years)	No. of pregnant women (15-49 years) having early ANC visit	Timing of first visit codes: 1=<4 mo; 2=<14 weeks; 3=<15 weeks; 4=<16 weeks; 5=1st trimester; 6=<6 mo; 7<12 weeks; 8<13 weeks; 9<20 weeks; 10<3 mo	Sample size	Source code: 1=survey; 2=adm.; 3= perinatal	Sources
NZL	New Zealand	2012	2012	56.1		5	62333	2	Ministry of Health (MoH). Maternity Tables 2013. Wellington, New Zealand: MOH; 2015.
NZL	New Zealand	2013	2013	58.4		5	59227	2	Ministry of Health (MoH). Maternity Tables 2013. Wellington, New Zealand: MOH; 2015.
NZL	New Zealand	2014	2014	61.8		5	59193	2	Ministry of Health (MoH). Report on Maternity 2014. Wellington, New Zealand: MOH; 2015.
NIC	Nicaragua	1987	1993	36.7		5	5469	1	Asociación Pro-Bienestar de la Familia Nicaragüense - PROFAMILIA, CDC. Encuesta sobre Salud Familiar Nicaragua 92-93. Managua, Nicaragua; 1993.
NIC	Nicaragua	1993	1998	61.3		1	7992	1	Instituto Nacional de Estadísticas y Censos (INEC), Ministerio de Salud (MINSA), Macro International Inc. Encuesta Nicaragüense de Demografía y Salud 1998. Calverton, Maryland, USA: Macro International Inc.; 1999.
NIC	Nicaragua	1996	2001	61		1	4848	1	Instituto Nacional de Estadísticas y Censos (INEC), Ministerio de Salud (MINSA), ORC Macro. Encuesta Nicaragüense de Demografía y Salud 2001. Calverton, USA: ORC Macro; 2002.
NIC	Nicaragua	2001	2007	70.2		1	7228	1	Instituto Nacional de Información de Desarrollo (INIDE), Ministerio de Salud (MINSA). Encuesta Nicaragüense de Demografía y Salud ENDESA 2006/07. Informe Final. Nicaragua; 2008.
NIC	Nicaragua	2006	2012	77.1		1	6774	1	Instituto Nacional de Información de Desarrollo (INIDE), Ministerio de Salud (MINSA). Encuesta Nicaragüense de Demografía y Salud 2011/12. Informe Preliminar. Ministerio de Salud, Nicaragua; 2013.
NER	Niger	1987	1992	11.2		1	7094	1	Direction de la Statistique et des Comptes Nationaux, Direction Général du Plan, Ministère des Finances et du Plan, Macro International Inc. Enquête Démographique et de Santé Niger 1992. Columbia, Maryland, USA: Macro International Inc.;1993.
NER	Niger	1995	1998	13.5		1	5007	1	Attama S, Seroussi M, Kourguéni AI, Koché H, Barrèm B. Enquête Démographique et de Santé, Niger 1998. Calverton, Maryland, USA: Care

ISO3	Country name	Data coverage period start year	Data coverage period end year	Coverage of early ANC visits (%) (women 15-49 years)	No. of pregnant women (15-49 years) having early ANC visit	Timing of first visit codes: 1=<4 mo; 2=<14 weeks; 3=<15 weeks; 4=<16 weeks; 5=1st trimester; 6=<6 mo; 7<12 weeks; 8<13 weeks; 9<20 weeks; 10<3 mo	Sample size	Source code: 1=survey; 2=adm.; 3= perinatal	Sources
									International Niger et Macro International Inc.; 1999.
NER	Niger	2001	2006	13.9		1	6301	1	Institut National de la Statistique (INS), Macro International Inc. Enquête Démographique et de Santé et à Indicateurs Multiples du Niger 2006. Calverton, Maryland, USA: INS et Macro International Inc.; 2007.
NER	Niger	2007	2012	22.1		1	8002	1	Institut National de la Statistique (INS), ICF International. Enquête Démographique et de Santé et à Indicateurs Multiples du Niger 2012. Calverton, Maryland, USA : INS et ICF International; 2013.
NGA	Nigeria	1985	1990	12.9		1	8113	1	Federal Office of Statistics, IRD/Macro International Inc. Nigeria Demographic and Health Survey 1990. Colombia, Maryland, USA: IRD/Macro International Inc.; 1992.
NGA	Nigeria	1996	1999	15.5		1	3547	1	National Population Commission [Nigeria]. Nigeria Demographic and Health Survey 1999. Calverton, Maryland: National Population Commission and ORC/Macro; 2000.
NGA	Nigeria	1998	2003	16.7		1	3911	1	National Population Commission (NPC) [Nigeria], ORC Macro. Nigeria Demographic and Health Survey 2003. Calverton, Maryland: National Population Commission and ORC Macro; 2004.
NGA	Nigeria	2003	2008	16.2		1	17635	1	National Population Commission (NPC) [Nigeria], IF Macro. Nigeria Demographic and Health Survey 2008. Abuja, Nigeria: National Population Commission and ICF Macro; 2009.
NGA	Nigeria	2008	2013	17.6		1	20467	1	National Population Commission (NPC) [Nigeria], ICF International. Nigeria Demographic and Health Survey 2013. Abuja, Nigeria, and Rockville, Maryland, USA: NPC and ICF International; 2014.
OMN	Oman	2000	2000	57.6		5	39994	2	Ministry of Health. Department of Health Information and Statistics, Directorate General of Planning. Annual Health Report 2011. Oman; 2012.
OMN	Oman	2005	2005	65.5		5	42065	2	Ministry of Health. Department of Health Information and Statistics, Directorate General of Planning. Annual Health Report 2011. Oman; 2012.

ISO3	Country name	Data coverage period start year	Data coverage period end year	Coverage of early ANC visits (%) (women 15-49 years)	No. of pregnant women (15-49 years) having early ANC visit	Timing of first visit codes: 1=<4 mo; 2=<14 weeks; 3=<15 weeks; 4=<16 weeks; 5=1st trimester; 6=<6 mo; 7<12 weeks; 8<13 weeks; 9<20 weeks; 10<3 mo	Sample size	Source code: 1=survey; 2=adm.; 3= perinatal	Sources
OMN	Oman	2006	2006	64.2		5	44116	2	Ministry of Health. Department of Health Information and Statistics, Directorate General of Planning. Annual Health Report 2011. Oman; 2012.
OMN	Oman	2007	2007	64.8		5	46609	2	Ministry of Health. Department of Health Information and Statistics, Directorate General of Planning. Annual Health Report 2011. Oman; 2012.
OMN	Oman	2008	2008	64.5		5	50614	2	Ministry of Health. Department of Health Information and Statistics, Directorate General of Planning. Annual Health Report 2011. Oman; 2012.
OMN	Oman	2009	2009	64.2		5	55950	2	Ministry of Health. Department of Health Information and Statistics, Directorate General of Planning. Annual Health Report 2011. Oman; 2012.
OMN	Oman	2010	2010	64.9		5	55712	2	Ministry of Health. Department of Health Information and Statistics, Directorate General of Planning. Annual Health Report 2011. Oman; 2012.
OMN	Oman	2011	2011	65.6		5	57323	2	Ministry of Health. Department of Health Information and Statistics, Directorate General of Planning. Annual Health Report 2011. Oman; 2012.
OMN	Oman	2012	2012	66.5		5	61549	2	Ministry of Health. Department of Health Information and Statistics, Directorate General of Planning. Annual Health Report 2014. Oman; 2015.
OMN	Oman	2013	2013	64.7		5	66772	2	Ministry of Health. Department of Health Information and Statistics, Directorate General of Planning. Annual Health Report 2014. Oman; 2015.
OMN	Oman	2014	2014	63.9		5	68293	2	Ministry of Health. Department of Health Information and Statistics, Directorate General of Planning. Annual Health Report 2014. Oman; 2015.
PAK	Pakistan	1985	1991	14.8		1	6407	1	National Institute of Population Studies, IRD/Macro International Inc. Pakistan Demographic Health survey 1990/1991. Columbia, Maryland, USA: IRD/Macro International Inc.; 1992.
PAK	Pakistan	2001	2007	30.6		1	5677	1	National Institute of Population Studies (NIPS) [Pakistan], Macro International Inc. Pakistan Demographic and Health Survey 2006-07. Islamabad, Pakistan: National Institute of Population Studies and Macro International Inc.;2008.
PAK	Pakistan	2007	2013	42.4		1	7446	1	National Institute of Population Studies (NIPS) [Pakistan], ICF International. Pakistan Demographic and Health Survey 2012-13. Islamabad, Pakistan, and

ISO3	Country name	Data coverage period start year	Data coverage period end year	Coverage of early ANC visits (%) (women 15-49 years)	No. of pregnant women (15-49 years) having early ANC visit	Timing of first visit codes: 1=<4 mo; 2=<14 weeks; 3=<15 weeks; 4=<16 weeks; 5=1st trimester; 6=<6 mo; 7<12 weeks; 8<13 weeks; 9<20 weeks; 10<3 mo	Sample size	Source code: 1=survey; 2=adm.; 3= perinatal	Sources
									Calverton, Maryland, USA: NIPS and ICF International; 2013.
PLW	Palau	2007	2007	33		5	279	2	Ministry of Health. National Health Profile 2013. Republic of Palau; 2014.
PLW	Palau	2008	2008	42		5	295	2	Ministry of Health. National Health Profile 2013. Republic of Palau; 2014.
PLW	Palau	2009	2009	44		5	273	2	Ministry of Health. National Health Profile 2013. Republic of Palau; 2014.
PLW	Palau	2010	2010	36		5	247	2	Ministry of Health. National Health Profile 2013. Republic of Palau; 2014.
PLW	Palau	2011	2011	47		5	247	2	Ministry of Health. National Health Profile 2013. Republic of Palau; 2014.
PAN	Panama	2004	2009	77.1		1	238393	1	Ruth Graciela De León Richardson et al.: Instituto Conmemorativo Gorgas de Estudios de la Salud. Panamá Encuesta Nacional de Salud Sexual y Reproductiva 2009 (ENASSER 2009)- Informe Final. Panamá; 2011.
PRY	Paraguay	1985	1990	59.2		1	3944	1	Centro Paraguayo de Estudios de Población, Macro Sistemas Inc. Encuesta Nacional de Demografía y Salud 1990. Columbia, Maryland, USA: Macro Systems Inc.; 1991.
PRY	Paraguay	1990	1996	58.1		5	4917	1	Centro Paraguay de Estudios de Poblacíon (CEPEP), Centers for Disease Control and Prevention (CDC), United States Agency for International Development (USAID). Encuesta Nacional de Demografía y Salud Reproductiva 1995-1996, ENDSR-95/96. Asunción, Paraguay; 1997.
PRY	Paraguay	1995	1998	34.8		5	1538	1	Centro Paraguay de Estudios de Poblacíon (CEPEP), Centers for Disease Control and Prevention (CDC), United States Agency for International Development (USAID). Encuesta Nacional de Salud Materno Infantil: 1998 (ENSMI-98). Informe Final. Asunción, Paraguay; 1999.

ISO3	Country name	Data coverage period start year	Data coverage period end year	Coverage of early ANC visits (%) (women 15-49 years)	No. of pregnant women (15-49 years) having early ANC visit	Timing of first visit codes: 1=<4 mo; 2=<14 weeks; 3=<15 weeks; 4=<16 weeks; 5=1st trimester; 6=<6 mo; 7<12 weeks; 8<13 weeks; 9<20 weeks; 10<3 mo	Sample size	Source code: 1=survey; 2=adm.; 3= perinatal	Sources
PRY	Paraguay	1999	2004	68.6		5	4022	1	Centro Paraguay de Estudios de Población (CEPEP), USAID, UNPFA, CDC, IPPF. Encuesta Nacional de Demografía y Salud Reproductiva 2004 (ENDSSR 2004). Informe Final. Asunción, Paraguay; 2005.
PRY	Paraguay	2003	2008	78.8		5	3057	1	Centro Paraguay de Estudios de Población (CEPEP), USAID, UNPFA, CDC, IPPF. Encuesta Nacional de Demografía y Salud Reproductiva 2008 (ENDSSR 2008). Informe Final. Asunción, Paraguay; 2009.
PER	Peru	1986	1992	46.3		1	8473	1	Nacional de Estadística e Informática (INEI), Asociación Benéfica PRISMA, Macro International Inc. Encuesta Demográfica y de Salud Familiar (ENDES 1991-1992). Columbia, Maryland, USA: Macro International Inc.; 1992
PER	Peru	1991	1996	46.3		1	15639	1	Instituto Nacional de Estadística e Informática, Macro International Inc. Peru Encuesta Demográfica y de Salud Familiar 1996. Calverton, Maryland, USA: Macro International Inc.; 1997.
PER	Peru	1995	2000	58		1	9535	1	Instituto Nacional de Estadística e Informática, Macro International Inc. Peru Encuesta Demográfica y de Salud Familiar 2000. Lima, Peru; 2001.
PER	Peru	1999	2006	69.7		1	5223	1	Instituto Nacional de Estadística e Informática, ORC Macro. Peru Encuesta Demográfica y de Salud Familiar - ENDES Continua, 2004-2006. Calverton, MD, USA: ORC Macro; 2007.
PER	Peru	2002	2008	72		1	6953	1	Instituto Nacional de Estadística e Informática, ORC Macro. Peru Encuesta Demográfica y de Salud Familiar 2007-2008 -Informe Principal. Calverton, MD, USA: ORC Macro; 2009.
PER	Peru	2004	2009	72.4		1	7737	1	Instituto Nacional de Estadística e Informática, ORC Macro. Peru Encuesta Demográfica y de Salud Familiar - ENDES Continua, 2009 Calverton, MD, USA: ORC Macro; 2010.
PER	Peru	2005	2010	73		1	7051	1	Instituto Nacional de Estadística e Informática, ORC Macro. Peru Encuesta Demográfica y de Salud Familiar - ENDES Continua, 2010 Calverton, MD, USA: ORC Macro; 2011.
PER	Peru	2006	2011	74.9		1	7053	1	Instituto Nacional de Estadística e Informática, ORC Macro. Peru Encuesta Demográfica y de Salud Familiar 2011. Calverton, MD, USA: ORC Macro;

ISO3	Country name	Data coverage period start year	Data coverage period end year	Coverage of early ANC visits (%) (women 15-49 years)	No. of pregnant women (15-49 years) having early ANC visit	Timing of first visit codes: 1=<4 mo; 2=<14 weeks; 3=<15 weeks; 4=<16 weeks; 5=1st trimester; 6=<6 mo; 7<12 weeks; 8<13 weeks; 9<20 weeks; 10<3 mo	Sample size	Source code: 1=survey; 2=adm.; 3= perinatal	Sources
									2012.
PER	Peru	2007	2012	75		1	7404	1	Instituto Nacional de Estadística e Informática, ORC Macro. Peru Encuesta Demográfica y de Salud Familiar 2012. Calverton, MD, USA: ORC Macro; 2013.
PER	Peru	2008	2013	77.4		1	6947	1	Instituto Nacional de Estadística e Informática, ORC Macro. Peru Encuesta Demográfica y de Salud Familiar 2014. Calverton, MD, USA: ORC Macro; 2015.
PER	Peru	2009	2014	77.3		1	7703	1	Instituto Nacional de Estadística e Informática, ORC Macro. Peru Encuesta Demográfica y de Salud Familiar 2013. Calverton, MD, USA: ORC Macro; 2014.
PHL	Philippines	1990	1993	48		1	4010	1	National Statistics Office (NSO), Macro International Inc. (MI). National Safe Motherhood Survey 1993. Calverton, Maryland: NSO, MI; 1994.
PHL	Philippines	1988	1993	43.3		1	8803	1	National Statistics Office (NSO) [Philippines], Macro International Inc. (MI). National Demographic Survey 1993. Calverton, Maryland: NSO and MI; 1994.
PHL	Philippines	1993	1998	51		1	7566	1	National Statistics Office (NSO)], Department of Health (DOH) [Philippines], Macro International Inc. (MI). National Demographic and Health Survey 1998. Manila: NSO and MI; 1999.
PHL	Philippines	1998	2003	53		1	4802	1	National Statistics Office (NSO) [Philippines], ORC Macro. National Demographic and Health Survey 2003. Calverton, Maryland: NSO and ORC Macro; 2004.
PHL	Philippines	2003	2008	54		1	4590	1	National Statistics Office (NSO) [Philippines], ICF Macro. National Demographic and Health Survey 2008. Calverton, Maryland: National Statistics Office and ICF Macro; 2009.
PHL	Philippines	2006	2011	57.9		1	7248	1	The National Statistics Office (NSO). 2011 Family Health Survey (FHS). NSO: Manila, Philippines; 2012.

ISO3	Country name	Data coverage period start year	Data coverage period end year	Coverage of early ANC visits (%) (women 15-49 years)	No. of pregnant women (15-49 years) having early ANC visit	Timing of first visit codes: 1=<4 mo; 2=<14 weeks; 3=<15 weeks; 4=<16 weeks; 5=1st trimester; 6=<6 mo; 7<12 weeks; 8<13 weeks; 9<20 weeks; 10<3 mo	Sample size	Source code: 1=survey; 2=adm.; 3= perinatal	Sources
PHL	Philippines	2007	2013	62.3		1	5188	1	Philippine Statistics Authority (PSA) [Philippines], ICF International. Philippines National Demographic and Health Survey 2013. Manila, Philippines, and Rockville, Maryland, USA: PSA and ICF International; 2014.
PRT	Portugal	2004	2004	91.2		3	5274	2	EURO-PERISTAT Project, with SCPE, EUROCAT, EURONEOSTAT. European Perinatal Health Report-data for 2004. 2008.
KOR	Republic of Korea	2003	2003	97.2		8	1098	1	Korea Institute for Health and Social Affairs. The 2003 National Survey on Fertility, Family Health and Welfare in Korea. Republic of Korea; 2004.
KOR	Republic of Korea	2006	2006	98.6		8	952	1	Korea Institute for Health and Social Affairs. The 2012 National Survey on Fertility, Family Health and Welfare in Korea. Republic of Korea; 2012.
KOR	Republic of Korea	2009	2009	96.9		8	975	1	Korea Institute for Health and Social Affairs. The 2012 National Survey on Fertility, Family Health and Welfare in Korea. Republic of Korea; 2012.
KOR	Republic of Korea	2012	2012	98		8	943	1	Korea Institute for Health and Social Affairs. The 2012 National Survey on Fertility, Family Health and Welfare in Korea. Republic of Korea; 2012.
MDA	Republic of Moldova	1992	1997	72.9		1	2141	1	Moldovan Ministry of Health, Division of Reproductive Health, Centers for Disease Control and Prevention. Reproductive Health Survey Moldova 1997. Final Report. Atlanta, Georgia, USA: Centers for Disease Control and Prevention; 1998.
MDA	Republic of Moldova	2000	2005	71.7		1	1387	1	National Scientific and Applied Center for Preventive Medicine (NCPM) [Moldova] and ORC Macro. Moldova Demographic and Health Survey 2005. Calverton, Maryland: National Scientific and Applied Center for Preventive Medicine of the Ministry of Health and Social Protection and ORC Macro; 2006.
ROU	Romania	1988	1993	57.3		5	1440	1	Institute for Mother and Child Care, Ministry of Health, Centers for Disease Control (CDC) and Prevention. Reproductive Health Survey Romania 1993. Atlanta, Georgia, USA: CDC; 1995.
ROU	Romania	1994	1999	60.3		5	2040	1	Romanian Association of Public Health and Health Management (ARSPMS), Centers for Disease Control and Prevention (DRH/CDC). Reproductive Health Survey Romania, 1999. Final Rapport.

ISO3	Country name	Data coverage period start year	Data coverage period end year	Coverage of early ANC visits (%) (women 15-49 years)	No. of pregnant women (15-49 years) having early ANC visit	Timing of first visit codes: 1=<4 mo; 2=<14 weeks; 3=<15 weeks; 4=<16 weeks; 5=1st trimester; 6=<6 mo; 7<12 weeks; 8<13 weeks; 9<20 weeks; 10<3 mo	Sample size	Source code: 1=survey; 2=adm.; 3= perinatal	Sources
									Atlanta, Georgia, USA: DRH/CDC; 2001.
ROU	Romania	1999	2004	74.4		5	1122	1	Ministry of Health, World Bank, UNPFA, USAID, UNICEF. Reproductive Health Survey: Romania, 2004. Summary Report, May 2005. Romania, Ministry of Health; 2005.
ROU	Romania	2010	2010	62.5		5	43584	2	EURO-PERISTAT Project, with SCPE, EUROCAT, EURONEOSTAT. European Perinatal Health Report-data for 2004. 2008.
RUS	Russian Federation	2006	2011	89.8		5	3292	1	Federal State Statistic Service (ROSSTAT), Ministry of Health of the Russian Federation, United Nations Population Fund (UNFPA), Centers for Disease Control and Prevention, Atlanta, USA (DRH/CDC). Russia Reproductive Health Survey 2011. Summary Report. Moscow: United Nations Fund for Population; 2012.
RWA	Rwanda	1987	1992	3.4		1	5612	1	Office National de la Population, Macro International Inc. Enquête Démographic et de Santé 1992. Calverton, Maryland, USA: Macro International Inc.; 1994.
RWA	Rwanda	1995	2000	4.7		1	5141	1	Office National de la Population (ONAPO) [Rwanda], ORC Macro. Enquête Démographique et de Santé, Rwanda 2000. Kigali, Rwanda et Calverton, Maryland, USA: Ministère de la Santé, Office National de la Population et ORC Macro; 2001.
RWA	Rwanda	2000	2005	7.9		1	5425	1	Institut National de la Statistique du Rwanda (INSR), ORC Macro. Rwanda Demographic and Health Survey 2005. Calverton, Maryland, USA: INSR and ORC Macro; 2006.
RWA	Rwanda	2002	2008	22		1	3658	1	Ministry of Health (MOH) [Rwanda], National Institute of Statistics of Rwanda (NISR), ICF Macro. Rwanda Interim Demographic and Health Survey 2007-08. Calverton, Maryland, USA: MOH, NISR, and ICF Macro; 2009.
RWA	Rwanda	2005	2010	38.2		1	6405	1	National Institute of Statistics of Rwanda (NISR) [Rwanda], Ministry of Health (MOH) [Rwanda], ICF International. Rwanda Demographic and Health Survey 2010. Calverton, Maryland, USA: NISR,

ISO3	Country name	Data coverage period start year	Data coverage period end year	Coverage of early ANC visits (%) (women 15-49 years)	No. of pregnant women (15-49 years) having early ANC visit	Timing of first visit codes: 1=<4 mo; 2=<14 weeks; 3=<15 weeks; 4=<16 weeks; 5=1st trimester; 6=<6 mo; 7<12 weeks; 8<13 weeks; 9<20 weeks; 10<3 mo	Sample size	Source code: 1=survey; 2=adm.; 3= perinatal	Sources
									MOH, and ICF International; 1012.
WSM	Samoa	2004	2009	13.2		1	1105	1	Ministry of Health [Samoa], Bureau of Statistics [Samoa], ICF Macro. Samoa Demographic and Health Survey 2009. Apia, Samoa: Ministry of Health, Samoa, 2010.
WSM	Samoa	2009	2014	12.1		1	2014	1	Samoa Bureau of Statistics, Ministry of Health. Samoa Demographic and Health Survey 2014. Samoa Bureau of Statistics, Government of Samoa, Apia, Samoa, 2015.
STP	Sao Tome and Principe	2003	2009	50.5		1	1386	1	Instituto Nacional de Estatística (INE) [São Tomé e Príncipe], Ministério da Saúde, ICF Macro. Inquérito Demográfico e Sanitário, São Tomé e Príncipe, IDS STP, 2008-2009. Calverton, Maryland, USA: INE; 2010.
STP	Sao Tome and Principe	2012	2014	67		5	724	1	National Institute of Statistics. Sao Tome and Principe Multiple Indicator Cluster Survey 2014, Final Report. São Tomé, Sao Tome and Principe; 2016.
SAU	Saudi Arabia	1986	1991	39.6		1	1050	2	Ministry of Health. Saudi Maternal and Child Health Survey 1991. Saudi Arabia; 1992.
SEN	Senegal	1987	1993	38.4		1	5581	1	Ministère de l'Economie des Finances et du Plan, Macro International Inc. Enquête Démographic et de Santé au Sénégal (EDS-II) 1992/93. Calverton, Maryland, USA: Macro International Inc.; 1994.
SEN	Senegal	1992	1997	46.3		1	6971	1	Ministère de l'Economie des Finances et du Plan, Macro International Inc. Enquête Démographic et de Santé au Sénégal (EDS-III) 1997. Calverton, Maryland, USA: Macro International Inc.; 1997.
SEN	Senegal	1994	1999	46.4		1	12461	1	Ministère de la Santé, SERDHA et Macro International Inc. Enquête Sénégalaise sur les Indicateurs de Santé, 1999. Calverton, Maryland, USA: SERDHA et Macro International Inc.; 2000.
SEN	Senegal	2000	2005	56.7		1	6927	1	Salif N, Ayad M. Enquête Démographique et de Santé au Sénégal 2005. Calverton, Maryland, USA: Centre de Recherche pour le Développement Humain [Sénégal] et ORC Macro; 2006.

ISO3	Country name	Data coverage period start year	Data coverage period end year	Coverage of early ANC visits (%) (women 15-49 years)	No. of pregnant women (15-49 years) having early ANC visit	Timing of first visit codes: 1=<4 mo; 2=<14 weeks; 3=<15 weeks; 4=<16 weeks; 5=1st trimester; 6=<6 mo; 7<12 weeks; 8<13 weeks; 9<20 weeks; 10<3 mo	Sample size	Source code: 1=survey; 2=adm.; 3= perinatal	Sources
SEN	Senegal	2005	2011	60.7		1	7678	1	Agence Nationale de la Statistique et de la Démographie (ANSD) [Sénégal], ICF International. Enquête Démographique et de Santé à Indicateurs Multiples au Sénégal (EDS-MICS) 2010-2011. Calverton, Maryland, USA: ANSD et ICF International; 2012.
SEN	Senegal	2007	2013	55.9		1	4200	1	Agence Nationale de la Statistique et de la Démographie (ANSD) [Sénégal], ICF International. Enquête Démographique et de Santé Continue (EDS-Continue 2012-2013). Calverton, Maryland, USA: ANSD et ICF International; 2013.
SEN	Senegal	2007	2014	58.5		1	4100	1	Agence Nationale de la Statistique, la Démographie (ANSD) [Sénégal], ICF International. Sénégal : Enquête Démographique et de Santé Continue (EDS-Continue 2014). Rockville, Maryland, USA: ANSD et ICF International; 2015.
SRB	Serbia	2012	2014	94.5		5	376	1	Statistical Office of the Republic of Serbia and UNICEF. Serbia Multiple Indicator Cluster Survey and Serbia Roma Settlements Multiple Indicator Cluster Survey, 2014, Final Reports. Belgrade, Serbia: Statistical Office of the Republic of Serbia and UNICEF; 2014.
SLE	Sierra Leone	2003	2008	30		1	4103	1	Statistics Sierra Leone (SSL), ICF Macro. Sierra Leone Demographic and Health Survey 2008. Calverton, Maryland, USA: Statistics Sierra Leone (SSL), ICF Macro; 2009.
SLE	Sierra Leone	2008	2013	44.5		1	8647	1	Statistics Sierra Leone (SSL), ICF International. Sierra Leone Demographic and Health Survey 2013. Freetown, Sierra Leone, Rockville, Maryland, USA: SSL and ICF International; 2014.
SVK	Slovakia	2004	2004	79.5		3	51968	2	EURO-PERISTAT Project, with SCPE, EUROCAT, EURONEOSTAT. European Perinatal Health Report- data for 2004. 2008.
SVN	Slovenia	2004	2004	91.1		3	17628	2	EURO-PERISTAT Project, with SCPE, EUROCAT, EURONEOSTAT. European Perinatal Health Report- data for 2004. 2008.
SVN	Slovenia	2010	2010	93.7		3	21934	2	EURO-PERISTAT Project with SCPE and EUROCAT. European Perinatal Health Report. The health and care of pregnant women and babies in

ISO3	Country name	Data coverage period start year	Data coverage period end year	Coverage of early ANC visits (%) (women 15-49 years)	No. of pregnant women (15-49 years) having early ANC visit	Timing of first visit codes: 1=<4 mo; 2=<14 weeks; 3=<15 weeks; 4=<16 weeks; 5=1st trimester; 6=<6 mo; 7<12 weeks; 8<13 weeks; 9<20 weeks; 10<3 mo	Sample size	Source code: 1=survey; 2=adm.; 3= perinatal	Sources
									Europe in 2010; 2013.
SLB	Solomon Islands	2001	2007	15.2		1	1799	1	National Statistics Office, Secretariat of the Pacific Community, Macro International Inc. Solomon Islands Demographic and Health Survey 2006-2007. Secretariat of the Pacific Community, Noumea, New Caledonia; 2009.
ZAF	South Africa	1993	1998	62.8		6	4992	1	Department of Health, Medical Research Council, Macro-International. South Africa Demographic and Health Survey 1998. Full Report. Pretoria, South Africa: Department of Health; 2000.
ZAF	South Africa	1998	2003	31.7		1	1859	1	Department of Health, Medical Research Council, ORC Macro. South Africa Demographic and Health Survey 2003. Pretoria, South Africa: Department of Health; 2007.
ZAF	South Africa	2006	2008	46.5		9	644	1	Shisana O, Simbayi LC, Rehle T, Zungu NP, Zuma K, Ngogo N et al., and SABSSM III Implementation Team. South African National HIV Prevalence, Incidence, Behaviour and Communication Survey, 2008: The health of our children. Cape Town: HSRC Press; 2010.
LKA	Sri Lanka	2001	2007	92.4		1	6014	1	Department of Census and Statistics, Ministry of Health Care and Nutrition. Sri Lanka Demographic and Health Survey 2006-2007. Colombo, Sri Lanka; 2008.
SDN	Sudan	1994	1999	44.3		1	11499	1	Ministry of Health, Central Bureau of Statistics, UNPFA. Safe Motherhood Survey National Report - 1999. Khartoum; 2001.
SDN	Sudan	2012	2014	46.5		1	4468	1	Central Bureau of Statistics (CBS), UNICEF Sudan. Sudan Multiple Indicator Cluster Survey 2014. Final Report. Khartoum, Sudan: UNICEF and CBS; 2016.
SWZ	Swaziland	2001	2007	25.8		1	2134	1	Central Statistical Office (CSO) [Swaziland],Macro International Inc. Swaziland Demographic and Health Survey 2006-07. Mbabane, Swaziland: Central Statistical Office and Macro International Inc.; 2008.
SYR	Syrian Arab Republic	1992	1992	41.4		5	5800	1	Ministry of Health, UNICEF. Study on Maternal Health Care Services During Pregnancy and Delivery in Syria - 1992. Damascus; 1993.

ISO3	Country name	Data coverage period start year	Data coverage period end year	Coverage of early ANC visits (%) (women 15-49 years)	No. of pregnant women (15-49 years) having early ANC visit	Timing of first visit codes: 1=<4 mo; 2=<14 weeks; 3=<15 weeks; 4=<16 weeks; 5=1st trimester; 6=<6 mo; 7<12 weeks; 8<13 weeks; 9<20 weeks; 10<3 mo	Sample size	Source code: 1=survey; 2=adm.; 3= perinatal	Sources
SYR	Syrian Arab Republic	1996	2001	87		6	2863	1	League of Arab States Pan Arab Project for Family Health PAPFAM, Syrian Arab Republic Office of the Prime Minister, Central Bureau of Statistics. The Family Health Survey in the Syrian Arab Republic 2001. Syrian Arab Republic; 2002.
THA	Thailand	2008	2009	71.3		7	37511	1	National Statistical Office Thailand, Ministry of Health and UNPFA. Key Findings: The 2009 Reproductive Health Survey Thailand. Bangkok, Thailand; 2010.
TLS	Timor-Leste	1998	2003	23.8		1	3338	1	Ministry of Health and National Statistics Office, Timor-Leste, and University of Newcastle, The Australian National University, ACIL Australia Pty Ltd, Australia. Timor-Leste 2003 Demographic and Health Survey. University of Newcastle, Newcastle, Australia; 2004.
TLS	Timor-Leste	2004	2010	45.3		1	6015	1	National Statistics Directorate (NSD) [Timor-Leste], Ministry of Finance [Timor-Leste], ICF Macro. Timor-Leste Demographic and Health Survey 2009-10. Dili, Timor-Leste: NSD [Timor-Leste] and ICF Macro; 2010.
TGO	Togo	1995	1998	16.8		1	3978	1	Kodjo A, Mboup G, Ouro-Gnao AM, Boukpessi B, Messan PA, Salami-Odjo R. Enquête Démographic et de Santé, Togo 1998. Calverton, Maryland USA: Direction de la Statistique et Macro International Inc.; 1999.
TGO	Togo	2008	2014	27.7		1	4858	1	Ministère de la Planification, du Développement et de l'Aménagement du Territoire (MPDAT), Ministère de la Santé (MS), ICF International. Enquête Démographique et de Santé au Togo 2013-2014. Rockville, Maryland, USA : MPDAT, MS et ICF International; 2015.
TON	Tonga	2007	2012	21.3		1	1069	1	Tonga Department of Statistics and Tonga Ministry of Health, SPC and UNFPA. Tonga Demographic and Health Survey, 2012. Tonga; 2013.
TUN	Tunisia	1989	1995	16.7	57	6	341	1	Ministère de la Santé Publique. L'Enquete Tunisienne sur la Santé de la Mére et de l'Enfant 1994-1995 (ETSME). Rapport Principal. Tunesie; 1996.

ISO3	Country name	Data coverage period start year	Data coverage period end year	Coverage of early ANC visits (%) (women 15-49 years)	No. of pregnant women (15-49 years) having early ANC visit	Timing of first visit codes: 1=<4 mo; 2=<14 weeks; 3=<15 weeks; 4=<16 weeks; 5=1st trimester; 6=<6 mo; 7<12 weeks; 8<13 weeks; 9<20 weeks; 10<3 mo	Sample size	Source code: 1=survey; 2=adm.; 3= perinatal	Sources
TUR	Turkey	1998	1993	45.7		1	3700	1	Ministry of Health [Turkey], Hacettepe University Institute of Population Studies, Macro International Inc. Turkish Demographic and Health Survey 1993. Ankara, Turkey; 1994.
TUR	Turkey	1993	1998	51.5		1	3459	1	Hacettepe University, Institute of Population Studies Ankara, Turkey, Macro Inc. Turkish Demographic and Health Survey 1998. International Inc. Macro International Inc. Calverton, Maryland, USA; 1999.
TUR	Turkey	1998	2003	57.5		1	3164	1	Hacettepe University Institute of Population Studies. Turkey Demographic and Health Survey, 2003. Hacettepe University Institute of Population Studies, Ministry of Health General Directorate of Mother and Child Health and Family Planning, State Planning Organization and European Union. Ankara, Turkey; 2004.
TUR	Turkey	2003	2008	74.2		1	2768	1	Hacettepe University Institute of Population Studies. Turkey Demographic and Health Survey, 2008. Hacettepe University Institute of Population Studies, Ministry of Health General Directorate of Mother and Child Health and Family Planning, T.R. Prime Ministry Undersecretary of State Planning Organization and TÜBİTAK, Ankara, Turkey; 2009.
TUR	Turkey	2008	2013	89.9		1	2672	1	Hacettepe University Institute of Population Studies. Turkey Demographic and Health Survey 2013. Hacettepe University Institute of Population Studies, T.R. Ministry of Development and TÜBİTAK, Ankara, Turkey; 2014.
TKM	Turkmenistan	1995	2000	72.4		1	2470	1	Gurbansoltan Eje Clinical Research Center for Maternal and Child Health (GECRCMCH), Ministry of Health and Medical Industry [Turkmenistan], ORC Macro. Turkmenistan Demographic and Health Survey 2000. Calverton, Maryland, USA: GECRCMCH and ORC Macro; 2001.
TUV	Tuvalu	2002	2007	27.3		1	292	1	Central Statistics Division(TCSD), SPC and Macro International Inc. Tuvalu Demographic and Health Survey 2007. Noumea, New Caledonia; 2009.
UGA	Uganda	1992	1995	14.3		1	6027	1	Statistics Department [Uganda], Macro International Inc. Uganda Demographic and Health Survey, 1995. Calverton, Maryland: Statistics Department [Uganda]

ISO3	Country name	Data coverage period start year	Data coverage period end year	Coverage of early ANC visits (%) (women 15-49 years)	No. of pregnant women (15-49 years) having early ANC visit	Timing of first visit codes: 1=<4 mo; 2=<14 weeks; 3=<15 weeks; 4=<16 weeks; 5=1st trimester; 6=<6 mo; 7<12 weeks; 8<13 weeks; 9<20 weeks; 10<3 mo	Sample size	Source code: 1=survey; 2=adm.; 3= perinatal	Sources
									and Macro International Inc.; 1996.
UGA	Uganda	1995	2001	14.4		1	4489	1	Uganda Bureau of Statistics (UBOS), ORC Macro. Uganda Demographic and Health Survey 2000-2001. Calverton, Maryland, USA: UBOS and ORC Macro; 2001.
UGA	Uganda	2001	2006	16.6		1	5035	1	Uganda Bureau of Statistics (UBOS), Macro International Inc. Uganda Demographic and Health Survey 2006. Calverton, Maryland, USA: UBOS and Macro International Inc.;2007.
UGA	Uganda	2006	2011	20.8		1	4968	1	Uganda Bureau of Statistics (UBOS), ICF International Inc. Uganda Demographic and Health Survey 2011. Kampala, Uganda: UBOS and Calverton, Maryland: ICF International Inc.;2012.
UKR	Ukraine	1994	1999	65.2		1	1938	1	Kiev International Institute of Sociology, CDC, USAID. 1999 Ukraine Reproductive Health Survey, Final Report. Ukraine; 2001.
UKR	Ukraine	2002	2007	83.6		1	1072	1	Ukrainian Center for Social Reforms (UCSR), State Statistical Committee (SSC) [Ukraine], Ministry of Health (MOH) [Ukraine], Macro International Inc. Ukraine Demographic and Health Survey 2007. Calverton, Maryland, USA: UCSR and Macro International; 2008.
GBR	United Kingdom/Scotland	2014	2014	90.8	48305	3	53222	2	ISD Scotland. Information Services Division, NHS National Services Scotland. Maternity and Births. Edinburgh: ISD Scotland; 2016. Available from: http://www.isdscotland.org/Health-Topics/Maternity-and-Births/Publications/index.asp#
GBR	United Kingdom/Scotland	2013	2013	88.1	48908	3	55542	2	ISD Scotland. Information Services Division, NHS National Services Scotland. Maternity and Births. Edinburgh: ISD Scotland; 2016. Available from: http://www.isdscotland.org/Health-Topics/Maternity-and-Births/Publications/index.asp#
GBR	United Kingdom/Scotland	2012	2012	79.5	45378	3	57089	2	ISD Scotland. Information Services Division, NHS National Services Scotland. Maternity and Births. Edinburgh: ISD Scotland; 2016. Available from: http://www.isdscotland.org/Health-Topics/Maternity-and-Births/Publications/index.asp#1003

ISO3	Country name	Data coverage period start year	Data coverage period end year	Coverage of early ANC visits (%) (women 15-49 years)	No. of pregnant women (15-49 years) having early ANC visit	Timing of first visit codes: 1=<4 mo; 2=<14 weeks; 3=<15 weeks; 4=<16 weeks; 5=1st trimester; 6=<6 mo; 7<12 weeks; 8<13 weeks; 9<20 weeks; 10<3 mo	Sample size	Source code: 1=survey; 2=adm.; 3= perinatal	Sources
GBR	United Kingdom/Scotland	2011	2011	73.7	41898	3	56843	2	ISD Scotland. Information Services Division, NHS National Services Scotland. Maternity and Births. Edinburgh: ISD Scotland; 2016. Available from: http://www.isdscotland.org/Health-Topics/Maternity-and-Births/Publications/index.asp#1003
GBR	United Kingdom/England	1995	1995	82		7	2406	3	Redshaw M, Rowe R, Hockley C, Brocklehurst P, National Perinatal Epidemiology Unit (NPEU), University of Oxford. Recorded delivery: a national survey of women's experience of maternity care 2006. Oxford: NPEU; 2007.
GBR	United Kingdom/England	2006	2006	86		7	2960	3	Redshaw M, Rowe R, Hockley C, Brocklehurst P, National Perinatal Epidemiology Unit (NPEU), University of Oxford. Recorded delivery: a national survey of women's experience of maternity care 2006. Oxford: NPEU; 2007.
GBR	United Kingdom/England	2007	2007	79		8	24646	3	Care Quality Commission, NHS. 2010 survey of women's experiences of maternity care. Statistical release. England UK: Care Quality Commission; 2010.
GBR	United Kingdom/England	2010	2010	90		7	5268	3	Redshaw M, Heikkila K, National Perinatal Epidemiology Unit (NPEU), University of Oxford. Recorded delivery: a national survey of women's experience of maternity care 2010. Oxford: NPEU; 2010.
GBR	United Kingdom/England	2010	2010	89		8	23154	3	Care Quality Commission, NHS. 2010 survey of women's experiences of maternity care. Statistical release. England UK: Care Quality Commission; 2010.
GBR	United Kingdom/England	2013	2013	82		8	21614	3	Care Quality Commission. National findings from the 2013 survey of women's experiences of maternity care. England UK: Care Quality Commission; 2014.
GBR	United Kingdom/England	2014	2014	96		8	9999	3	Redshaw M, Henderson J, National Perinatal Epidemiology Unit (NPEU), University of Oxford. Recorded delivery: a national survey of women's experience of maternity care 2014. Oxford: NPEU; 2015.
GBR	United Kingdom/England	2015	2015	96		8	20631	3	Care Quality Commission, NHS. 2015 survey of women's experiences of maternity care. Staticital release. England UK: Care Quality Commission;

ISO3	Country name	Data coverage period start year	Data coverage period end year	Coverage of early ANC visits (%) (women 15-49 years)	No. of pregnant women (15-49 years) having early ANC visit	Timing of first visit codes: 1=<4 mo; 2=<14 weeks; 3=<15 weeks; 4=<16 weeks; 5=1st trimester; 6=<6 mo; 7<12 weeks; 8<13 weeks; 9<20 weeks; 10<3 mo	Sample size	Source code: 1=survey; 2=adm.; 3= perinatal	Sources
									2015.
TZA	United Republic of Tanzania	1986	1992	12.1		1	8032	1	Bureau of Statistics Planning Commission, Macro International Inc. Tanzania Demographic and Health Survey 1991/1992. Columbia, Maryland USA; 1993.
TZA	United Republic of Tanzania	1991	1996	11.6		1	6916	1	Bureau of Statistics [Tanzania], Macro International Inc. Tanzania Demographic and Health Survey 1996. Calverton, Maryland: Bureau of Statistics and Macro International; 1997.
TZA	United Republic of Tanzania	1994	1999	10.5		1	2183	1	National Bureau of Statistics [Tanzania] and Macro International Inc. Tanzania Reproductive and Child Health Survey 1999. Calverton, Maryland, USA: National Bureau of Statistics and Macro International Inc.; 2000.
TZA	United Republic of Tanzania	1999	2005	14.1		1	5628	1	National Bureau of Statistics (NBS) [Tanzania], ORC Macro. Tanzania Demographic and Health Survey 2004-05. Dar es Salaam, Tanzania: National Bureau of Statistics and ORC Macro; 2005.
TZA	United Republic of Tanzania	2005	2010	15		1	5378	1	National Bureau of Statistics (NBS) [Tanzania], ICF Macro. Tanzania Demographic and Health Survey 2010. Dar es Salaam, Tanzania: NBS and ICF Macro; 2011.
USA	United States of America	1990	1990	75.8		5	4158212	2	Centers for Disease Control and Prevention (CDC), National Center for Health Statistics. Prenatal Care in the United States 1980-1994. Series 21. No. 54. Hyattsville, Maryland: CDC; 1996.
USA	United States of America	1991	1991	76.2		5	4110907	2	Centers for Disease Control and Prevention (CDC), National Center for Health Statistics. Prenatal Care in the United States 1980-1994. Series 21. No. 54. Hyattsville, Maryland: CDC; 1996.
USA	United States of America	1992	1992	77.7		5	4065014	2	Centers for Disease Control and Prevention (CDC), National Center for Health Statistics. Prenatal Care in the United States 1980-1994. Series 21. No. 54. Hyattsville, Maryland: CDC; 1996.
USA	United States of America	1993	1993	78.9		5	4000240	2	Centers for Disease Control and Prevention (CDC), National Center for Health Statistics. Prenatal Care in the United States 1980-1994. Series 21. No. 54. Hyattsville, Maryland: CDC; 1996.

ISO3	Country name	Data coverage period start year	Data coverage period end year	Coverage of early ANC visits (%) (women 15-49 years)	No. of pregnant women (15-49 years) having early ANC visit	Timing of first visit codes: 1=<4 mo; 2=<14 weeks; 3=<15 weeks; 4=<16 weeks; 5=1st trimester; 6=<6 mo; 7<12 weeks; 8<13 weeks; 9<20 weeks; 10<3 mo	Sample size	Source code: 1=survey; 2=adm.; 3= perinatal	Sources
USA	United States of America	1994	1994	80.2		5	3952767	2	Centers for Disease Control and Prevention (CDC), National Center for Health Statistics. Prenatal Care in the United States 1980-1994. Series 21. No. 54. Hyattsville, Maryland: CDC; 1996.
USA	United States of America	1995	1995	81.3		5	3899589	2	Martin JA, Hamilton BE, Ventura SJ, Menacker F, Park MM, Sutton PD. Births: Final data for 2001. National vital statistics reports; vol. 51 no. 2. Hyattsville, Maryland: National Center for Health Statistics; 2002.
USA	United States of America	1996	1996	81.9		5	3891494	2	Martin JA, Hamilton BE, Ventura SJ, Menacker F, Park MM, Sutton PD. Births: Final data for 2001. National vital statistics reports; vol. 51 no. 2. Hyattsville, Maryland: National Center for Health Statistics; 2002.
USA	United States of America	1997	1997	82.5		5	3880894	2	Martin JA, Hamilton BE, Ventura SJ, Menacker F, Park MM, Sutton PD. Births: Final data for 2001. National vital statistics reports; vol. 51 no. 2. Hyattsville, Maryland: National Center for Health Statistics; 2002.
USA	United States of America	1998	1998	82.8		5	3941553	2	Martin JA, Hamilton BE, Ventura SJ, Menacker F, Park MM, Sutton PD. Births: Final data for 2001. National vital statistics reports; vol. 51 no. 2. Hyattsville, Maryland: National Center for Health Statistics; 2002.
USA	United States of America	1999	1999	83.2		5	3959417	2	Martin JA, Hamilton BE, Ventura SJ, Menacker F, Park MM, Sutton PD. Births: Final data for 2001. National vital statistics reports; vol. 51 no. 2. Hyattsville, Maryland: National Center for Health Statistics; 2002.
USA	United States of America	2000	2000	83.2		5	4058814	2	Martin JA, Hamilton BE, Ventura SJ, Menacker F, Park MM, Sutton PD. Births: Final data for 2001. National vital statistics reports; vol. 51 no. 2. Hyattsville, Maryland: National Center for Health Statistics; 2002.
USA	United States of America	2001	2001	83.4		5	4025933	2	Martin JA, Hamilton BE, Ventura SJ, Menacker F, Park MM, Sutton PD. Births: Final data for 2001. National vital statistics reports; vol. 51 no. 2. Hyattsville, Maryland: National Center for Health Statistics; 2002.

ISO3	Country name	Data coverage period start year	Data coverage period end year	Coverage of early ANC visits (%) (women 15-49 years)	No. of pregnant women (15-49 years) having early ANC visit	Timing of first visit codes: 1=<4 mo; 2=<14 weeks; 3=<15 weeks; 4=<16 weeks; 5=1st trimester; 6=<6 mo; 7<12 weeks; 8<13 weeks; 9<20 weeks; 10<3 mo	Sample size	Source code: 1=survey; 2=adm.; 3= perinatal	Sources
USA	United States of America	2002	2002	83.7		5	4021726	2	Martin JA, Hamilton BE, Sutton PD, et al. Births: Final data for 2003. National vital statistics reports; vol. 54 no. 2. Hyattsville, MD: National Center for Health Statistics; 2005.
USA	United States of America	2003	2003	84.1		5	4089950	2	Martin JA, Hamilton BE, Sutton PD, et al. Births: Final data for 2003. National vital statistics reports; vol. 54 no. 2. Hyattsville, MD: National Center for Health Statistics; 2005.
USA	United States of America	2007	2007	70.8		5	4316233	2	CDC/NCHS, National Vital Statistics System, Birth File. Martin JA, Hamilton BE, Sutton PD, Ventura SJ, Mathews TJ, Osterman MJK. Births: Final data for 2008. National vital statistics reports; vol 59 no 1. Hyattsville, MD: NCHS; 2010.
USA	United States of America	2008	2008	70.7		5	4247694	2	CDC/NCHS, National Vital Statistics System, Birth File. Martin JA, Hamilton BE, Sutton PD, Ventura SJ, Mathews TJ, Osterman MJK. Births: Final data for 2008. National vital statistics reports; vol 59 no 1. Hyattsville, MD: NCHS; 2010.
UZB	Uzbekistan	1993	1996	72.6		1	1392	1	Institute of Obstetrics and Gynecology [Uzbekistan], Macro International Inc. Uzbekistan Demographic and Health Survey, 1996. Calverton, Maryland: Institute of Obstetrics and Gynecology and Macro International Inc.; 1997.
VUT	Vanuatu	2008	2013	30.1		1	1139	1	Vanuatu National Statistics Office (VNSO) and (Secretariat of the Pacific Community (SPC). Vanuatu Demographic and Health Survey, 2013. SPC; Noumea, New Caledonia; 2014.
VEN	Venezuela (Bolivarian Republic of)	1993	1998	78.1		5	2545063	1	FNUAP. Encuesta Nacional de Población y Familia 1998. Venezuela; 2001.
VNM	Viet Nam	1994	1997	43		1	1818	1	National Committee for Population and Family Planning. The Population and Family Health Project. Viet Nam Demographic and Health Survey 1997. Ha Noi, Viet Nam; 1999.
VNM	Viet Nam	1999	2002	56.7		1	1321	1	Committee for Population, Family and Children [Vietnam], ORC Macro. Vietnam Demographic and Health Survey 2002. Calverton, Maryland, USA: Committee for Population, Family and Children and

ISO3	Country name	Data coverage period start year	Data coverage period end year	Coverage of early ANC visits (%) (women 15-49 years)	No. of pregnant women (15-49 years) having early ANC visit	Timing of first visit codes: 1=<4 mo; 2=<14 weeks; 3=<15 weeks; 4=<16 weeks; 5=1st trimester; 6=<6 mo; 7<12 weeks; 8<13 weeks; 9<20 weeks; 10<3 mo	Sample size	Source code: 1=survey; 2=adm.; 3= perinatal	Sources
									ORC Macro; 2003.
VNM	Viet Nam	2012	2014	84.5		1	1464	1	General Statistics Office, UNICEF. Viet Nam Multiple Indicator Cluster Survey 2014, Final Report. Ha Noi, Viet Nam; 2015.
YEM	Yemen	1986	1992	13.5		1	7421	1	Central Statistical Organization (CSO) [Yemen], Pan Arab Project for Child Development (PAPCHILD) [Egypt], Macro International Inc. (MI). Yemen Demographic and Maternal and Child Health Survey 1991/1992. Calverton, Maryland: CSO and MI; 1994.
YEM	Yemen	1992	1997	17		1	12685	1	Central Statistical Organization (CSO) [Yemen], Macro International Inc. (MI). Yemen Demographic and Maternal and Child Health Survey 1997. Calverton, Maryland, USA: CSO and MI; 1998.
YEM	Yemen	1998	2003	67	2249	6	3356	1	The Republic of Yemen Ministry of Health and Population, Central Statistical Organization, League of Arab States. The Yemen Family Health Survey: Principal Report. Pan Arab Project for Family Health. Cairo, Egypt: The Republic of Yemen Ministry of Health and Population, Central Statistical Organization and League of Arab States; 2004.
YEM	Yemen	2008	2013	30.6		1	10369	1	Ministry of Public Health and Population (MOPHP), Central Statistical Organization (CSO) [Yemen], Pan Arab Program for Family Health (PAPFAM), ICF International. Yemen National Health and Demographic Survey 2013. Rockville, Maryland, USA: MOPHP, CSO, PAPFAM, and ICF International; 2015.
ZMB	Zambia	1987	1992	9.7		1	6211	1	Central Statistical Office [Zambia], University of Zambia, Macro International Inc. Zambia Demographic and Health Survey, 1992. Macro International Inc., Columbia, Maryland, USA; 1993.
ZMB	Zambia	1991	1996	11		1	7159	1	Central Statistical Office [Zambia] and Ministry of Health, Macro International Inc. Zambia Demographic and Health Survey, 1996. Calverton, Maryland: Central Statistical Office and Macro International Inc.; 1997.

ISO3	Country name	Data coverage period start year	Data coverage period end year	Coverage of early ANC visits (%) (women 15-49 years)	No. of pregnant women (15-49 years) having early ANC visit	Timing of first visit codes: 1=<4 mo; 2=<14 weeks; 3=<15 weeks; 4=<16 weeks; 5=1st trimester; 6=<6 mo; 7<12 weeks; 8<13 weeks; 9<20 weeks; 10<3 mo	Sample size	Source code: 1=survey; 2=adm.; 3= perinatal	Sources
ZMB	Zambia	1996	2002	14.3		1	4402	1	Central Statistical Office [Zambia], Central Board of Health [Zambia], ORC Macro. Zambia Demographic and Health Survey 2001-2002. Calverton, Maryland, USA: Central Statistical Office, Central Board of Health, and ORC Macro; 2003.
ZMB	Zambia	2002	2007	19.2		1	4136	1	Central Statistical Office (CSO), Ministry of Health (MOH), Tropical Diseases Research Centre (TDRC), University of Zambia, Macro International Inc. Zambia Demographic and Health Survey 2007. Calverton, Maryland, USA: CSO and Macro International Inc.; 2009.
ZMB	Zambia	2008	2014	24.4		1	9324	1	Central Statistical Office (CSO) [Zambia], Ministry of Health (MOH) [Zambia], ICF International. Zambia Demographic and Health Survey 2013-14. Rockville, Maryland, USA: Central Statistical Office, Ministry of Health, and ICF International; 2014.
ZWE	Zimbabwe	1991	1994	25.2		1	2328	1	Central Statistical Office [Zimbabwe], Macro International Inc. Zimbabwe Demographic and Health Survey, 1994. Calverton, Maryland, USA: Central Statistical Office and Macro International Inc.; 1995.
ZWE	Zimbabwe	1994	1999	26.7		1	2770	1	Central Statistical Office [Zimbabwe], Macro International Inc. Zimbabwe Demographic and Health Survey 1999. Calverton, Maryland, USA: Central Statistical Office and Macro International Inc.; 2000.
ZWE	Zimbabwe	2000	2006	27.2		1	4099	1	Central Statistical Office (CSO) [Zimbabwe], Macro International Inc. Zimbabwe Demographic and Health Survey 2005-06. Calverton, Maryland, USA: CSO and Macro International Inc.; 2007.
ZWE	Zimbabwe	2007	2009	31.4		1	2799	1	Central Statistical Office (CSO) [Zimbabwe], UNICEF. Multiple Indicator Monitoring Survey (MIMS) 2009. CSO: Harare, Zimbabwe; 2010.
ZWE	Zimbabwe	2005	2011	19.4		1	4426	1	Zimbabwe National Statistics Agency (ZIMSTAT), ICF International. Zimbabwe Demographic and Health Survey 2010-11. Calverton, Maryland: ZIMSTAT and ICF International Inc.; 2012.

ISO3	Country name	Data coverage period start year	Data coverage period end year	Coverage of early ANC visits (%) (women 15-49 years)	No. of pregnant women (15-49 years) having early ANC visit	Timing of first visit codes: 1=<4 mo; 2=<14 weeks; 3=<15 weeks; 4=<16 weeks; 5=1st trimester; 6=<6 mo; 7<12 weeks; 8<13 weeks; 9<20 weeks; 10<3 mo	Sample size	Source code: 1=survey; 2=adm.; 3= perinatal	Sources
ZWE	Zimbabwe	2012	2014	31.2		1	2615	1	Zimbabwe National Statistics Agency (ZIMSTAT). Multiple Indicator Cluster Survey 2014, Key Findings. Harare, Zimbabwe: ZIMSTAT; 2014.
